# Effects of Virtual Reality Interventions on Balance in Community-Dwelling Older Adults: A Systematic Review

**DOI:** 10.3390/jcm15218368

**Published:** 2026-10-27

**Authors:** YongGu Han, SunWook Park

**Affiliations:** 1 Department of Physical Therapy, Shinsung University, Dangjin 31801, Chungcheongnam-do, Republic of Korea; inr5678@naver.com; 2 Department of Physical Therapy, Kangwon National University, Samcheok 25949, Gangwon-do, Republic of Korea

**Keywords:** balance, community-dwelling, older people, virtual reality, systematic review

## Abstract

**Objectives:** This systematic review compared the effects of virtual reality (VR) interventions on balance in community-dwelling older adults with those of inactive and active comparators. **Methods:** PubMed, PEDro, CINAHL and Embase were searched from inception to January 2025, and the PubMed search was updated on 8 October 2026 for randomised controlled trials (RCTs) of VR interventions in ambulatory community-dwelling older adults with a mean age of 60 years or above in every study arm. Risk of bias was assessed with RoB 2. Because comparators, outcome instruments and available statistics were too heterogeneous for meta-analysis, findings were synthesised by comparator type and outcome domain using the direction of the between-group effect on 1 prespecified outcome per trial and domain, irrespective of statistical significance, and the certainty of evidence was rated with GRADE. **Results:** Thirty-five reports of 33 RCTs were included, 15 of them identified in the updated search; overall risk of bias was low in three trials, raised some concerns in 20 and was high in 10. Forty-eight further reports from the updated search are awaiting classification. Against inactive comparators, the prespecified clinical balance outcome favoured VR with a clear between-group difference in six of 17 comparisons; all in trials with some concerns regarding the risk of bias and none of the three trials at low risk of bias showed a clear difference. Against active comparators of similar dose, clear differences occurred in both directions (two favouring VR and two favouring the comparator, out of 13), so no consistent advantage for VR was shown; the trials were small and rarely reported effect estimates with confidence intervals, so equivalence could not be established. Four trials reported falls; in the largest, at low risk of bias, home-based stepping exergames reduced the rate of falls compared with an education control (incidence rate ratio 0.74, 95% confidence interval 0.56–0.98). A sensitivity analysis restricted to trials meeting the registered age criterion did not change these findings. **Conclusions:** VR-based training may improve balance compared with no training, and home-based stepping exergames probably reduce falls. There is no evidence that VR is superior to conventional balance training of comparable dose, but the available trials cannot show whether it is equivalent, and the certainty of the balance evidence is low to very low. VR can be considered an alternative format for delivering balance training, pending adequately powered comparative trials.

## 1. Introduction

Changes in balance and an increased risk of falls are important health concerns in the elderly population [1]. One-third of community-dwelling older adults aged ≥ 65 years experience a bruise or fracture from a fall each year, and after the age of 60 years, they experience a decline in physical function, trunk and lower extremity muscle strength and bone density, resulting in decreased balance and an increased fear of falling [2]. Previous studies have shown that 28.7% of older adults experience at least one fall per year, and 37.5% of older adults who experience a fall require medical treatment or experience activity limitations [3]. Therefore, it is important to investigate balance problems associated with falls in community-dwelling older adults and create alternatives to reduce the incidence of falls; exercise programmes that challenge balance are currently the best-supported of these alternatives [4].

Currently, various interventions are used in the community to improve balance and prevent falls among older adults. In addition to standard physical therapy, dance sports programmes, Pilates, and training with tools, such as gym balls, have been used to improve balance [5,6]. However, current physical therapy interventions to improve balance often require the same interventions to be performed repeatedly during each treatment, resulting in monotony among patients [7]. To overcome these shortcomings, computer-based virtual reality training programmes have become increasingly popular as effective and inexpensive treatments that can be applied at home or in the community. In addition to traditional interventions, virtual reality therapy has been widely used in stroke, cerebral palsy, and sports rehabilitation [8,9,10]. In addition, virtual reality tasks have been reported to be effective in improving static and dynamic balance and coordination skills in games, sports, and dance [10].

Although VR programmes are increasingly used to improve balance, the trials evaluating them in older adults are generally small and differ widely in platform, dose and comparator. Most systematic reviews of VR for balance have addressed people with stroke, cerebral palsy, Parkinson’s disease or other neurological conditions [9,10,11,12], and their findings cannot be directly extrapolated to community-dwelling older adults without such diagnoses. A previous meta-analysis in community-dwelling older adults pooled VR comparisons and reported more favourable mobility and balance outcomes with VR than with either no intervention or conventional exercise, but rated the included trials as being at high risk of bias with variable protocols and judged the evidence to be inconclusive [13]. We also previously reported a systematic review and meta-analysis of VR and motor function in community-dwelling older adults [14]. That review addressed motor function as a whole and pooled the trials into overall estimates; it was not designed to show whether any benefit of VR on balance reflects an advantage over conventional balance training or only over no training. Whether VR offers any advantage over conventional balance training, as opposed to no training, therefore remains uncertain. This distinction is clinically important, because the practical decision is usually whether to use VR instead of, or in addition to, conventional exercise.

Therefore, this systematic review aimed to synthesise RCTs of VR interventions in community-dwelling older adults and to compare their effects on balance with those of inactive and active comparators, separately for each balance domain, counting each trial once and rating the certainty of each comparator-specific conclusion with GRADE. The review was designed to answer the question that a pooled estimate across all control conditions cannot: whether VR adds anything beyond the generic effect of structured exercise.

## 2. Materials and Methods

This systematic review was conducted and reported in accordance with the PRISMA 2020 statement [15] and the Cochrane Handbook for Systematic Reviews of Interventions [16]. The protocol was registered in PROSPERO on 20 July 2023 (CRD42023451977). A separate meta-analysis of motor function outcomes, registered under a different PROSPERO record (CRD42023417655), has been published [14]. The present report addresses balance outcomes specifically, stratified by comparator type, and draws on an overlapping set of trials. It differs from the earlier report in its outcome focus (balance domains only), unit of analysis (trial rather than report), synthesis method (comparator-stratified synthesis without meta-analysis), risk-of-bias assessment (RoB 2, re-assessed at the domain level for this report) and certainty assessment (GRADE). Deviations from the registered protocol are reported in Section 2.8. The completed PRISMA 2020 checklist is provided in Supplementary Table S3.

### 2.1. Source Data and Search Strategy

PubMed (including MEDLINE), PEDro, CINAHL and Embase were searched from inception; searches were first run in July 2023 and last run in all 4 databases in January 2025. Because more than 18 months had elapsed between that search and the preparation of this revision, the PubMed search was re-run from inception on 8 October 2026 to identify trials published since the last search and to check the completeness of the original search. For this update, the population block was also corrected: the PubMed strategy reported in the original submission contained frailty-specific population terms that were inconsistent with the eligibility criteria, and the update used the general population block shown below. Embase, CINAHL and PEDro were not re-searched in this update. No publication-date limits were applied, and only reports published in English were eligible. The search combined controlled vocabulary (MeSH or Emtree) and free-text terms for older adults and for VR or exergaming. The PubMed strategy used in the updated search was: (“Aged” [MeSH] OR “older adult*” [tiab] OR elderly [tiab] OR “community-dwelling” [tiab]) AND (“Virtual Reality” [MeSH] OR “virtual reality” [tiab] OR “video game*” [tiab] OR exergam* [tiab] OR “Wii Fit” [tiab] OR Nintendo [tiab] OR Xbox [tiab] OR Kinect [tiab] OR “virtual world” [tiab]) AND English [lang]. The search dates and numbers of records retrieved in each run are provided in Supplementary Table S1. Two reviewers (a professor of physiotherapy and a doctoral candidate) independently performed the search and screening, and discrepancies were resolved through discussion. In the updated search, title and abstract screening was assisted by keyword filtering for VR, older adults, balance and trial design, and the remaining records were assessed against the same eligibility criteria. Reports confirmed as eligible from the full text were extracted, assessed for risk of bias and incorporated into the synthesis using the same methods as those applied in the trials from the original searches. Reports whose eligibility could not be confirmed because the full text could not be obtained or the information available was insufficient, are reported as studies awaiting classification (Supplementary Table S5), following Cochrane guidance for updated reviews [16]. The review question was structured using the PICOS framework (Table 1): participants, community-dwelling older adults; intervention, VR; comparison, any non-VR intervention or no intervention; outcome, balance; and study design, RCT.

### 2.2. Study Screening: Inclusion and Exclusion Criteria

Studies were included if they (1) were RCTs, with quasi-randomised designs excluded; (2) enrolled older adults described by the authors as community-dwelling, that is, living independently in the community rather than in residential or institutional care, with a reported mean age of 60 years or above in every study arm; (3) delivered a VR intervention as defined in Section 2.6, either alone or in addition to other exercise; (4) compared it with no intervention, usual care or a non-VR intervention; (5) reported at least 1 balance outcome, including clinical balance or functional mobility, instrumented postural control, balance confidence or fear of falling, or falls; and (6) were published in English. Studies were excluded if they (1) had no control group; (2) enrolled institutionalised older adults; (3) enrolled samples defined by a specific neurological or musculoskeletal diagnosis, such as stroke or Parkinson’s disease; or (4) enrolled samples recruited specifically as frail or pre-frail.

Where 2 reports were judged to describe the same trial (identical group sizes, participant characteristics and intervention protocol), the trial rather than the report was used as the unit of analysis. Two such pairs were identified, Singh et al. [17,18] and Bagheri et al. [19] with Khanmohammadi et al. [20], so 20 reports of 18 unique trials were included from the original searches; each of the 15 trials from the updated search was described in a single report, giving 35 reports of 33 trials in total. Each trial was counted once in all syntheses and is presented in a single row in Table 2, and, for the 2 trials described in companion reports, the more conservative risk-of-bias judgement in each domain was used.

The age criterion registered in PROSPERO was 65 years or above. The criterion applied at screening, however, was a mean age of 60 years or above in every study arm, and this is reported as a protocol deviation (Section 2.8). Five arms from 3 trials had a mean age below 65 years [17,18,21,22]; in the Singh trial, women aged 56 years or above were eligible, although the mean age of both arms exceeded 60 years [17]. One trial reported only an age range of 60 to 94 years [23], and 1 reported only the mean age of all participants (65.7 years) [24]. Because the revised criterion differed from the registered one, all records retrieved by the updated PubMed search, from inception onwards, were screened against the revised criterion, so that any trial excluded earlier on age would be identified. A sensitivity analysis restricted to the 28 trials that met the registered criterion (that is, excluding the trials of Singh et al. [17,18], Kim et al. [21], Barsasella et al. [23], Yu et al. [22] and Zhao et al. [24]) was performed for every comparator-by-domain synthesis.

### 2.3. Risk of Bias Assessment

Because all included studies were RCTs, risk of bias was assessed with version 2 of the Cochrane risk-of-bias tool for randomised trials (RoB 2) [25]. RoB 2 comprises 5 domains: the randomisation process, deviations from intended interventions, missing outcome data, measurement of the outcome and selection of the reported result. The overall judgement (low risk, some concerns or high risk) is derived from the domain-level judgements and is not a separate domain. Each domain was judged using the signalling questions of the tool; the responses for each trial, with the supporting information from the trial reports, are given in Supplementary Table S4. The assessed result was the between-group comparison of the primary balance outcome at the end of the intervention period, and the effect of interest was the effect of assignment to the intervention. In domain 1, the judgement was low only when allocation concealment was explicitly described (for example, central allocation or sequentially numbered, sealed, opaque envelopes); when allocation concealment was not reported, and baseline characteristics did not suggest a problem with randomisation, the domain was judged to raise some concerns, as required by the RoB 2 algorithm [25]. In domain 5, the judgement was low only when a registered protocol or statistical analysis plan was available and the reported balance outcomes and analyses were consistent with it; otherwise, the domain was judged to raise some concerns. The overall judgement was the least favourable domain judgement. For the 15 trials from the updated search, domain 4 was judged to raise some concerns when assessor blinding was not reported for a performance-based outcome, and domain 5 was judged low only when the trial had been registered before enrolment. Domain 4 was judged separately for outcomes whose assessor-blinding arrangements differed within a report. Two reviewers assessed each report independently, and disagreements were resolved through discussion.

### 2.4. Coding of Variables (Data Coding)

Two reviewers independently extracted the following data using a standardised form: authors, year, number of participants randomised and analysed, age, sex, VR platform, comparator, intervention dose (session duration, sessions per week and number of weeks), outcome measures, between-group results with effect estimates and measures of variance where reported, adherence, adverse events and duration of any post-intervention follow-up. Discrepancies were resolved through discussion and by re-checking the original reports. For this revision, every cell of Table 2 was re-extracted from the original trial reports by both reviewers independently and compared cell by cell, and the 15 trials identified in the updated search were extracted with the same form; group sizes are given separately as randomised and as analysed for the balance outcome, companion reports of the same trial are presented in a single row, and only between-group results are reported.

### 2.5. Data Synthesis

A meta-analysis was considered but judged not feasible, and the findings were therefore synthesised without meta-analysis in accordance with the Synthesis Without Meta-analysis (SWiM) reporting guideline [26]. Trials were grouped first by comparator type as either inactive (no intervention, daily routine, daily activity, insole use or usual care) or active (conventional balance-oriented physiotherapy, gym- or group-based exercise, treadmill training or motor–cognitive dual-task training). Trials in which VR was added to the same conventional exercise received by the control group (add-on designs) were identified separately because the VR arm also received a greater total training dose. Within each comparator group, outcomes were grouped into four domains: clinical balance and functional mobility; instrumented postural control; balance confidence and fear of falling; and falls.

Pooling was ruled out for each of the four most frequently reported outcomes. The TUG was reported in seven trials, but the comparators ranged from insoles and no intervention to conventional balance physiotherapy and dual-task training, and no comparator-defined subgroup contained more than four trials, too few to estimate between-study variance reliably [16]. The BBS was reported in only three small trials with five to 11 participants per arm, two of which were three-arm trials comparing VR with different conditions [27,28] and one an explicitly labelled feasibility study [29]. Fear of falling was measured with three instruments that are not interchangeable (FES-I, ABC or ABC-6, and the modified Falls Efficacy Scale), and force-plate outcomes used different variables, recorded under different standing and visual conditions. In addition, between-group differences with measures of variance were not recoverable for a substantial proportion of outcomes, so any pooled analysis would have been restricted to a non-random subset of the evidence.

Because effect estimates could not be combined, the standardised metric was the direction of the between-group effect, as recommended by the Cochrane Handbook when a meta-analysis of effect estimates is not possible [16]. To avoid inflating the number of favourable results, the direction was recorded for one prespecified outcome per trial and domain, selected with a fixed hierarchy: (i) the outcome designated as primary by the trial authors if it belonged to that domain; otherwise, (ii) for clinical balance and functional mobility, the BBS, then the Mini-BESTest, the FAB, the TUG, the 8-foot up-and-go test, the balance component of the SPPB and other clinical tests; (iii) for instrumented postural control, the centre-of-pressure or stepping variable recorded under the most demanding condition; (iv) for balance confidence and fear of falling, the FES-I, then the ABC (or ABC-6) and the modified Falls Efficacy Scale; and (v) for falls, the number of fallers or the fall rate. The direction was classified from the point estimate of the between-group difference at the end of the intervention (or, where only change scores were reported, the difference in change) as favouring VR, favouring the comparator or showing no difference, irrespective of statistical significance; a result without a clear between-group difference whose direction could not be determined from the reported data was classified as showing no clear difference; whether the confidence interval excluded the null value, or the *p* value indicated statistical significance was recorded separately and displayed in an effect direction plot [30]. In a trial with more than one VR arm, a direction was recorded only when it was the same for every VR arm [31]. Within-arm pre–post changes were not used, and effectiveness was not judged by counting statistically significant studies. The direction of effect for each comparison is presented with the overall RoB 2 judgement in Table 3.

### 2.6. Operational Definition and Classification of VR Interventions

For the purpose of this review, a VR intervention was operationally defined as an intervention in which participant movement was captured in real time and mapped onto a computer-generated environment providing concurrent visual feedback and a movement-contingent task goal. Included interventions were classified along two axes: (i) degree of immersion—non-immersive (screen-based with no head-mounted display), semi-immersive (screen-based with additional sensing hardware such as balance boards or motion capture) or fully immersive (head-mounted display with 6-DoF tracking); and (ii) platform type—commercial console exergaming (e.g., Nintendo Wii, Xbox 360/Kinect), proprietary or research-developed VR programmes and VR combined with exercise hardware (e.g., treadmill or stationary bicycle). The classification of each included trial is reported in Section 3.3.

### 2.7. Certainty of Evidence Assessment

The certainty of evidence was rated for each comparator-by-domain synthesis with the GRADE approach [32], using the guidance for rating certainty in the absence of a single pooled effect estimate [33]. Evidence from RCTs started at high certainty and was downgraded for risk of bias, inconsistency, indirectness, imprecision and publication bias; inconsistency explained by risk of bias was not downgraded twice. Because non-significant results from small trials cannot establish the absence of an effect, imprecision was judged against the possibility of a clinically important difference in either direction. The judgements are presented as a summary-of-findings table for each comparator-by-domain synthesis (Table 4).

### 2.8. Deviations from the Protocol

Five deviations from the registered protocol occurred. First, the age criterion was applied as a mean age of 60 years or above in every study arm instead of 65 years or above (Section 2.2); records from the updated search were screened against the revised criterion, and a sensitivity analysis restricted to trials meeting the registered criterion was added. Second, the planned meta-analysis was replaced by a synthesis without meta-analysis for the reasons given in Section 2.5. Third, the trial rather than the report was adopted as the unit of analysis after companion reports were identified. Fourth, a GRADE assessment was added to rate the certainty of the evidence. Fifth, the PubMed search was updated with a population block consistent with the eligibility criteria; eligible trials identified by the update were incorporated, and reports that could not be classified are reported as studies awaiting classification (Section 2.1).

## 3. Results

### 3.1. Search Results

**Figure 1:**
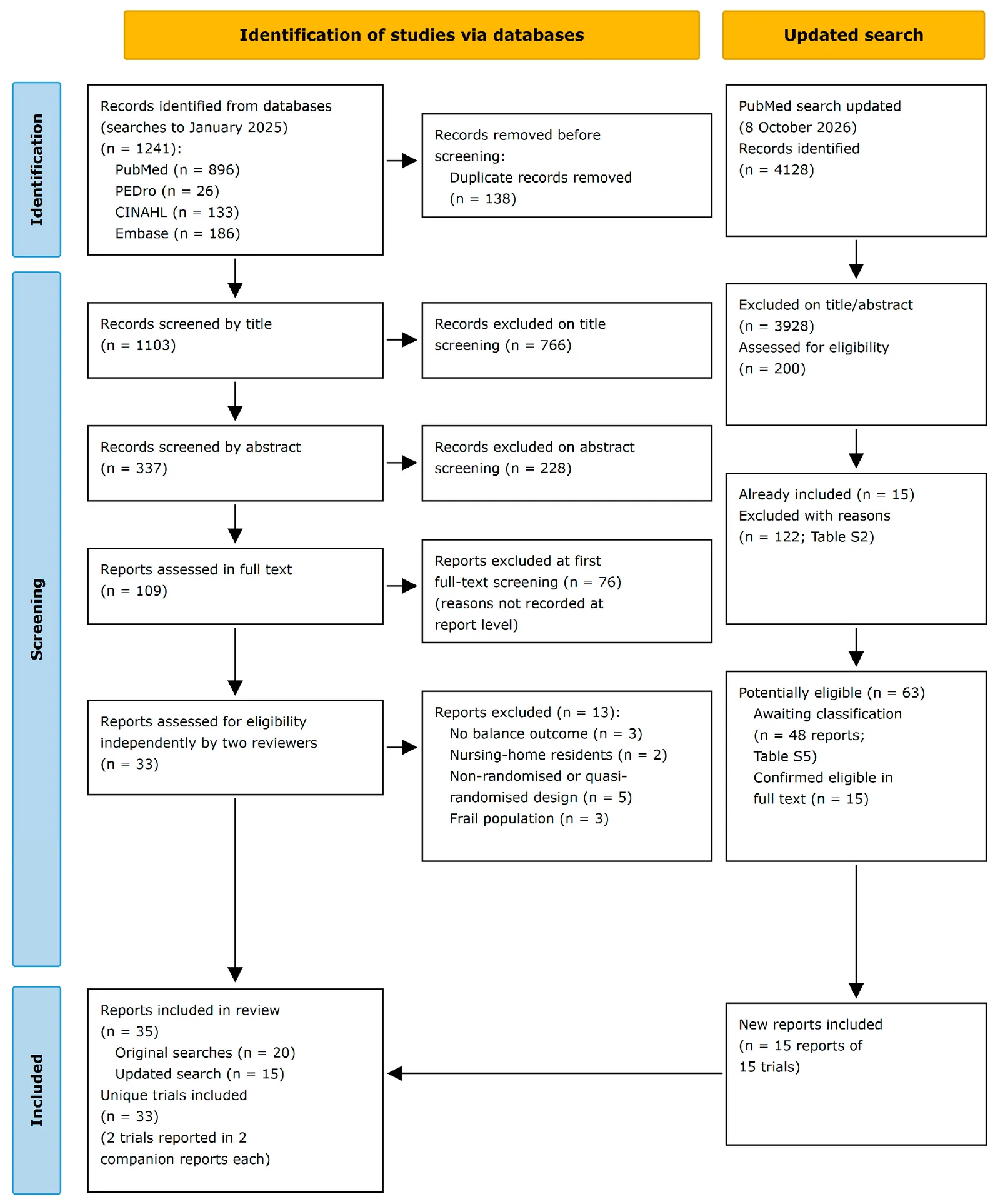
PRISMA flowchart (including the updated PubMed search, 8 October 2026).

A total of 1241 records were identified from the four databases in the searches run to January 2025 (PubMed, 896; PEDro, 26; CINAHL, 133; Embase, 186). After 138 duplicates were removed, 766 records were excluded during title screening and a further 228 after abstract screening. Of the 109 reports retrieved in full text, 76 were excluded at the first full-text screening; the reasons for these exclusions were not recorded at the report level, which is acknowledged as a limitation. The remaining 33 were assessed independently by two reviewers, and 13 were excluded (no balance outcome, *n* = 3; nursing-home residents, n = 2; non-randomised or quasi-randomised design, n = 5; frail population, n = 3), leaving 20 reports of 18 trials. The updated PubMed search (8 October 2026) retrieved 4128 records. After title and abstract screening, 200 reports were assessed for eligibility: 15 corresponded to reports already included, 122 were excluded for the reasons listed in Supplementary Table S2 (for example, non-randomised allocation, residential-care samples, frail or pre-frail samples, samples defined by a specific diagnosis, no balance outcome or no non-VR comparator), and 63 were potentially eligible. Of these, 15 reports of 15 trials were confirmed as eligible in the full text and were incorporated [22,24,31,34,35,36,37,38,39,40,41,42,43,44,45], and 48 could not be classified based on the information available and are listed as studies awaiting classification (Supplementary Table S5). In total, 35 reports of 33 unique trials were included (Figure 1).

### 3.2. Quality Assessment

**Figure 2:**
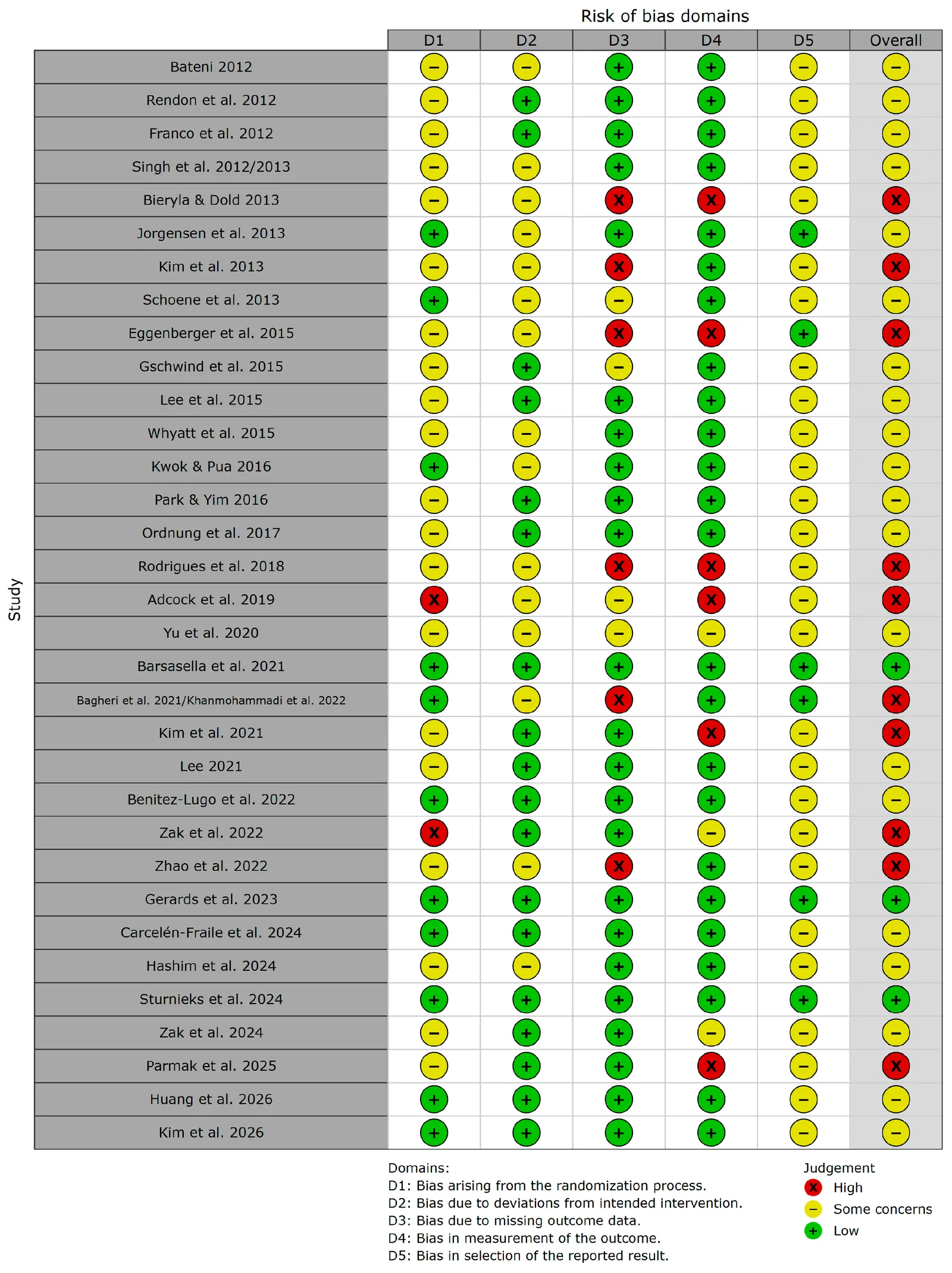
Results of RoB 2.0 (revised judgements; signalling-question responses in Supplementary Table S4).

**Figure 3:**
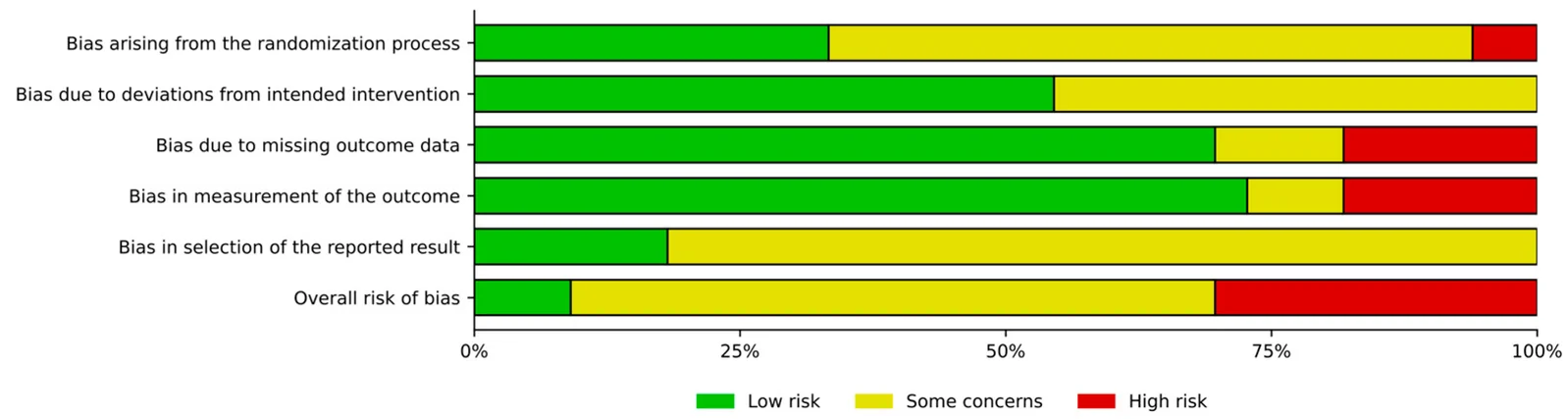
Results of RoB 2.0 (summary plot; revised).

Overall risk of bias was low in three trials [23,40,46], raised some concerns in 20 and was high in 10 (Figure 2 and Figure 3; signalling-question responses and their supporting information are given in Supplementary Table S4). For the randomisation process (domain 1), the 11 trials that described adequate allocation concealment were at low risk [7,19,20,23,34,39,40,42,44,45,46,47], two were at high risk because marked baseline imbalance accompanied unreported allocation concealment [31,37], and the remaining 20 raised some concerns because allocation concealment was not reported. For deviations from intended interventions (domain 2), 18 trials were at low risk and 15 raised some concerns. For missing outcome data (domain 3), 23 trials were at low risk, four raised some concerns and six were at high risk. For measurement of the outcome (domain 4), 24 trials were at low risk, three raised some concerns and 6 were at high risk. For selection of the reported result (domain 5), the six trials with a protocol registered before enrolment and consistent with the report were at low risk [7,19,20,23,40,46,48], and 27 raised some concerns. Among the trials from the original searches, these revisions changed the overall judgement from low risk to some concerns in four trials [27,49,50,51] and from high risk to some concerns in one trial with complete outcome data [47].

### 3.3. Types of Interventions

Of the 33 trials, 19 used commercial console exergaming, namely Nintendo Wii in 12 [7,17,18,19,20,27,28,29,39,42,47,49,52,53], Xbox 360 or Kinect in 6 [22,35,36,50,54,55] and Nintendo Switch in one [24]; two used stepping exergames played on a step pad or mat [34,40]; three used proprietary or research-developed VR programmes [37,48,51]; four combined VR with exercise hardware, a stationary bicycle [21], a treadmill [38,46] or a force platform [44]; one used several VR devices [23]; and four used a head-mounted display [31,41,43,45]. According to the classification in Section 2.6, 29 interventions were non-immersive or semi-immersive and screen-based, and the four head-mounted-display interventions were classified as fully immersive, although two trials used a smartphone-based headset with rotational (3-DoF) tracking only [31,41]; these four trials were small (44 to 80 participants) and two were at high risk of bias, so evidence on contemporary head-mounted platforms remains limited. In two trials, VR was added to the same conventional exercise received by the comparator group (add-on design) [28,51]; in all other trials, VR was delivered alone or in place of part of the comparator programme. Comparators were inactive (no intervention, daily routine, daily activity, usual activities, education, insole use or usual care) or active (conventional balance physiotherapy, the Otago programme, gym-, group- or home-based exercise, treadmill training or motor–cognitive dual-task training). Session duration ranged from 15 to 60 min (1 trial prescribed 120 min per week without a fixed session length [40]), frequency from 1 to 5 sessions per week, and programme length from 3 weeks to 12 months.

### 3.4. Types of Outcomes

Outcomes were grouped into four domains (numbers are trials). Clinical balance and functional mobility were assessed with the TUG (n = 16), the 8-foot up-and-go test (n = 3), the BBS (n = 6), the Fullerton Advanced Balance Scale (n = 3), the SPPB or its balance component (n = 5), single-leg or unipedal stance (n = 6), the Tinetti or Performance-Oriented Mobility Assessment (n = 4), the Functional Reach Test (n = 2) and single-trial instruments (Mini-BESTest, Narrow Corridor Walk Test, Ten Step Test, tandem stance and bubble test). Instrumented postural control was assessed with force-plate or stepping measures (n = 12) and other device-based measures (n = 3). Balance confidence and fear of falling were assessed with the FES-I or Icon-FES (n = 7), the ABC or ABC-6 (n = 3) and the modified Falls Efficacy Scale (n = 1). Falls were reported in 4 trials. Four trials used a head-mounted display [31,41,43,45].

### 3.5. Synthesis of Between-Group Findings

Findings are described by comparator type and outcome domain for one prespecified outcome per trial and domain (Section 2.5), together with the overall RoB 2 judgement of each trial (Table 3); the certainty of each comparator-by-domain synthesis is summarised in Table 4. Within-arm pre–post changes were not used. Twenty-one comparisons used an inactive comparator (no intervention, daily routine, usual activities, education, insole use or usual care). For clinical balance and functional mobility (17 comparisons), the point estimate of the prespecified outcome favoured VR with a clear between-group difference in 6 [7,39,42,45,49,53], all of which raised some concerns about risk of bias; it favoured VR without a clear difference in three [21,22,40]; no clear difference was found in six [23,27,34,37,46,55]; it favoured the comparator without a clear difference in one [35]; and a between-group test was not reported in one [29]. None of the three trials at low overall risk of bias showed a clear between-group difference [23,40,46]. For instrumented postural control (11 comparisons), the prespecified variable favoured VR with a clear difference in four [34,52,53,54] and without a clear difference in two [22,24]; no clear difference was found in three [7,27,36], and the point estimate favoured the comparator in two [35,40]. Because the variables and test conditions differed, these findings are not directly comparable. For balance confidence and fear of falling (six comparisons), the point estimate favoured VR in four, with a clear difference in two [7,49]; no clear difference was found in 1 [34], and the point estimate favoured the comparator in one [35]. Three comparisons against an inactive comparator reported falls. In the largest trial (769 participants, low risk of bias), home-based stepping exergames reduced the rate of falls over 12 months compared with an education booklet (incidence rate ratio 0.74, 95% confidence interval 0.56–0.98) and the proportion of participants who fell (risk ratio 0.75, 0.61–0.92) [40]; one smaller trial showed no clear difference [55], and in one the fall counts favoured VR but were not compared statistically [35].

Fourteen comparisons used an active comparator (conventional balance-oriented physiotherapy, the Otago programme, gym-, group- or home-based exercise, treadmill training or motor–cognitive dual-task training). For clinical balance and functional mobility (13 comparisons), the prespecified outcome showed a clear difference favouring VR in two: an immersive VR programme supervised by a physiotherapist compared with an unsupervised home exercise brochure, at high risk of bias [43], and supervised game-based platform training compared with unsupervised home exercise videos [44]; in both, the VR arm also received more supervision than the comparator. No clear between-group difference was found in 7 [17,18,19,27,47,48,50,53]. The point estimate favoured the comparator in 4, with a clear difference in two: the VR-only arm of a three-arm trial improved less on the BBS than the arm receiving conventional physiotherapy [28], and three head-mounted-display VR arms had slower TUG times than an Otago arm after 3 weeks [31]. Outside the clinical domain, differences favouring VR were confined to the modified Falls Efficacy Scale, without an accompanying difference in TUG [47], selected centre-of-pressure variables during gait initiation [20], and the bubble test and force-plate standing and sitting balance in two add-on comparisons [28,51]. In these add-on designs, VR was given in addition to the conventional exercise received by the control group; because the VR arm also received a greater total training dose, these advantages cannot be attributed to VR itself. For balance confidence and fear of falling (five comparisons), the difference clearly favoured VR in one [47], the point estimate favoured VR without a clear difference in one [43], and no clear difference was found in three [17,19,44]; the only comparison against an active comparator that reported falls showed no clear difference [48].

Across both comparator types, a between-group effect estimate with a confidence interval or other measure of variance was extractable for a minority of prespecified outcomes only; the magnitude and precision of any VR effect therefore remain unquantified, and non-significant results from small trials cannot be interpreted as evidence of no effect or of equivalence. In the sensitivity analysis restricted to the 28 trials meeting the registered age criterion, four inactive comparisons [21,22,23,24] and 1 active comparison [17,18] were removed; the direction of the findings and the certainty ratings were unchanged in every comparator-by-domain synthesis, and two trials at low overall risk of bias remained [40,46].

### 3.6. Follow-Up

Three trials assessed outcomes after the end of the intervention, one at 1 year [48], one after 3 weeks of detraining [53] and one at 3 weeks after the intervention [41], so the durability of any effect remains unknown.

### 3.7. Certainty of Evidence

The certainty of evidence for each comparator-by-domain synthesis is summarised in Table 4. The certainty of evidence was low for clinical balance and functional mobility against inactive comparators (downgraded for risk of bias and imprecision) and very low against active comparators (additionally downgraded for inconsistency because clear differences occurred in both directions). It was very low for instrumented postural control and for balance confidence and fear of falling. For falls, the certainty was moderate against inactive comparators, resting mainly on one large trial of home-based stepping exergames at low risk of bias [40] (downgraded for imprecision), and very low against active comparators (one trial at high risk of bias).

## 4. Discussion

This review synthesised 35 reports of 33 RCTs of VR-based balance training in community-dwelling older adults. Against inactive comparators, the direction of effect on the prespecified clinical balance outcome favoured VR with a clear difference in six of 17 comparisons, all in trials with some concerns about risk of bias, and in none of the three trials at low risk of bias, whereas against active comparators of similar dose, clear differences occurred in both directions and no consistent advantage for VR was shown on the prespecified clinical balance outcome; the certainty of this evidence was low to very low. Improving balance and reducing falls are distinct outcomes, and the evidence assembled here speaks mainly to the former, with the important exception of one large trial at low risk of bias in which home-based stepping exergames reduced falls [40]. Previous reviews reporting benefits of VR on balance concerned mainly people with stroke, Parkinson’s disease, cerebral palsy or other neurological conditions [9,10,11,12]; no trial in the present review enrolled participants with these conditions, and those reviews are cited only for context.

When the evidence is organised by comparator rather than by *p* value, a consistent pattern emerges. Against inactive comparators, every clear between-group difference on a prespecified clinical balance outcome favoured VR, which is what would be expected of any structured, supervised and progressively challenging exercise stimulus relative to no stimulus at all. Against active comparators of broadly similar dose—conventional balance physiotherapy, the Otago programme, gym-, group- or home-based exercise, treadmill training or motor–cognitive dual-task training—clear differences in the prespecified clinical balance outcome occurred in both directions. The two differences that favoured VR came from trials in which the VR training was supervised, whereas the comparator exercise was performed unsupervised at home [43,44], so that supervision rather than VR may explain them; the two differences that favoured the comparator involved conventional physiotherapy and the Otago programme [28,31]. Apart from these, the differences that emerged were confined to single secondary or instrumented measures, and the clearest remaining advantage for VR came from the trial in which it was added to, rather than substituted for, conventional exercise, so that the VR arm also received a greater total training dose [51]. These findings provide no evidence that VR has a specific effect on balance beyond that of an equally dosed and equally supervised exercise stimulus. They are compatible with VR acting as an alternative delivery format, but because the active-comparator trials were small and rarely reported effect estimates with confidence intervals, they cannot establish that VR and conventional training are equivalent, and a clinically relevant difference in either direction remains possible. The motivational and task-specific features of VR have been proposed as the mechanism for its advantage over inactive comparators [10,12]; whether they confer an additional advantage over conventional balance training has not been demonstrated. Divergence across trials therefore corresponded more closely to the nature of the comparator than to the VR platform used. This interpretation differs in emphasis from that of a previous meta-analysis, which reported more favourable outcomes with VR than with both no intervention and conventional exercise but judged the evidence inconclusive because of high risk of bias and variable protocols [13]; the present comparator-stratified synthesis indicates that the favourable findings are concentrated in comparisons against inactive controls and in trials at higher risk of bias. The included trials delivered 15 to 60 min per session (or 120 min per week in the longest trial), one to five sessions per week, over 3 weeks to 12 months. Because no trial compared different doses and this review did not undertake a dose–response analysis, no inference can be drawn about the dose required to alter balance outcomes, and dose remains a question for future trials.

The present findings should also be read alongside our earlier meta-analysis of VR and motor function in community-dwelling older adults, which drew on an overlapping set of trials [14]. That review pooled the trials in a meta-analysis, whereas the present review judged a meta-analysis not to be feasible. The two positions answer different questions rather than reflecting different data. A pooled estimate across all control conditions can be computed, but it answers whether VR is better than whatever the control group happened to receive—a mixture of no training, usual care and structured exercise—which is not the decision a clinician faces. Once the balance evidence is stratified by comparator, as is required to ask whether VR adds anything to conventional training, the trials within each comparator-defined subgroup used different instruments, and between-group estimates with measures of variance are missing for many outcomes (Section 2.5), so that any pooled estimate would rest on a small, non-random subset of the trials. The present review therefore does not replace the earlier pooled estimates but qualifies them: it indicates that a favourable pooled effect of VR on balance is likely to be driven by comparisons against inactive controls and by trials at higher risk of bias, and that the evidence does not show a consistent advantage over conventional balance training of similar dose. With the updated search, it also adds 15 trials published up to 2026, including the first large trial at low risk of bias with falls as its primary outcome [40]. To our knowledge, this is the first review of VR for balance in community-dwelling older adults that separates inactive from active comparators, counts each trial once, records the direction of effect for a single prespecified outcome per domain irrespective of statistical significance and rates the certainty of each comparator-specific conclusion with GRADE. These features change the practical message from “VR improves balance” to “VR is an alternative way to deliver balance training whose superiority over conventional training has not been shown”.

The platforms also differed in the postural control demands they imposed. Weight-shifting platforms, such as the Wii balance board and Kinect-based stepping games used in most included trials, train voluntary displacement of the centre of pressure within the base of support and anticipatory postural adjustments guided by visual feedback [1]. Consistent with the principle of task specificity, the between-group differences observed with these platforms appeared mainly on instrumented centre-of-pressure or stepping variables [20,52,54], which closely resemble the trained task, rather than on global clinical balance scales. Dance-based platforms [48,55] add rhythmic multidirectional stepping and concurrent cognitive demands, whereas treadmill- and cycling-based VR [21,46] mainly challenges locomotor or cyclical lower-limb control; none of these four trials showed a between-group difference on balance outcomes. Because platform type was partly confounded with comparator type and each non-weight-shifting platform was represented by only one or two trials, these observations are hypothesis-generating, but they suggest that the balance effect of VR depends on how closely the trained task matches the balance component being measured.

Most concerns about risk of bias arose in domains 1, 2 and 5. In domain 1, 20 trials raised some concerns because allocation concealment was not reported, and two were at high risk; in domain 2, 15 trials raised some concerns, mainly because participants and intervention providers could not be blinded to a visible exercise intervention and intention-to-treat analyses were seldom reported; and in domain 5, 27 trials raised some concerns because no protocol or statistical analysis plan registered before enrolment was available. A further six trials were at high risk in domain 3 because of missing outcome data and six in domain 4 because of outcome assessment, so that 10 of the 33 trials were at high overall risk and only three were at low overall risk. Importantly, favourable findings on clinical balance were concentrated in trials with some concerns or at high risk, whereas none of the three trials at low overall risk showed a clear between-group difference on the prespecified clinical balance outcome [23,40,46] (Table 3). The low-risk trial that reduced falls did so without a detectable between-group difference in clinical or instrumented balance [40], which indicates that balance test results and fall outcomes should not be assumed to move together. The direction of effect should therefore be interpreted with caution, and future trials should conceal allocation and describe how, register their protocol and statistical analysis plan before enrolment, blind outcome assessors, analyse participants by intention to treat and minimise and report missing outcome data.

The Nintendo Wii and Xbox consoles used in most included trials are commercially available and relatively inexpensive, which makes them accessible to older adults at home or in community settings; more recent trials used stepping mats, force-platform games and consumer head-mounted displays [31,40,41,43,44,45]. Because the available trials did not show a consistent difference between VR and conventional balance training in either direction, such systems may offer an alternative format for delivering balance training, particularly for older adults who find conventional exercise monotonous [7]; the home-based, largely unsupervised stepping exergame that reduced falls [40] shows that this format can be delivered at scale, although adherence in that trial was below the prescribed dose. These possibilities remain to be confirmed in adequately powered trials that also assess adherence and cost.

The samples included in this review were ambulatory community-dwelling volunteers in whom frailty was not formally assessed in any trial, and we therefore avoid characterising them as “not frail” or “healthy”. A limited-responsiveness or ceiling-effect explanation for the modest between-group differences observed on the TUG and BBS is plausible in that reported baseline TUG values (approximately 7 to 12 s), which lie close to reference values for healthy older people [56], and BBS values (largely in the mid-50 s) leave little room for further improvement on those instruments in a community-dwelling sample. We did not, however, formally test for ceiling effects, and restricted instrument responsiveness is only one of several plausible explanations for the lack of between-group differences observed against active comparators; insufficient training dose, comparators of equivalent physiological potency and inadequate statistical power are equally consistent with the pattern of findings.

The pattern observed in this review, namely that VR was generally favoured over no training but showed no evidence of superiority over conventional balance training of similar dose, suggests that further small trials of generic off-the-shelf exergaming against no training are unlikely to be informative. More informative trials would compare VR with conventional balance training of matched dose, be powered for a prespecified clinically important difference or equivalence margin and report between-group estimates with confidence intervals. Because VR platforms record movement data by design, they could also allow difficulty progression and perturbation magnitude to be individualised based on measured postural control rather than age alone; whether such individualisation yields an additional effect is a question that these trials could test directly.

A related priority is to move beyond whether VR works to determining which modality works best at which dose. This cannot be resolved from the present literature because, with one exception, every included trial compared a single VR platform with a non-VR comparator; the exception compared two head-mounted displays in a small 3-week trial at high risk of bias [31]. The evidence network therefore lacks the connecting comparisons on which indirect estimation depends [16]. Network meta-analysis could rank competing modalities once such trials accumulate. Achieving this will require head-to-head trials of immersive versus non-immersive platforms, standardised reporting of dose, and agreement on a core outcome set for balance in community-dwelling older adults.

This review has several limitations. First, the PubMed search was updated to October 2026, but Embase, CINAHL and PEDro were not re-searched, and 48 reports identified by the update are awaiting classification; trials indexed only in those databases may have been missed, and the findings should be re-examined when the awaiting reports have been classified. Second, because pooling was judged inappropriate (Section 2.5), the magnitude and precision of any VR effect remain unquantified, and non-significant results from small trials cannot be interpreted as evidence of no effect. Third, the number of trials in each comparator-by-domain group was modest, and most samples were small. Fourth, only English-language reports were eligible, publication bias could not be assessed formally, and the reasons for the exclusions at the first full-text screening of the original search were not recorded at the report level. Fifth, the age criterion deviated from the registered protocol, although a sensitivity analysis restricted to trials meeting the registered criterion did not change the findings; and allocation concealment was not reported in 20 trials, which is reflected in the domain 1 judgements. Sixth, adherence, adverse events and follow-up were reported inconsistently, and only three trials assessed outcomes beyond the intervention period [41,48,53]; trial authors were not contacted for missing data. Seventh, the trials from the original searches overlap with those of our earlier meta-analysis of motor function [14]; data extraction and risk-of-bias assessment were performed afresh for this report, but the two reviews should not be regarded as independent sources of evidence. A future meta-analysis will become feasible only if trials converge on common comparators and a core outcome set and report between-group estimates with confidence intervals.

## 5. Conclusions

In community-dwelling older adults, VR-based balance training may improve balance compared with no training, but this finding rests mainly on trials with some concerns about the risk of bias or with a high risk of bias. There is no evidence that VR is superior to conventional balance training of comparable dose. However, because the comparative trials were small and rarely reported effect estimates with confidence intervals, neither superiority nor equivalence can be established, and the certainty of this evidence is low to very low. Home-based stepping exergames probably reduce falls compared with no training, on the basis of 1 large trial at low risk of bias; whether other VR formats reduce falls is unknown, so improvements in balance and balance confidence should still be regarded as surrogate outcomes rather than evidence of fall prevention. Training dose varied widely (15 to 60 min per session, 1 to 5 sessions per week, 3 weeks to 12 months), and because no trial compared doses, no conclusion about the required intensity can be drawn. VR can therefore be regarded as an alternative format for delivering balance training in this population rather than a proven superior intervention. Adequately powered trials against active comparators, matched for dose and supervision and designed to test superiority or equivalence against a prespecified clinically important difference, using a core outcome set and reporting between-group estimates with confidence intervals, are needed.

**Table 2:** General characteristics of included trials.

Study	Comparator Type	Groups (Intervention)	n Randomised/Analysed	Age (Years)	Sex (M/F)	Dose	Balance Outcomes	Between-Group Results (Balance)
Bateni [28]	Active; add-on arm	EG 1: Wii Fit + PT EG 2: Wii Fit CG: PT	6/6 6/6 6/6	68 ± 14 79 ± 13 72 ± 12	3/3 2/4 3/3	3 d × 4 wk	BBS Bubble test	BBS: PT and Wii Fit + PT > Wii Fit alone (*p* < 0.05) Bubble test: Wii Fit + PT > PT (*p* < 0.05)
Rendon et al. [49]	Inactive	EG: Nintendo Wii CG: No intervention	20/20 20/20	85.7 ± 4.3 83.3 ± 6.2	7/13 7/13	45–60 min × 3 d × 6 wk	8FTUG ABC	8FTUG and ABC: difference favoured EG (*p* < 0.05)
Franco et al. [27]	Active and inactive (3 arms)	EG: Wii Fit + home exercise CG 1: Matter of balance CG 2: No intervention	11/11 11/11 10/10	79.8 ± 4.7 77.9 ± 6.9 76.9 ± 6.3	2/9 3/8 2/8	30–45 min × 2 d × 3 wk	BBS Tinetti balance Wii balance board	No between-group difference in any balance outcome (*p* > 0.05)
Singh et al. [17,18]	Active	EG: Nintendo Wii CG: Conventional balance PT	18/18 18/18	61.12 ± 3.72 64.00 ± 5.88	0/18 0/18	40 min × 2 d × 6 wk	[17] PPA, ABC-6 [18] TUG, OPI, TST	[17] PPA and ABC-6: no between-group difference (*p* > 0.05) [18] TUG, OPI and TST: no between-group difference (*p* > 0.05)
Bieryla and Dold [29]	Inactive	EG: Wii Fit CG: Daily routine	12 randomised in total A: EG 4, CG 5	82.5 ± 1.6 80.5 ± 7.8	NR	30 min × 3 d × 3 wk	BBS FAB FR TUG	Between-group comparisons not reported for any balance outcome (within-group analyses only; not used as evidence of effect)
Jorgensen et al. [7]	Inactive	EG: Nintendo Wii CG: Insoles (daily use)	28/28 30/30	75.9 ± 5.7 73.7 ± 6.1	9/19 9/21	35 min × 2 d × 10 wk	TUG Static CoP (force plate) FES-I	TUG and FES-I: difference in change favoured EG (*p* < 0.05) Static postural balance (CoP): no between-group difference
Kim et al. [54]	Inactive	EG: Xbox 360 Kinect (unsupervised) CG: Daily routine	18/18 14/14	68.28 ± 3.74 66.21 ± 3.87	4/14 1/13	60 min × 3 d × 8 wk	Stepping tests (force plate)	Backward and crossover stepping with eyes closed: difference favoured EG (*p* < 0.05)
Schoene et al. [34]	Inactive	EG: Step-pad videogame (home, unsupervised) CG: Usual activities	18/15 19/17	78 ± 5 (completers, both arms)	NR	15–20 min × 2–3 d × 8 wk	CSRT PPA sway TUG Icon-FES	CSRT (primary) and PPA sway: difference favoured EG (*p* < 0.05) TUG and Icon-FES: no between-group difference
Eggenberger et al. [48]	Active	EG: Dance video game (StepMania) + strength and balance CG 1: Treadmill + verbal memory training + strength and balance CG 2: Treadmill + strength and balance	89 randomised in total A: 24; 22; 25	77.3 ± 6.3 78.5 ± 5.1 80.8 ± 4.7	10/14 6/16 9/16	60 min × 2 d × 26 wk	SPPB balance Falls	SPPB balance and fall frequency: no between-group difference (*p* > 0.05); 1-year follow-up
Gschwind et al. [35]	Inactive	EG: Kinect exergames + strength exercises (home, unsupervised) CG: Education booklet	78/NR 75/NR (136 re-assessed in total)	74.7 ± 6.7 74.7 ± 6.0	35/43 25/50	≥3 × 40 min balance + ≥3 × 15–20 min strength per wk × 16 wk	PPA sway Tandem stance Choice stepping SPPB TUG Icon-FES Falls	Sway, tandem stance, stepping reaction time, SPPB, TUG and Icon-FES: no between-group difference (*p* > 0.05) Falls: counted, between-group test not reported
Lee et al. [50]	Active	EG: Xbox 360 Kinect CG: Group-based exercise	26/26 28/28	68.77 ± 4.62 67.71 ± 4.31	0/26 0/28	60 min × 3 d × 8 wk	8FTUG	8FTUG: no between-group difference (*p* > 0.05)
Whyatt et al. [52]	Inactive	EG: Nintendo Wii (balance games) CG: Daily activity	40/40 42/42	77.18 ± 6.59 76.62 ± 7.28	5/35 20/22	30 min × 2 d × 5 wk	Force plate (static and dynamic)	Static (anterior, left, right) and dynamic balance: difference favoured EG (*p* < 0.05)
Kwok and Pua [47]	Active	EG: Nintendo Wii CG: Gym-based exercise	40/40 40/40	70.5 ± 6.7 69.8 ± 7.5	8/32 4/36	60 min × 1 d × 12 wk	MFES TUG NCW	MFES: difference favoured EG (*p* < 0.05) TUG and NCW: no between-group difference (*p* > 0.05)
Park and Yim [51]	Active (add-on)	EG: VR kayak + conventional exercise CG: Conventional exercise	36/36 36/36	72.94 ± 2.98 74.11 ± 2.88	3/33 1/35	VR 20 min + conventional 30 min × 2 d × 6 wk Conventional 30 min × 2 d × 6 wk	Force plate (standing and sitting balance)	Standing and sitting balance: difference in change favoured EG (*p* < 0.05); EG received a greater total dose
Ordnung et al. [36]	Inactive	EG: Xbox 360 Kinect CG: No training	15/14 15/15	69.8 ± 6.3 68.6 ± 4.7	7/7 7/8	60 min × 2 d × 6 wk	CoP sway (Wii Balance Board, eyes open and closed)	No between-group difference in any sway variable (*p* > 0.05)
Rodrigues et al. [55]	Inactive	EG: Xbox 360 Kinect (dance) CG: Daily activity	22/22 25/25	68 (67–70) 69 (67–73)	0/22 0/25	40 min × 3 d × 12 wk	TUG Falls	TUG and falls: no between-group difference (*p* > 0.05)
Adcock et al. [37]	Inactive	EG: Active@Home exergame (home-based, sensor-based) CG: Usual care	37 randomised in total A: 15; 16	77.0 ± 6.4 70.9 ± 5.0	5/10 10/6	30–40 min × 3 d × 16 wk	Extended SPPB balance Gait (single and dual task)	No between-group difference in any balance or gait outcome (*p* > 0.05)
Yu et al. [22]	Inactive	EG: Xbox 360 Kinect CG: Usual lifestyle	21/20 22/20	64.3 ± 4.8 63.8 ± 4.2	5/15 3/17	50 min (30 min gaming) × 3 d × 10 wk	8FTUG One-leg stance CoP (force plate)	8FTUG (*p* = 0.346) and CoP (*p* = 0.815; analysed in 17 and 14): no between-group difference
Barsasella et al. [23]	Inactive	EG: Various VR devices CG: No intervention	29/29 31/31	Range 60–94	4/25 10/21	15 min × 2 d × 6 wk	Unipedal stance	Unipedal stance: no between-group difference (*p* > 0.05)
Bagheri et al. [19]/Khanmohammadi et al. [20]	Active	EG: Nintendo Wii CG: Motor–cognitive dual-task training	[19] 31; 29 [20] 29; 28	71.83 ± 4.24 71.22 ± 5.82	[19] 10/21; 11/18 [20] 13/16; 13/15	60 min × 3 d × 5 wk (16 sessions)	[19] TUG, ABC, force plate [20] CoP during gait initiation	[19] TUG, ABC and force-plate measures: no between-group difference (*p* > 0.05) [20] ML displacement, ML velocity and AP velocity: difference favoured EG (*p* < 0.05)
Kim et al. [21]	Inactive	EG: VR bicycle riding programme CG: Daily activity	10/10 10/10	68.32 ± 6.32 64.70 ± 6.83	2/8 1/9	30 min × 2 d × 12 wk	TUG SPPB	TUG and SPPB: no between-group difference (change in TUG −0.23 vs. −0.07 s; SPPB +0.30 vs. −0.20 points)
Lee [38]	Active	EG: Treadmill gait training with VR scenery CG: Same treadmill training without VR	28/28 28/28	81.0 ± 6.9 79.5 ± 6.2	16/12 15/13	50 min × 5 d × 4 wk	BBS TUG One-leg stance FR	One-leg stance and TUG: difference in change favoured EG (*p* ≤ 0.002) BBS and FR: no between-group difference
Benitez-Lugo et al. [39]	Inactive	EG: Wii Fit + usual care CG: Usual care (centre workshops)	28/26 20/20	72.2 ± 4.8 73.0 ± 7.0	6/20 8/12	30 min × 2 d × 8 wk	BBS Tinetti TUG	BBS, Tinetti and TUG: difference in change favoured EG (one-sided *p* ≤ 0.014)
Zhao et al. [24]	Inactive	EG: Nintendo Switch exergames CG: Routine daily life	24/22 24/16	65.7 ± 3.8 (both arms)	14/24 (analysed, both arms)	50–55 min × 3 d × 12 wk	Force-plate sway (eyes open and closed)	Eyes-closed sway length, velocity and area: point estimates favoured EG; P not reported (F = 2.92–3.74)
Zak et al. [31]	Active	EG 1: HMD VR (smartphone headset) EG 2: Dual-task exercise + HMD VR EG 3: HMD VR (Oculus Rift S) CG: Otago programme	15/15 15/15 15/15 15/15	76.7 ± 1.5 78.1 ± 3.7 79.1 ± 3.6 76.7 ± 1.6	24/36 (all arms)	30 min × 3 d × 3 wk	TUG POMA Single-leg stance	TUG and POMA gait: post-intervention values favoured CG (*p* ≤ 0.04) Single-leg stance: favoured EG 2 and EG 3, with large baseline imbalance
Gerards et al. [46]	Inactive	EG: Perturbation-based VR treadmill training CG: Usual care	39/37 43/41	73 (IQR 8) 73 (IQR 10)	8/31 9/34	30 min × 1 d × 3 wk	Mini-BESTest FES-I	Mini-BESTest (*p* = 0.87) and FES-I (*p* = 0.85): no between-group difference
Carcelén-Fraile et al. [42]	Inactive	EG: Wii Fit (Balance Board) CG: Usual activities	38/37 38/36	69.9 ± 2.0 70.4 ± 1.5	12/25 9/27	30 min × 2 d × 12 wk	Tinetti (balance, gait, total)	Tinetti balance, gait and total: group × time interaction favoured EG (*p* ≤ 0.021)
Hashim et al. [53]	Active and inactive (3 arms)	EG: Nintendo Wii CG 1: Conventional balance PT CG 2: Usual care	42 randomised in total A: 16; 16; 11	72.5 ± 7.5 71.5 ± 5 74 ± 8	6/9 5/9 4/7	50 min × 3 d × 6 wk	FAB CoP (AP, ML)	FAB, AP and ML: EG and CG 1 each differed from CG 2 (*p* < 0.05); no difference between EG and CG 1; 3-week detraining follow-up
Sturnieks et al. [40]	Inactive (CG 2)	EG: Step-mat exergame (home, unsupervised) CG 1: Seated cognitive training (not used) CG 2: Education booklet	252/252 262/262 255/255 (falls; laboratory subsample 111; 118; 120)	72.6 ± 5.7 72.6 ± 5.5 72.5 ± 5.5	74/178 73/189 73/182	120 min per wk × 12 months	Falls Postural sway TUG SPPB CSRT Icon-FES	EG vs. CG 2: fall rate IRR 0.74 (95% CI 0.56–0.98); fallers RR 0.75 (0.61–0.92) Sway, TUG, SPPB and CSRT (6 mo): no between-group difference Icon-FES: favoured EG at 6 mo (MD −2.35, −4.40 to −0.31), not at 12 mo
Zak et al. [41]	Active	EG: Dual-task exercise + HMD VR CG: Otago programme	40/40 40/40	76.7 ± 2.2 76.8 ± 1.8	0/40 0/40	60 min × 3 d × 6 wk	BBS TUG Single-leg stance	TUG: post-intervention value favoured CG (*p* < 0.001) BBS and single-leg stance: no between-group difference; 3-week follow-up
Parmak et al. [43]	Active	EG: Immersive HMD VR (Meta Quest 2) CG: Home exercise brochure	22/22 22/22	71.1 ± 4.8 75.4 ± 9.2	4/18 4/18	35 min × 3 d × 8 wk	FAB FES-I	FAB (primary): ANCOVA favoured EG (*p* < 0.001) FES-I: no between-group difference (*p* = 0.106)
Huang et al. [44]	Active	EG: Game-based balance training (Biodex) CG: Home exercise videos	20/20 20/20	69.9 ± 4.3 70.8 ± 4.7	4/16 0/20	30 min × 1–2 d × 8 wk (16 sessions)	Sway indices (single and dual task) m-CTSIB TUG Single-leg stance FES-I	Single-task sway (all indices) and TUG: group × time interaction favoured EG (*p* ≤ 0.032) Dual-task sway: favoured EG with eyes open only Single-leg stance and FES-I: *p* < 0.05, direction not determinable from the reported data
Kim et al. [45]	Inactive	EG: HMD VR multimodal training CG: Usual activities	30/30 30/27	68.5 69.9	NR	30 min × 2 d × 8 wk	One-leg stance TUG	One-leg stance: difference in change favoured EG (*p* = 0.009) TUG: no between-group difference (values not reported)

Only balance outcomes and between-group results are shown; companion reports of the same trial are presented in a single row. Montero-Alía et al., whose study did not meet the eligibility criteria, has been removed (Supplementary Table S2). n randomised/analysed: number randomised/number analysed for the balance outcome. Rows for references [22,24,31,34,35,36,37,38,39,40,41,42,43,44,45]: trials identified in the updated PubMed search (8 October 2026). 8FTUG: 8-foot up-and-go; A: analysed; ABC: Activities-specific Balance Confidence scale; CI: confidence interval; CSRT: choice stepping reaction time; AP: anteroposterior; BBS: Berg Balance Scale; CG: control group; CoP: centre of pressure; d: days per week; EG: experimental group; FAB: Fullerton Advanced Balance Scale; FES-I: Falls Efficacy Scale–International; FR: Functional Reach test; HMD: head-mounted display; IRR: incidence rate ratio; m-CTSIB: modified Clinical Test of Sensory Interaction on Balance; MD: mean difference; IQR: interquartile range; MFES: modified Falls Efficacy Scale; ML: mediolateral; NCW: Narrow Corridor Walk Test; NR: not reported; OPI: Overall Performance Index; PPA: Physiological Profile Assessment; POMA: Performance-Oriented Mobility Assessment; PT: physical therapy; RR: risk ratio; SPPB: Short Physical Performance Battery; TST: Ten Step Test; TUG: Timed Up and Go Test; wk: weeks.

**Table 3:** Direction of the between-group effect for 1 prespecified outcome per trial and domain, with the overall RoB 2 judgement.

Comparison	Clinical Balance and Functional Mobility	Instrumented Postural Control	Balance Confidence/Fear of Falling	Falls	Overall Risk of Bias
VR vs. inactive comparator					
Rendon et al. [49]	8FTUG ▲ *	–	ABC ▲ *	–	Some concerns
Franco et al. [27] (vs. no intervention)	BBS ◄►	Wii balance board ◄►	–	–	Some concerns
Bieryla and Dold [29]	BBS †	–	–	–	High
Jorgensen et al. [7]	TUG ▲ *	Static CoP ◄►	FES-I ▲ *	–	Some concerns
Kim et al. [54]	–	Backward stepping, eyes closed ▲ *	–	–	High
Whyatt et al. [52]	–	Force-plate CoP ▲*	–	–	Some concerns
Rodrigues et al. [55]	TUG ◄►	–	–	Falls ◄►	High
Barsasella et al. [23]	Unipedal stance ◄►	–	–	–	Low
Kim et al. [21]	TUG ▲	–	–	–	High
Gerards et al. [46]	Mini-BESTest ◄►	–	FES-I ▲	–	Low
Hashim et al. [53] (vs. usual care)	FAB ▲ *	CoP (AP) ▲ *	–	–	Some concerns
Schoene et al. [34]	TUG ◄►	CSRT ▲ *	Icon-FES ◄►	–	Some concerns
Gschwind et al. [35]	TUG ▼	Sway area ▼	Icon-FES ▼	Falls ▲ †	Some concerns
Ordnung et al. [36]	–	CoP, eyes closed ◄►	–	–	Some concerns
Adcock et al. [37]	SPPB balance ◄►	–	–	–	High
Yu et al. [22]	8FTUG ▲	One-leg stance CoP ▲	–	–	Some concerns
Benitez-Lugo et al. [39]	BBS ▲ *	–	–	–	Some concerns
Zhao et al. [24]	–	Sway, eyes closed ▲	–	–	High
Sturnieks et al. [40] (vs. education)	TUG ▲	Postural sway ▼	Icon-FES ▲	Fall rate ▲ *	Low
Carcelén-Fraile et al. [42]	Tinetti balance ▲ *	–	–	–	Some concerns
Kim et al. [45]	One-leg stance ▲ *	–	–	–	Some concerns
VR vs. active comparator					
Bateni [28] (Wii Fit vs. PT; add-on arm: Wii Fit + PT vs. PT)	BBS ▼ *	Bubble test ◄► (add-on ▲ *)	–	–	Some concerns
Franco et al. [27] (vs. Matter of Balance)	BBS ◄►	Wii balance board ◄►	–	–	Some concerns
Singh et al. [17,18]	TUG ◄►	–	ABC-6 ◄►	–	Some concerns
Eggenberger et al. [48]	SPPB balance ◄►	–	–	Falls ◄►	High
Lee et al. [50]	8FTUG ◄►	–	–	–	Some concerns
Kwok and Pua [47]	TUG ◄►	–	MFES ▲ *	–	Some concerns
Park and Yim [51] (add-on)	–	Standing balance ▲ *	–	–	Some concerns
Bagheri et al. [19]/Khanmohammadi et al. [20]	TUG ◄►	CoP, gait initiation (ML displacement) ▲ *	ABC ◄►	–	High
Hashim et al. [53] (vs. conventional PT)	FAB ◄►	CoP (AP) ◄►	–	–	Some concerns
Lee [38] (vs. treadmill without VR)	BBS ▼	–	–	–	Some concerns
Zak et al. [31] (3 VR arms vs. Otago)	TUG ▼ *	–	–	–	High
Zak et al. [41] (vs. Otago)	BBS ▼	–	–	–	Some concerns
Parmak et al. [43]	FAB ▲ *	–	FES-I ▲	–	High
Huang et al. [44]	TUG ▲ *	Sway, dual task, eyes closed ▲	FES-I ◄► ‡	–	Some concerns

▲ Point estimate favours VR; ▼ point estimate favours the comparator; ◄► no clear between-group difference; * confidence interval or *p* value excludes no effect; † between-group test not reported; ‡ between-group difference reported (*p* < 0.05) but its direction could not be determined from the reported data; –, domain not assessed. Direction was recorded irrespective of statistical significance for the outcome selected with the hierarchy in Section 2.5. Trials [22,24,31,34,35,36,37,38,39,40,41,42,43,44,45] were identified in the updated PubMed search. Abbreviations as in Table 2.

**Table 4:** Summary of findings and certainty of evidence (GRADE) for each comparator-by-domain synthesis.

Comparator and Outcome Domain	Comparisons (Trials)	Participants *	Direction of Effect (Prespecified Outcome)	Certainty	Reasons for Downgrading	Interpretation
Inactive comparators						
Clinical balance and functional mobility	17 [7,21,22,23,27,29,34,35,37,39,40,42,45,46,49,53,55]	≈1000	Clear difference favouring VR in 6 [7,39,42,45,49,53], all with some concerns about the risk of bias; point estimates favouring VR without a clear difference in 3 [21,22,40]; no clear difference in 6; favouring the comparator in 1 [35]; not tested in 1 [29]. None of the 3 trials at low risk of bias showed a clear difference [23,40,46]	⊕ ⊕ ◯ ◯ Low	Risk of bias (−1); imprecision (−1)	VR may improve clinical balance compared with no training
Instrumented postural control	11 [7,22,24,27,34,35,36,40,52,53,54]	≈700	Clear difference favouring VR in 4 [34,52,53,54]; point estimate favouring VR in 2 [22,24]; no clear difference in 3; favouring the comparator in 2, including the largest trial [35,40]; variables and test conditions differed	⊕ ◯ ◯ ◯ Very low	Risk of bias (−1); inconsistency (−1); imprecision (−1)	The evidence is very uncertain
Balance confidence and fear of falling	6 [7,34,35,40,46,49]	≈850	Clear difference favouring VR in 2 [7,49]; point estimate favouring VR in 2 [40,46]; no clear difference in 1 [34]; favouring the comparator in 1 [35]	⊕ ◯ ◯ ◯ Very low	Risk of bias (−1); inconsistency (−1, different instruments); imprecision (−1)	The evidence is very uncertain
Falls	3 [35,40,55]	≈710	Fall rate and number of fallers clearly reduced in the largest trial, at low risk of bias (IRR 0.74, 95% CI 0.56–0.98) [40]; no clear difference in 1 [55]; counts favoured VR without a between-group test in 1 [35]	⊕ ⊕ ⊕ ◯ Moderate	Imprecision (−1; 1 adequately powered trial, upper confidence limit close to no effect)	Home-based stepping exergames probably reduce falls compared with no training; evidence for other VR formats is lacking
Active comparators						
Clinical balance and functional mobility	13 [17,18,19,27,28,31,38,41,43,44,47,48,50,53]	≈650	Clear difference favouring VR in 2 [43,44], 1 at high risk of bias; no clear difference in 7; point estimate favouring the comparator in 2 [38,41]; clear difference favouring the comparator in 2 [28,31]	⊕ ◯ ◯ ◯ Very low	Risk of bias (−1); inconsistency (−1, clear differences in both directions); imprecision (−1)	No consistent advantage of VR over conventional training of similar dose; equivalence cannot be established
Instrumented postural control	6 [19,20,27,28,44,51,53]	≈240	Clear difference favouring VR in selected variables in 3, 2 of them add-on comparisons [20,28,51]; point estimate favouring VR in 1 [44]; no clear difference in 2	⊕ ◯ ◯ ◯ Very low	Risk of bias (−1); inconsistency (−1); imprecision (−1)	The evidence is very uncertain
Balance confidence and fear of falling	5 [17,19,43,44,47]	≈260	Clear difference favouring VR in 1 [47]; point estimate favouring VR in 1 [43]; no clear difference in 3	⊕ ◯ ◯ ◯ Very low	Risk of bias (−1); inconsistency (−1); imprecision (−1)	The evidence is very uncertain
Falls	1 [48]	≈70	No clear difference	⊕ ◯ ◯ ◯ Very low	Risk of bias (−1, high); imprecision (−2)	The evidence is very uncertain; no evidence of fall prevention

* Approximate number of participants analysed, summed across comparisons from Table 2 (a control arm shared by 2 comparisons is counted once per comparison; where the number analysed was not reported, the number randomised was used). Certainty: ⊕ ⊕ ⊕ ◯ moderate, the true effect is probably close to the observed effect; ⊕ ⊕ ◯ ◯ low, the true effect may be substantially different from the observed effect; ⊕ ◯ ◯ ◯ very low, the true effect is likely to be substantially different. Indirectness and publication bias were not downgraded. A sensitivity analysis restricted to trials meeting the registered age criterion (excluding [17,18,21,22,23,24]) did not change any rating. CI: confidence interval; IRR: incidence rate r.

## Figures and Tables

**Table 1:** PICOS criteria for study eligibility.

Element	Inclusion	Exclusion
Participants	Older adults described as community-dwelling (living independently, not in residential or institutional care), ambulatory, with a reported mean age of ≥60 years in every study arm (registered criterion: ≥65 years; see Section 2.8)	Institutionalised samples; samples defined by a specific neurological or musculoskeletal diagnosis (e.g., stroke, Parkinson’s disease); samples recruited specifically as frail or pre-frail
Intervention	VR intervention as operationally defined in Section 2.6, delivered alone or in addition to other exercise	Interventions without real-time movement capture and concurrent visual feedback
Comparison	Inactive (no intervention, usual care, daily routine or activity, insole use) or active (conventional balance-oriented physiotherapy, gym- or group-based exercise, treadmill training, motor–cognitive dual-task training)	No control group
Outcomes	At least 1 balance outcome: clinical balance or functional mobility; instrumented postural control; balance confidence or fear of falling; falls	No balance outcome
Study design	Randomised controlled trials published in English	Quasi-randomised or non-randomised designs; conference abstracts without a full report

## Data Availability

The search strategies, the reports excluded in the updated search and the studies awaiting classification are provided in Supplementary Tables S1, S2 and S5; the risk-of-bias assessments are provided in Supplementary Table S4; the extracted data are presented in Table 2, Table 3 and Table 4.

## Supplementary Materials

The following supporting information can be downloaded at: https://www.mdpi.com/article/10.3390/jcm15218368/s1. Table S1: Search runs, strategies and numbers of records retrieved; Table S2: Reports excluded at eligibility assessment in the updated PubMed search, with reasons; Table S3: PRISMA 2020 checklist; Table S4: RoB 2 judgements with signalling-question responses and supporting information for each trial; Table S5: Studies awaiting classification identified in the updated PubMed search [57,58,59,60,61,62,63,64,65,66,67,68,69,70,71,72,73,74,75,76,77,78,79,80,81,82,83,84,85,86,87,88,89,90,91,92,93,94,95,96,97,98,99,100,101,102,103,104,105,106,107,108,109,110,111,112,113,114,115,116,117,118,119,120,121,122,123,124,125,126,127,128,129,130,131,132,133,134,135,136,137,138,139,140,141,142,143,144,145,146,147,148,149,150,151,152,153,154,155,156,157,158,159,160,161,162,163,164,165,166,167,168,169,170,171,172,173,174,175,176,177,178,179,180,181,182,183,184,185,186,187,188,189,190,191,192,193,194,195,196,197,198,199,200,201,202,203,204,205,206,207,208,209,210,211,212,213,214,215,216,217,218,219,220,221,222,223,224,225,226].

## References

[1] Shumway-Cook A., Woollacott M.H. (2023). Motor Control.

[2] Hicks C., Levinger P., Menant J.C., Lord S.R., Sachdev P.S., Brodaty H., Sturnieks D.L. (2020). Reduced Strength, Poor Balance and Concern about Falls Mediate the Relationship between Knee Pain and Fall Risk in Older People. BMC Geriatr..

[3] Guirguis-Blake J.M., Perdue L.A., Coppola E.L., Bean S.I. (2024). Interventions to Prevent Falls in Older Adults: Updated Evidence Report and Systematic Review for the US Preventive Services Task Force. JAMA.

[4] Sherrington C., Fairhall N.J., Wallbank G.K., Tiedemann A., Michaleff Z.A., Howard K., Clemson L., Hopewell S., Lamb S.E. (2019). Exercise for Preventing Falls in Older People Living in the Community. Cochrane Database Syst. Rev..

[5] Moreno-Segura N., Igual-Camacho C., Ballester-Gil Y., Blasco-Igual M.C., Blasco J.M. (2018). The Effects of the Pilates Training Method on Balance and Falls of Older Adults: A Systematic Review and Meta-analysis of Randomized Controlled Trials. J. Aging Phys. Act..

[6] Raj N.B., Lee W.Z., Eswaramoorthi V., Razali H., Othman N.Y.H., Rao U.M. (2020). Exercise Programmes for Reducing the Risk of Falls in Elderly: A Review. Res. J. Pharm. Technol..

[7] Jorgensen M.G., Laessoe U., Hendriksen C., Nielsen O.B.F., Aagaard P. (2013). Efficacy of Nintendo Wii Training on Mechanical Leg Muscle Function and Postural Balance in Community-Dwelling Older Adults: A Randomized Controlled Trial. J. Gerontol. Ser. A Biol. Sci. Med. Sci..

[8] Seah B., Kowitlawakul Y., Jiang Y., Ang E., Chokkanathan S., Wang W. (2019). A Review on Healthy Ageing Interventions Addressing Physical, Mental and Social Health of Independent Community-Dwelling Older Adults. Geriatr. Nurs..

[9] Warnier N., Lambregts S., Port I.V.D. (2020). Effect of Virtual Reality Therapy on Balance and Walking in Children with Cerebral Palsy: A Systematic Review. Dev. Neurorehabilit..

[10] Wu J., Zeng A., Chen Z., Wei Y., Huang K., Chen J., Ren Z. (2021). Effects of Virtual Reality Training on Upper Limb Function and Balance in Stroke Patients: Systematic Review and Meta-meta-Analysis. J. Med. Internet Res..

[11] Lei C., Sunzi K., Dai F., Liu X., Wang Y., Zhang B., He L., Ju M. (2019). Effects of Virtual Reality Rehabilitation Training on Gait and Balance in Patients with Parkinson’s Disease: A Systematic Review. PLoS ONE.

[12] Truijen S., Abdullahi A., Bijsterbosch D., van Zoest E., Conijn M., Wang Y., Struyf N., Saeys W. (2022). Effect of Home-Based Virtual Reality Training and Telerehabilitation on Balance in Individuals with Parkinson Disease, Multiple Sclerosis, and Stroke: A Systematic Review and Meta-analysis. Neurol. Sci..

[13] Neri S.G., Cardoso J.R., Cruz L., Lima R.M., De Oliveira R.J., Iversen M.D., Carregaro R.L. (2017). Do virtual reality games improve mobility skills and balance measurements in community-dwelling older adults? Systematic review and meta-analysis. Clin. Rehabil..

[14] Han Y.-G., Park S.-W. (2025). Effects of Virtual Reality Intervention on Motor Function in Community-Dwelling Older Adults: A Systematic Review and Meta-Analysis. Medicine.

[15] Page M.J., McKenzie J.E., Bossuyt P.M., Boutron I., Hoffmann T.C., Mulrow C.D., Shamseer L., Tetzlaff J.M., Akl E.A., Brennan S.E. (2021). The PRISMA 2020 Statement: An Updated Guideline for Reporting Systematic Reviews. BMJ.

[16] Higgins J.P.T., Thomas J., Chandler J., Cumpston M., Li T., Page M.J., Welch V.A. (2024). Cochrane Handbook for Systematic Reviews of Interventions, version 6.5.

[17] Singh D.K.A., Rajaratnam B.S., Palaniswamy V., Pearson H., Raman V.P., Bong P.S. (2012). Participating in a Virtual Reality Balance Exercise Program Can Reduce Risk and Fear of Falls. Maturitas.

[18] Singh D.K.A., Rajaratnam B.S., Palaniswamy V., Raman V.P., Bong P.S., Pearson H. (2013). Effects of Balance-Focused Interactive Games Compared to Therapeutic Balance Classes for Older Women. Climacteric.

[19] Bagheri H., Khanmohammadi R., Olyaei G., Talebian S., Reza Hadian M.R., Najafi M. (2021). Video Game and Motor-Cognitive Dual-Task Training Could Be Suitable Treatments to Improve Dual-Task Interference in Older Adults. Neurosci. Lett..

[20] Khanmohammadi R., Olyaei G., Talebian S., Hadian M.R., Bagheri H., Aliabadi S. (2022). The Effect of Video Game-Based Training on Postural Control during Gait Initiation in Community-Dwelling Older Adults: A Randomized Controlled Trial. Disabil. Rehabil..

[21] Kim D.-R., Song S., Kim G.-M., Chang J.H., Tak Y.J., Huh U., Cho J.S., Liao Y., Han K.-S., Ko M.-H. (2021). Effects of ICT-Based Multicomponent Program on Body Composition and Cognitive Function in Older Adults: A Randomized Controlled Clinical Study. Clin. Interv. Aging.

[22] Yu T.-C., Chiang C.-H., Wu P.-T., Wu W.-L., Chu I.-H. (2020). Effects of Exergames on Physical Fitness in Middle-Aged and Older Adults in Taiwan. Int. J. Environ. Res. Public Health.

[23] Barsasella D., Liu M.F., Malwade S., Galvin C.J., Dhar E., Chang C.-C., Li Y.J., Syed-Abdul S. (2021). Effects of Virtual Reality Sessions on the Quality of Life, Happiness, and Functional Fitness among the Older People: A Randomized Controlled Trial from Taiwan. Comput. Methods Programs Biomed..

[24] Zhao C., Zhao C., Li Y., Zhao M., Wang L., Guo J., Zhang L., Sun Y., Ye X., Zhu W. (2022). The Effects of Active Video Game Exercise Based on Self-Determination Theory on Physical Fitness and Cognitive Function in Older Adults. J. Clin. Med..

[25] Sterne J.A.C., Savović J., Page M.J., Elbers R.G., Blencowe N.S., Boutron I., Cates C.J., Cheng H.Y., Corbett M.S., Eldridge S.M. (2019). RoB 2: A revised tool for assessing risk of bias in randomised trials. BMJ.

[26] Campbell M., McKenzie J.E., Sowden A., Katikireddi S.V., Brennan S.E., Ellis S., Hartmann-Boyce J., Ryan R., Shepperd S., Thomas J. (2020). Synthesis without Meta-analysis (SWiM) in Systematic Reviews: Reporting Guideline. BMJ.

[27] Franco J.R., Jacobs K., Inzerillo C., Kluzik J. (2012). The Effect of the Nintendo Wii Fit and Exercise in Improving Balance and Quality of Life in Community Dwelling Elders. Technol. Health Care.

[28] Bateni H. (2012). Changes in Balance in Older Adults Based on Use of Physical Therapy vs the Wii Fit Gaming System: A Preliminary Study. Physiotherapy.

[29] Bieryla K.A., Dold N.M. (2013). Feasibility of Wii Fit Training to Improve Clinical Measures of Balance in Older Adults. Clin. Interv. Aging.

[30] Thomson H.J., Thomas S. (2013). The Effect Direction Plot: Visual Display of Non-Standardised Effects across Multiple Outcome Domains. Res. Synth. Methods.

[31] Zak M., Sikorski T., Krupnik S., Wasik M., Grzanka K., Courteix D., Dutheil F., Brola W. (2022). Physiotherapy Programmes Aided by VR Solutions Applied to the Seniors Affected by Functional Capacity Impairment: Randomised Controlled Trial. Int. J. Environ. Res. Public Health.

[32] Guyatt G.H., Oxman A.D., Vist G.E., Kunz R., Falck-Ytter Y., Alonso-Coello P., Schünemann H.J. (2008). GRADE: An Emerging Consensus on Rating Quality of Evidence and Strength of Recommendations. BMJ.

[33] Murad M.H., Mustafa R.A., Schünemann H.J., Sultan S., Santesso N. (2017). Rating the Certainty in Evidence in the Absence of a Single Estimate of Effect. Evid. Based Med..

[34] Schoene D., Lord S.R., Delbaere K., Severino C., Davies T.A., Smith S.T. (2013). A randomized controlled pilot study of home-based step training in older people using videogame technology. PLoS ONE.

[35] Gschwind Y.J., Eichberg S., Ejupi A., de Rosario H., Kroll M., Marston H.R., Drobics M., Annegarn J., Wieching R., Lord S.R. (2015). ICT-based system to predict and prevent falls (iStoppFalls): Results from an international multicenter randomized controlled trial. Eur. Rev. Aging Phys. Act..

[36] Ordnung M., Hoff M., Kaminski E., Villringer A., Ragert P. (2017). No Overt Effects of a 6-Week Exergame Training on Sensorimotor and Cognitive Function in Older Adults. A Preliminary Investigation. Front. Hum. Neurosci..

[37] Adcock M., Fankhauser M., Post J., Lutz K., Zizlsperger L., Luft A.R., Guimarães V., Schättin A., de Bruin E.D. (2019). Effects of an In-home Multicomponent Exergame Training on Physical Functions, Cognition, and Brain Volume of Older Adults: A Randomized Controlled Trial. Front. Med..

[38] Lee K. (2021). Virtual Reality Gait Training to Promote Balance and Gait Among Older People: A Randomized Clinical Trial. Geriatrics.

[39] Benitez-Lugo M.-L., Suárez-Serrano C., Galvao-Carmona A., Vazquez-Marrufo M., Chamorro-Moriana G. (2022). Effectiveness of feedback-based technology on physical and cognitive abilities in the elderly. Front. Aging Neurosci..

[40] Sturnieks D.L., Hicks C., Smith N., Ratanapongleka M., Menant J., Turner J., Lo J., Chaplin C., Garcia J., Valenzuela M.J. (2024). Exergame and cognitive training for preventing falls in community-dwelling older people: A randomized controlled trial. Nat. Med..

[41] Zak M., Sikorski T., Michalska A., Sztandera P., Szczepanowska-Wolowiec B., Brola W., Courteix D., Dutheil F. (2024). The Effects of Physiotherapy Programmes, Aided by Virtual Reality Solutions, on Balance in Older Women: A Randomised Controlled Trial. J. Clin. Med..

[42] Carcelén-Fraile M.D.C., Aibar-Almazán A., Hita-Contreras F., Sánchez-Alcalá M., Parra-Díaz A.B., Infante-Guedes A., Castellote-Caballero Y. (2024). Using the Nintendo™ Wii to Improve Physical Function and Reduce the Risk of Falls in Older Adults: A Randomized Controlled Clinical Trial. Sensors.

[43] Parmak D., Angın E., Iyigun G. (2025). Effects of Immersive Virtual Reality on Physical Function, Fall-Related Outcomes, Fatigue, and Quality of Life in Older Adults: A Randomized Controlled Trial. Healthcare.

[44] Huang C.-J., Chen H.-C., Oknina L., Huang S.-W., Lin L.-F. (2026). Effects of game-based virtual reality balance training on postural stability and mobility in community-dwelling older adults: An exploratory randomized controlled trial. BMC Geriatr..

[45] Kim D.-R., Shin M.-J., Lee E.-L., Lai T.-F., Yang Y.-A., Park J.-H. (2026). Effects of a virtual reality-based multimodal training program on cognitive and physical functions in community-dwelling adults aged 60 years and older: A randomized pre-post controlled study. BMC Geriatr..

[46] Gerards M., Marcellis R., Senden R., Poeze M., de Bie R., Meijer K., Lenssen A. (2023). The Effect of Perturbation-Based Balance Training on Balance Control and Fear of Falling in Older Adults: A Single-Blind Randomised Controlled Trial. BMC Geriatr..

[47] Kwok B.C., Pua Y.H. (2016). Effects of WiiActive Exercises on Fear of Falling and Functional Outcomes in Community-Dwelling Older Adults: A Randomised Control Trial. Age Ageing.

[48] Eggenberger P., Theill N., Holenstein S., Schumacher V., de Bruin E.D. (2015). Multicomponent Physical Exercise with Simultaneous Cognitive Training to Enhance Dual-Task Walking of Older Adults: A Secondary Analysis of a 6-Month Randomized Controlled Trial with 1-Year Follow-Up. Clin. Interv. Aging.

[49] Rendon A.A., Lohman E.B., Thorpe D., Johnson E.G., Medina E., Bradley B. (2012). The Effect of Virtual Reality Gaming on Dynamic Balance in Older Adults. Age Ageing.

[50] Lee M., Son J., Kim J., Yoon B. (2015). Individualized Feedback-Based Virtual Reality Exercise Improves Older Women’s Self-Perceived Health: A Randomized Controlled Trial. Arch. Gerontol. Geriatr..

[51] Park J., Yim J. (2016). A New Approach to Improve Cognition, Muscle Strength, and Postural Balance in Community-Dwelling Elderly with a 3-D Virtual Reality Kayak Program. Tohoku J. Exp. Med..

[52] Whyatt C., Merriman N.A., Young W.R., Newell F.N., Craig C. (2015). A Wii Bit of Fun: A Novel Platform to Deliver Effective Balance Training to Older Adults. Games Health J..

[53] Hashim J., Campelo A.M., Doyle-Baker P.K., Cossich V.R.A., Katz L. (2024). Comparison between Six-Week Exergaming, Conventional Balance and No Exercise Training Program on Older Adults’ Balance and Gait Speed. Int. J. Comput. Sci. Sport.

[54] Kim J., Son J., Ko N., Yoon B. (2013). Unsupervised Virtual Reality-Based Exercise Program Improves Hip Muscle Strength and Balance Control in Older Adults: A Pilot Study. Arch. Phys. Med. Rehabil..

[55] Rodrigues E.V., Gallo L.H., Guimarães A.T.B., Melo Filho J., Luna B.C., Gomes A.R.S. (2018). Effects of Dance Exergaming on Depressive Symptoms, Fear of Falling, and Musculoskeletal Function in Fallers and Nonfallers Community-Dwelling Older Women. Rejuvenation Res..

[56] Long J., Cai T., Huang X., Zhou Y., Kuang J., Wu L. (2020). Reference Value for the TUGT in Healthy Older People: A Systematic Review and Meta-analysis. Geriatr. Nurs..

[57] Alhasan H.S., Wheeler P.C., Fong D.T.P. (2021). Application of Interactive Video Games as Rehabilitation Tools to Improve Postural Control and Risk of Falls in Prefrail Older Adults. Cyborg Bionic Syst..

[58] Alhasan H.S., Alshehri M.A. (2025). Gaming Against Frailty: Effects of Virtual Reality-Based Training on Postural Control, Mobility, and Fear of Falling Among Frail Older Adults. J. Clin. Med..

[59] Anguera J.A., Volponi J.J., Simon A.J., Gallen C.L., Rolle C.E., Anguera-Singla R., Pitsch E.A., Thompson C.J., Gazzaley A. (2022). Integrated cognitive and physical fitness training enhances attention abilities in older adults. npj Aging.

[60] Areiza R., Osorio F.V., Montoya-Alvarez L., Rodriguez-Sepulveda E., Perez L., Pérez A.S., Martínez S.C.E. (2026). Physical Capacity Changes Following a Virtual Reality-Assisted Exercise Program in Institutionalized Older Adults: A Pilot Intervention Study. Healthcare.

[61] Arnold N., Wilson O., Thompson L. (2024). Virtual Reality Training Affects Center of Pressure (COP)-Based Balance Parameters in Older Individuals. Appl. Sci..

[62] Bakker J., Donath L., Rein R. (2020). Balance training monitoring and individual response during unstable vs. stable balance Exergaming in elderly adults: Findings from a randomized controlled trial. Exp. Gerontol..

[63] Batcir S., Livne Y., Lev Lehman R., Edelman S., Schiller L., Lubovsky O., Shani G., Shapiro A., Melzer I. (2021). Development and piloting of a perturbation stationary bicycle robotic system that provides unexpected lateral perturbations during bicycling (the PerStBiRo system). BMC Geriatr..

[64] Begde A., Alqurafi A., Pain M.T.G., Blenkinsop G., Wilcockson T.D.W., Hogervorst E. (2023). The Effectiveness of Home-Based Exergames Training on Cognition and Balance in Older Adults: A Comparative Quasi-Randomized Study of Two Exergame Interventions. Innov. Aging.

[65] Belleville S., Cuesta M., Bieler-Aeschlimann M., Giacomino K., Widmer A., Mittaz Hager A.G., Perez-Marcos D., Cardin S., Boller B., Bier N. (2023). Pre-frail older adults show improved cognition with StayFitLonger computerized home-based training: A randomized controlled trial. Geroscience.

[66] Blanco V., Otero P., Cotardo T., Bouza Q., Torres Á.J., Vázquez F.L. (2025). Efficacy of a video game for promoting active and healthy aging (Initial Version): A randomized controlled trial. BMC Public Health.

[67] Bourrelier J., Fautrelle L., Haratyk E., Manckoundia P., Mérienne F., Mourey F., Kubicki A. (2021). Enhancement of Anticipatory Postural Adjustments by Virtual Reality in Older Adults with Cognitive and Motor Deficits: A Randomised Trial. Geriatrics.

[68] Cai H., Li G., Jiang S., Yin H., Liu P., Chen L. (2019). Effect of Low-Intensity, Kinect™-Based Kaimai-Style Qigong Exercise in Older Adults With Type 2 Diabetes. J. Gerontol. Nurs..

[69] Campo-Prieto P., Cancela-Carral J.M., Alsina-Rey B., Rodríguez-Fuentes G. (2022). Immersive Virtual Reality as a Novel Physical Therapy Approach for Nonagenarians: Usability and Effects on Balance Outcomes of a Game-Based Exercise Program. J. Clin. Med..

[70] Campo-Prieto P., Cancela-Carral J.M., Rodríguez-Fuentes G. (2022). Feasibility and Effects of an Immersive Virtual Reality Exergame Program on Physical Functions in Institutionalized Older Adults: A Randomized Clinical Trial. Sensors.

[71] Chen P.Y., Wei S.H., Hsieh W.L., Cheen J.R., Chen L.K., Kao C.L. (2012). Lower limb power rehabilitation (LLPR) using interactive video game for improvement of balance function in older people. Arch. Gerontol. Geriatr..

[72] Chow Y.E., Yoong S.Q., Oide K., Chen C., Lee P.Y., Chua M.C.H., Jiang Y. (2025). Older adults’ perceptions and experiences of the SinDance exergame: A process evaluation. Geriatr. Nurs..

[73] Cicek A., Ozdincler A.R., Tarakci E. (2020). Interactive video game-based approaches improve mobility and mood in older adults: A nonrandomized, controlled trial. J. Bodyw. Mov. Ther..

[74] Ciemer C., Schott N., Klotzbier T.J., Ghellal S. (2025). Effectiveness, enjoyment, and meaningfulness of a virtual reality gait-based fall prevention exergame in community-dwelling healthy older adults: An interdisciplinary pilot study. Front. Psychol..

[75] Corrêa F.I., Kunitake A.I., Segheto W., Duarte de Oliveira M., Fregni F., Ferrari Corrêa J.C. (2024). The effect of transcranial direct current stimulation associated with video game training on the postural balance of older women in the community: A blind, randomized, clinical trial. Physiother. Res. Int..

[76] Damasceno S.O., Pizzo Junior E., Cavina A.P.S., Dos Santos D.S.P., da Cruz M.E.L., Pimenta G.S.O.M., Vanderlei F.M. (2026). Responses in Posttherapy Recovery-Based Virtual Reality with Blood Flow Restriction in Older Women: A Randomized Crossover Clinical Trial. J. Aging Phys. Act..

[77] Daniel K. (2012). Wii-hab for pre-frail older adults. Rehabil. Nurs..

[78] Del Din S., Galna B., Lord S., Nieuwboer A., Bekkers E.M.J., Pelosin E., Avanzino L., Bloem B.R., Olde Rikkert M.G.M., Nieuwhof F. (2020). Falls Risk in Relation to Activity Exposure in High-Risk Older Adults. J. Gerontol. A. Biol. Sci. Med. Sci..

[79] Delbroek T., Vermeylen W., Spildooren J. (2017). The effect of cognitive-motor dual task training with the biorescue force platform on cognition, balance and dual task performance in institutionalized older adults: A randomized controlled trial. J. Phys. Ther. Sci..

[80] Ditchburn J.L., van Schaik P., Dixon J., MacSween A., Martin D. (2020). The effects of exergaming on pain, postural control, technology acceptance and flow experience in older people with chronic musculoskeletal pain: A randomised controlled trial. BMC Sports Sci. Med. Rehabil..

[81] Dockx K., Alcock L., Bekkers E., Ginis P., Reelick M., Pelosin E., Lagravinese G., Hausdorff J.M., Mirelman A., Rochester L. (2017). Fall-Prone Older People’s Attitudes towards the Use of Virtual Reality Technology for Fall Prevention. Gerontology.

[82] Eggenberger P., Schumacher V., Angst M., Theill N., de Bruin E.D. (2015). Does multicomponent physical exercise with simultaneous cognitive training boost cognitive performance in older adults? A 6-month randomized controlled trial with a 1-year follow-up. Clin. Interv. Aging.

[83] Eggenberger P., Wolf M., Schumann M., de Bruin E.D. (2016). Exergame and Balance Training Modulate Prefrontal Brain Activity during Walking and Enhance Executive Function in Older Adults. Front. Aging Neurosci..

[84] Eggenberger P., Annaheim S., Kündig K.A., Rossi R.M., Münzer T., de Bruin E.D. (2020). Heart Rate Variability Mainly Relates to Cognitive Executive Functions and Improves Through Exergame Training in Older Adults: A Secondary Analysis of a 6-Month Randomized Controlled Trial. Front. Aging Neurosci..

[85] Fakhro M.A., Hadchiti R., Awad B. (2020). Effects of Nintendo Wii fit game training on balance among Lebanese older adults. Aging Clin. Exp. Res..

[86] Filho J.M., Biesek S., Vojciechowski A.S., Tormes G.A., Gomes A.R.S. (2022). Effects of multidomain interventions on skeletal muscle architecture and function in pre-frail older women: The WiiProtein study. Geriatr. Nurs..

[87] Fu A.S., Gao K.L., Tung A.K., Tsang W.W., Kwan M.M. (2015). Effectiveness of Exergaming Training in Reducing Risk and Incidence of Falls in Frail Older Adults with a History of Falls. Arch. Phys. Med. Rehabil..

[88] Fung V., Ho A., Shaffer J., Chung E., Gomez M. (2012). Use of Nintendo Wii Fit™ in the rehabilitation of outpatients following total knee replacement: A preliminary randomised controlled trial. Physiotherapy.

[89] Gallo L.H., Rodrigues E.V., Filho J.M., da Silva J.B., Harris-Love M.O., Gomes A.R.S. (2019). Effects of virtual dance exercise on skeletal muscle architecture and function of community dwelling older women. J. Musculoskelet. Neuronal Interact..

[90] Gatica-Rojas V., Cartes-Velásquez R., Silva-Llanos J., Arenas-Leiva C., De Vitis V., Posella S., Cofré Lizama L.E. (2025). Telerehabilitation and Face-to-Face Exergame Delivery Modalities to Improve Postural Control in Older Adults: A Randomised Controlled Trial. Med. Sci..

[91] Ghasemian M., Tajpour M., Mollanuri P., Zamanpour E., Moradi H. (2024). Computerized cognitive games versus cognitive exergame: The comparison of motor and cognitive functions enhancement in the elderly. BMC Psychol..

[92] Gomes G.C.V., Simões M.D.S., Lin S.M., Bacha J.M.R., Viveiro L.A.P., Varise E.M., Carvas Junior N., Lange B., Jacob Filho W., Pompeu J.E. (2018). Feasibility, safety, acceptability, and functional outcomes of playing Nintendo Wii Fit Plus(TM) for frail older adults: A randomized feasibility clinical trial. Maturitas.

[93] Gouveia É.R., Smailagic A., Ihle A., Marques A., Gouveia B.R., Cameirão M., Sousa H., Kliegel M., Siewiorek D. (2021). The Efficacy of a Multicomponent Functional Fitness Program Based on Exergaming on Cognitive Functioning of Healthy Older Adults: A Randomized Controlled Trial. J. Aging Phys. Act..

[94] Gschwind Y.J., Schoene D., Lord S.R., Ejupi A., Valenzuela T., Aal K., Woodbury A., Delbaere K. (2015). The effect of sensor-based exercise at home on functional performance associated with fall risk in older people—A comparison of two exergame interventions. Eur. Rev. Aging Phys. Act..

[95] Hagedoorn L., Ruiz-Rodríguez A., Leijen I., van Asseldonk E., Weerdesteyn V. (2025). Action observation with motor simulation of reactive stepping: A randomized study in older adults with a history of falls. Exp. Gerontol..

[96] Hassett L., van den Berg M., Lindley R.I., Crotty M., McCluskey A., van der Ploeg H.P., Smith S.T., Schurr K., Howard K., Hackett M.L. (2020). Digitally enabled aged care and neurological rehabilitation to enhance outcomes with Activity and MObility UsiNg Technology (AMOUNT) in Australia: A randomised controlled trial. PLoS Med..

[97] Hauer K., Litz E., Günther-Lange M., Ball C., de Bruin E.D., Werner C. (2020). Effectiveness and sustainability of a motor-cognitive stepping exergame training on stepping performance in older adults: A randomized controlled trial. Eur. Rev. Aging Phys. Act..

[98] He Y., Lee J., Kim J., Brodie M.A., Mitri G., van Schooten K.S., Lovell N.H., Lord S.R., Okubo Y. (2025). Virtual obstacle-avoidance training using daily-life obstacles with physical feedback in older people: A cross-over trial. Arch. Gerontol. Geriatr..

[99] Huang S.F., Fan C.W., Nisrina D.K., Lin T.W., Huang W.C. (2025). A Novel Gamified Exercise Program Incorporating Stampede Training for Enhancing Functional Fitness, Physical Activity Levels, and Quality of Life in Community-Dwelling Older Adults: Randomized Parallel Exploratory Trial. JMIR Serious Games.

[100] Hwang N.K., Choi J.B., Choi D.K., Park J.M., Hong C.W., Park J.S., Yoon T.H. (2021). Effects of Semi-Immersive Virtual Reality-Based Cognitive Training Combined with Locomotor Activity on Cognitive Function and Gait Ability in Community-Dwelling Older Adults. Healthcare.

[101] Jahouh M., González-Bernal J.J., González-Santos J., Fernández-Lázaro D., Soto-Cámara R., Mielgo-Ayuso J. (2021). Impact of an Intervention with Wii Video Games on the Autonomy of Activities of Daily Living and Psychological-Cognitive Components in the Institutionalized Elderly. Int. J. Environ. Res. Public Health.

[102] Janhunen M., Katajapuu N., Paloneva J., Pamilo K., Oksanen A., Keemu H., Karvonen M., Luimula M., Korpelainen R., Jämsä T. (2023). Effects of a home-based, exergaming intervention on physical function and pain after total knee replacement in older adults: A randomised controlled trial. BMJ Open Sport Exerc. Med..

[103] Janssen S., Tange H., Arends R. (2013). A Preliminary Study on the Effectiveness of Exergame Nintendo “Wii Fit Plus” on the Balance of Nursing Home Residents. Games Health J..

[104] Jeon M.J., Jeon H.S., Yi C.H., Kwon O.Y., You S.H., Park J.H. (2021). Block and Random Practice: A Wii Fit Dynamic Balance Training in Older Adults. Res. Q. Exerc. Sport.

[105] Kannan L., Sahu U., Subramaniam S., Mehta N., Kaur T., Hughes S., Bhatt T. (2024). Gaming-Based Tele-Exercise Program to Improve Physical Function in Frail Older Adults: Feasibility Randomized Controlled Trial. J. Med. Internet Res..

[106] Kayama H., Okamoto K., Nishiguchi S., Yukutake T., Tanigawa T., Nagai K., Yamada M., Aoyama T. (2013). Efficacy of an Exercise Game Based on Kinect in Improving Physical Performances of Fall Risk Factors in Community-Dwelling Older Adults. Games Health J..

[107] Kayama H., Okamoto K., Nishiguchi S., Yamada M., Kuroda T., Aoyama T. (2014). Effect of a Kinect-based exercise game on improving executive cognitive performance in community-dwelling elderly: Case control study. J. Med. Internet Res..

[108] Kim S.H., Cho S.H. (2022). Benefits of Virtual Reality Program and Motor Imagery Training on Balance and Fall Efficacy in Isolated Older Adults: A Randomized Controlled Trial. Medicina.

[109] Kim T., Xiong S. (2022). Effectiveness and Usability of a Novel Kinect-Based Tailored Interactive Fall Intervention System for Fall Prevention in Older People: A Preliminary Study. Front. Public Health.

[110] Konstantinidis E.I., Billis A.S., Mouzakidis C.A., Zilidou V.I., Antoniou P.E., Bamidis P.D. (2016). Design, Implementation, and Wide Pilot Deployment of FitForAll: An Easy to use Exergaming Platform Improving Physical Fitness and Life Quality of Senior Citizens. IEEE J. Biomed. Health Inform..

[111] Kubica J., Szymura J., Domagalik A., Golda S., Wiecek M., Fafrowicz M., Marek T., Pera J. (2019). Systematic Balance Exercises Influence Cortical Activation and Serum BDNF Levels in Older Adults. J. Clin. Med..

[112] Kubicki A., Petrement G., Bonnetblanc F., Ballay Y., Mourey F. (2012). Practice-related improvements in postural control during rapid arm movement in older adults: A preliminary study. J. Gerontol. A. Biol. Sci. Med. Sci..

[113] Kubicki A., Bonnetblanc F., Petrement G., Mourey F. (2014). Motor-prediction improvements after virtual rehabilitation in geriatrics: Frail patients reveal different learning curves for movement and postural control. Neurophysiol. Clin..

[114] Kwan R.Y.C., Liu J.Y.W., Fong K.N.K., Qin J., Leung P.K., Sin O.S.K., Hon P.Y., Suen L.W., Tse M.K., Lai C.K. (2021). Feasibility and Effects of Virtual Reality Motor-Cognitive Training in Community-Dwelling Older People With Cognitive Frailty: Pilot Randomized Controlled Trial. JMIR Serious Games.

[115] Kwan R.Y.C., Liu J., Sin O.S.K., Fong K.N.K., Qin J., Wong J.C.Y., Lai C. (2024). Effects of Virtual Reality Motor-Cognitive Training for Older People With Cognitive Frailty: Multicentered Randomized Controlled Trial. J. Med. Internet Res..

[116] Köyağasıoğlu O., Özgürbüz C., Bediz C.Ş., Güdücü Ç., Aydınoğlu R., Akşit T. (2022). The Effects of Virtual Reality Nonphysical Mental Training on Balance Skills and Functional Near-Infrared Spectroscopy Activity in Healthy Adults. J. Sport Rehabil..

[117] Lee S., Shin S. (2013). Effectiveness of virtual reality using video gaming technology in elderly adults with diabetes mellitus. Diabetes Technol. Ther..

[118] Leung K.M., Shi Y. (2025). Effects of Competitive Exergaming-Based Esports on Older Adults in Hong Kong: Nonrandomized Controlled Pilot Study. JMIR Serious Games.

[119] Levy F., Leboucher P., Rautureau G., Komano O., Millet B., Jouvent R. (2016). Fear of falling: Efficacy of virtual reality associated with serious games in elderly people. Neuropsychiatr. Dis. Treat..

[120] Li Y., Wilke C., Shiyanov I., Muschalla B. (2024). Impact of Virtual Reality-Based Group Activities on Activity Level and Well-Being Among Older Adults in Nursing Homes: Longitudinal Exploratory Study. JMIR Serious Games.

[121] Liao Y.Y., Chen I.H., Wang R.Y. (2019). Effects of Kinect-based exergaming on frailty status and physical performance in prefrail and frail elderly: A randomized controlled trial. Sci. Rep..

[122] Lima A.M.N., Moreira M.T., Ferreira M.S., Parola V., Rodrigues S., Gomes J.M.P.A., Pinto A.C.C.B., Martins M.M., Moreira S., Fernandes C.S. (2025). Efficacy of Nintendo Switch Rehabilitation Program for Older Adults: A Quasi-Experimental Study. Games Health J..

[123] Litz E., Werner C., Bauer J.M., Hauer K. (2021). Effects and Feasibility of a Memory Exergame Training in Older Adults: A Randomized Controlled Trial. Games Health J..

[124] Liu J., Lockhart T.E., Parijat P., McIntosh J.D., Chiu Y.P. (2015). Comparison of Slip Training in VR Environment and on Moveable Platform. Biomed. Sci. Instrum..

[125] Lund H.H., Jessen J.D. (2014). Effects of Short-Term Training of Community-Dwelling Elderly with Modular Interactive Tiles. Games Health J..

[126] Martins A.C., Guia D., Saraiva M., Pereira T. (2020). Effects of A “Modified” Otago Exercise Program on the Functional Abilities and Social Participation of Older Adults Living in the Community-The AGA@4life Model. Int. J. Environ. Res. Public Health.

[127] Mascarenhas C.H.M., Carneiro J.A.O., Nobre T.T.X., Schettino L., de Araujo C.M., Dos Reis L.A., Fernandes M.H. (2023). Analysis of Plantar Tactile Sensitivity in Older Women after Conventional Proprioceptive Training and Exergame. Int. J. Environ. Res. Public Health.

[128] Merriman N.A., Roudaia E., Ondřej J., Romagnoli M., Orvieto I., O’Sullivan C., Newell F.N. (2022). “CityQuest,” A Custom-Designed Serious Game, Enhances Spatial Memory Performance in Older Adults. Front. Aging Neurosci..

[129] Mirelman A., Rochester L., Maidan I., Del Din S., Alcock L., Nieuwhof F., Rikkert M.O., Bloem B.R., Pelosin E., Avanzino L. (2016). Addition of a non-immersive virtual reality component to treadmill training to reduce fall risk in older adults (V-TIME): A randomised controlled trial. Lancet.

[130] Mohamed Zein El-AbdeenMohamed D., Noshy Abd El-Aziz Mohamed H., Hassan Abd Elhameed S. (2025). Effect of a home-based physical rehabilitation program via virtual reality on the functional outcomes of frail older adults: A quasi-experimental study. Sci. Rep..

[131] Monteiro-Junior R.S., da Silva Figueiredo L.F., Maciel-Pinheiro P.T., Abud E.L.R., Braga A.E.M.M., Barca M.L., Engedal K., Nascimento O.J.M., Deslandes A.C., Laks J. (2017). Acute effects of exergames on cognitive function of institutionalized older persons: A single-blinded, randomized and controlled pilot study. Aging Clin. Exp. Res..

[132] Montero-Alía P., Miralles-Basseda R., López-Jiménez T., Muñoz-Ortiz L., Jiménez-González M., Prat-Rovira J., Albarrán-Sánchez J.L., Manresa-Domínguez J.M., Andreu-Concha C.M., Rodríguez-Pérez M.C. (2019). Controlled trial of balance training using a video game console in community-dwelling older adults. Age Ageing.

[133] Moreira N.B., Rodacki A.L.F., Costa S.N., Pitta A., Bento P.C.B. (2021). Perceptive-Cognitive and Physical Function in Prefrail Older Adults: Exergaming Versus Traditional Multicomponent Training. Rejuvenation Res..

[134] Morone G., Paolucci T., Luziatelli S., Iosa M., Piermattei C., Zangrando F., Paolucci S., Vulpiani M.C., Saraceni V.M., Baldari C. (2016). Wii Fit is effective in women with bone loss condition associated with balance disorders: A randomized controlled trial. Aging Clin. Exp. Res..

[135] Morrison S., Simmons R., Colberg S.R., Parson H.K., Vinik A.I. (2018). Supervised Balance Training and Wii Fit-Based Exercises Lower Falls Risk in Older Adults With Type 2 Diabetes. J. Am. Med. Dir. Assoc..

[136] Mugueta-Aguinaga I., Garcia-Zapirain B. (2019). Frailty Level Monitoring and Analysis after a Pilot Six-Week Randomized Controlled Clinical Trial Using the FRED Exergame Including Biofeedback Supervision in an Elderly Day Care Centre. Int. J. Environ. Res. Public Health.

[137] Nobari H., Rezaei S., Sheikh M., Fuentes-García J.P., Pérez-Gómez J. (2021). Effect of Virtual Reality Exercises on the Cognitive Status and Dual Motor Task Performance of the Aging Population. Int. J. Environ. Res. Public Health.

[138] Oesch P., Kool J., Fernandez-Luque L., Brox E., Evertsen G., Civit A., Hilfiker R., Bachmann S. (2017). Exergames versus self-regulated exercises with instruction leaflets to improve adherence during geriatric rehabilitation: A randomized controlled trial. BMC Geriatr..

[139] Orsega-Smith E., Davis J., Slavish K., Gimbutas L. (2012). Wii Fit Balance Intervention in Community-Dwelling Older Adults. Games Health J..

[140] Parijat P., Lockhart T.E., Liu J. (2015). Effects of perturbation-based slip training using a virtual reality environment on slip-induced falls. Ann. Biomed. Eng..

[141] Peng H.T., Tien C.W., Lin P.S., Peng H.Y., Song C.Y. (2020). Novel Mat Exergaming to Improve the Physical Performance, Cognitive Function, and Dual-Task Walking and Decrease the Fall Risk of Community-Dwelling Older Adults. Front. Psychol..

[142] Perrot A., Maillot P., Le Foulon A., Rebillat A.S. (2021). Effect of Exergaming on Physical Fitness, Functional Mobility, and Cognitive Functioning in Adults With Down Syndrome. Am. J. Intellect. Dev. Disabil..

[143] Pichierri G., Coppe A., Lorenzetti S., Murer K., de Bruin E.D. (2012). The effect of a cognitive-motor intervention on voluntary step execution under single and dual task conditions in older adults: A randomized controlled pilot study. Clin. Interv. Aging.

[144] Pichierri G., Murer K., de Bruin E.D. (2012). A cognitive-motor intervention using a dance video game to enhance foot placement accuracy and gait under dual task conditions in older adults: A randomized controlled trial. BMC Geriatr..

[145] Pitta A., Pereira G., Lara J.P.R., Beck J.K.M., Wolf R., Mayor J.J.V., Moreira N.B., Rodacki A.L.F. (2020). The Effects of Different Exergame Intensity Training on Walking Speed in Older Women. Games Health J..

[146] Prasertsakul T., Kaimuk P., Chinjenpradit W., Limroongreungrat W., Charoensuk W. (2018). The effect of virtual reality-based balance training on motor learning and postural control in healthy adults: A randomized preliminary study. Biomed. Eng. Online.

[147] Pyatova Y., Zhang B., Misawa M., Markowitz S.N., Sen A., Appel L., Cheung K., Nasir P., Tchao D., Garcia-Giler E. (2026). A Visual Telerehabilitation Program in Virtual Reality for Age-Related Macular Degeneration: Randomized Feasibility and Proof-of-Concept Trial. JMIR Rehabil. Assist. Technol..

[148] Quatorze J., Reis M., Alvarez G., Martins A.C. (2026). Exergaming for Healthy Aging: Associations with Functional Capacity, Social Participation, Self-Efficacy for Exercise, and Adherence to Inform Exergame Development. Sensors.

[149] Ramnath U., Rauch L., Lambert E.V., Kolbe-Alexander T. (2021). Efficacy of interactive video gaming in older adults with memory complaints: A cluster-randomized exercise intervention. PLoS ONE.

[150] Rogan S., Radlinger L., Baur H., Schmidtbleicher D., de Bie R.A., de Bruin E.D. (2016). Sensory-motor training targeting motor dysfunction and muscle weakness in long-term care elderly combined with motivational strategies: A single blind randomized controlled study. Eur. Rev. Aging Phys. Act..

[151] Sadeghi H., Hakim M.N., Hamid T.A., Amri S.B., Razeghi M., Farazdaghi M., Shakoor E. (2017). The effect of exergaming on knee proprioception in older men: A randomized controlled trial. Arch. Gerontol. Geriatr..

[152] Sadura-Sieklucka T., Czerwosz L.T., Kądalska E., Kożuchowski M., Księżopolska-Orłowska K., Targowski T. (2023). Is Balance Training Using Biofeedback Effective in the Prophylaxis of Falls in Women over the Age of 65?. Brain Sci..

[153] Santos G.O.R., Wolf R., Silva M.M., Rodacki A.L.F., Pereira G. (2019). Does exercise intensity increment in exergame promote changes in strength, functional capacity and perceptual parameters in pre-frail older women? A randomized controlled trial. Exp. Gerontol..

[154] Saredakis D., Keage H.A., Corlis M., Ghezzi E.S., Loffler H., Loetscher T. (2021). The Effect of Reminiscence Therapy Using Virtual Reality on Apathy in Residential Aged Care: Multisite Nonrandomized Controlled Trial. J. Med. Internet Res..

[155] Schwenk M., Grewal G.S., Honarvar B., Schwenk S., Mohler J., Khalsa D.S., Najafi B. (2014). Interactive balance training integrating sensor-based visual feedback of movement performance: A pilot study in older adults. J. Neuroeng. Rehabil..

[156] Schättin A., Arner R., Gennaro F., de Bruin E.D. (2016). Adaptations of Prefrontal Brain Activity, Executive Functions, and Gait in Healthy Elderly Following Exergame and Balance Training: A Randomized-Controlled Study. Front. Aging Neurosci..

[157] Schättin A., Baier C., Mai D., Klamroth-Marganska V., Herter-Aeberli I., de Bruin E.D. (2019). Effects of exergame training combined with omega-3 fatty acids on the elderly brain: A randomized double-blind placebo-controlled trial. BMC Geriatr..

[158] Self M.A., Pearce L.M.N., Cashin A.G., van den Berg M.E.L., Sherrington C., Hassett L. (2025). Mechanism evaluation of a digitally enabled rehabilitation intervention for people in aged care and neurological rehabilitation: Mediation analysis of the AMOUNT trial. Disabil. Rehabil..

[159] Shin D., Hong S. (2024). A Study on the Effectiveness of VR Rehabilitation Training Content for Older Individuals with Total Knee Replacement: Pilot Study. Healthcare.

[160] Sokołowska B., Świderski W., Smolis-Bąk E., Sokołowska E., Sadura-Sieklucka T. (2024). A machine learning approach to evaluate the impact of virtual balance/cognitive training on fall risk in older women. Front. Comput. Neurosci..

[161] Song S., Hahm S.C., Chang S., Lee J., Kang H., Lee S.H., Kim J. (2026). Feasibility and Preliminary Effects of a Pressure Sensor-Based Exergame on Cognitive and Sensorimotor Frailty in Low-Income Older Adults Receiving Public Community Care Services: Pilot Randomized Controlled Trial. JMIR Aging.

[162] Stanmore E., Eost-Telling C., Meekes W., Banham K., Chillala J., Roy B., Firth J. (2024). Exergames for falls prevention in sheltered homes: A feasibility study. Front. Public Health.

[163] Stephan A.T., McVey A., Steele T., Cooper K., Phillips C.B., Ross L.A. (2024). Older Adults’ Perceptions of Cognitive, Physical, and Exergame Training: A Mixed Methods Investigation of a Four-Armed Randomized Controlled Pilot Trial. Games Health J..

[164] Sugiyama K., Kataoka M., Iwata A., Higuchi Y., Saga R., Ikebuchi M., Takahashi S., Nakamura H. (2026). Feasibility and safety of mixed reality treadmill walking in older adults: A pilot and feasibility trial. Disabil. Rehabil. Assist. Technol..

[165] Szczepocka E., Mokros Ł., Kazmierski J., Nowakowska K., Łucka A., Antoszczyk A., Oltra-Cucarella J., Werzowa W., Hellevik M.M., Skouras S. (2024). The Effectiveness of Virtual Reality-Based Training on Cognitive, Social, and Physical Functioning in High-Functioning Older Adults (CoSoPhy FX): 2-Arm, Parallel-Group Randomized Controlled Trial. JMIR Res. Protoc..

[166] Szturm T., Betker A.L., Moussavi Z., Desai A., Goodman V. (2011). Effects of an interactive computer game exercise regimen on balance impairment in frail community-dwelling older adults: A randomized controlled trial. Phys. Ther..

[167] Taylor L., Kerse N., Klenk J., Borotkanics R., Maddison R. (2018). Exergames to Improve the Mobility of Long-Term Care Residents: A Cluster Randomized Controlled Trial. Games Health J..

[168] Wherry S.J., Ananian C., Swan P.D. (2019). Feasibility of a Home-Based Balance Intervention in Middle-Aged Women Using Wii Fit Plus^®^. J. Phys. Act. Health.

[169] Wilson O., Borris F., Thorpe B., Newby A., El Hakour Y., Douglas B., Butler J., Melendez R.R., Azikiwe C., Arnold N. (2024). Exploring Virtual Reality-Based Training’s Effects on Balance Ability and Balance Confidence in Older Adults. Annu. Int. Conf. IEEE Eng. Med. Biol. Soc..

[170] Wilson O., Arnold N., Thompson L.A. (2024). Investigating the Effects of Virtual Reality-Based Training on Balance Ability and Balance Confidence in Older Individuals. Appl. Sci..

[171] Wong A.K.C., Zhang M.Q., Bayuo J., Chow K.K.S., Wong S.M., Wong B.P., Liu B.C.M., Lau D.C.H., Kowatsch T. (2024). The Effect of Young People-Assisted, Individualized, Motion-Based Video Games on Physical, Cognitive, and Social Frailty Among Community-Dwelling Older Adults With Frailty: Randomized Controlled Trial. JMIR Serious Games.

[172] Wu Y.Z., Lin J.Y., Wu P.L., Kuo Y.F. (2019). Effects of a hybrid intervention combining exergaming and physical therapy among older adults in a long-term care facility. Geriatr. Gerontol. Int..

[173] Yeşilyaprak S.S., Yıldırım M.Ş., Tomruk M., Ertekin Ö., Algun Z.C. (2016). Comparison of the effects of virtual reality-based balance exercises and conventional exercises on balance and fall risk in older adults living in nursing homes in Turkey. Physiother. Theory Pract..

[174] Yousefi Babadi S., Daneshmandi H. (2021). Effects of virtual reality versus conventional balance training on balance of the elderly. Exp. Gerontol..

[175] Zahedian-Nasab N., Jaberi A., Shirazi F., Kavousipor S. (2021). Effect of virtual reality exercises on balance and fall in elderly people with fall risk: A randomized controlled trial. BMC Geriatr..

[176] Zukowski L.A., Shaikh F.D., Haggard A.V., Hamel R.N. (2022). Acute effects of virtual reality treadmill training on gait and cognition in older adults: A randomized controlled trial. PLoS ONE.

[177] de Bruin E.D., Baur H., Brülhart Y., Luijckx E., Hinrichs T., Rogan S. (2020). Combining Stochastic Resonance Vibration With Exergaming for Motor-Cognitive Training in Long-Term Care; A Sham-Control Randomized Controlled Pilot Trial. Front. Med..

[178] de Rond V., D’Cruz N., Hulzinga F., McCrum C., Verschueren S., de Xivry J.O., Nieuwboer A. (2023). Neural correlates of weight-shift training in older adults: A randomized controlled study. Sci. Rep..

[179] Abd El-Kafy E.M., Alayat M.S., Subahi M.S., Badghish M.S. (2024). Motion tracking virtual reality technology in improving gait in the elderly: A randomized controlled trial. Clin. Rehabil..

[180] AlQahtani M. (2024). Impact of Wii Fit Training (WFT) and Reactive Balance Training (RBT) for in Elder Age Population: A Comparative Analysis. J. Pharm. Bioallied Sci..

[181] Bacha J.M.R., Gomes G.C.V., de Freitas T.B., Viveiro L.A.P., da Silva K.G., Bueno G.C., Varise E.M., Torriani-Pasin C., Alonso A.C., Luna N.M.S. (2018). Effects of Kinect Adventures Games Versus Conventional Physical Therapy on Postural Control in Elderly People: A Randomized Controlled Trial. Games Health J..

[182] Bieryla K.A. (2016). Xbox Kinect training to improve clinical measures of balance in older adults: A pilot study. Aging Clin. Exp. Res..

[183] Campelo A.M., Weisberg A., Sheehan D.P., Schneider K., Cossich V.R.A., Katz L. (2023). Physical and Affective Physical Literacy Domains Improved After a Six-Week Exergame Exercise Program in Older Adults: A Randomized Controlled Clinical Trial. Games Health J..

[184] Chan W.L.S., Chan C.W.L., Lam F.M.H., Chan H.H.W., Chan K.C.K., Chan J.S.K., Chan O.L.W., Cheung D.S.K. (2024). Feasibility, safety, and effects of a Nintendo Ring Fit Adventure™ balance and strengthening exercise program in community-dwelling older adults with a history of falls: A feasibility randomized controlled trial. Geriatr. Gerontol. Int..

[185] Chang C.L., Chen W.Y., Pan C.Y., Tseng Y.T., Chen F.C., Tsai C.L. (2026). The Effects of 8-Week Traditional Aerobic Exercise and Exergaming on Dual-Task Performance and Prefrontal Cortex Activation in Older Adults. Med. Sci. Sports Exerc..

[186] Chuang I.C., Abdullahi A., Chen I.C., Wu Y.R., Wu C.Y. (2025). Effects of immersive leisure-based virtual reality cognitive training on cognitive and physical function in community-based older adults: A randomized controlled trial. Digit. Health.

[187] Duque G., Boersma D., Loza-Diaz G., Hassan S., Suarez H., Geisinger D., Suriyaarachchi P., Sharma A., Demontiero O. (2013). Effects of balance training using a virtual-reality system in older fallers. Clin. Interv. Aging.

[188] Evangelista de Lima B., Passos G.S., Youngstedt S.D., Bandeira Santos Júnior L.C., Gonçalves Santana M. (2021). Effects of Xbox Kinect exercise training on sleep quality, anxiety and functional capacity in older adults. J. Bodyw. Mov. Ther..

[189] Fidan O., Kiloatar H., Colak E., Oskay D. (2025). Effects of group-based virtual reality training on activities of daily living and functional outcomes in older adults: A randomised control trial. Disabil. Rehabil. Assist. Technol..

[190] Gallardo-Meza C., Simon K., Bustamante-Ara N., Ramirez-Campillo R., García-Pinillos F., Keogh J.W.L., Izquierdo M. (2022). Effects of 4 Weeks of Active Exergames Training on Muscular Fitness in Elderly Women. J. Strength Cond. Res..

[191] Ghous M., Masood Q., Nawaz Malik A., Afridi A., Mehmood Q. (2024). Comparison of Nonimmersive Virtual Reality and Task-Oriented Circuit Training on Gait, Balance, and Cognition Among Elderly Population: A Single-Blind Randomized Control Trial. Games Health J..

[192] Gonçalves A., Muñoz J., Cameirão M.S., Gouveia É.R., Sousa H., Bermúdez I., Badia S. (2021). The Benefits of Custom Exergames for Fitness, Balance, and Health-Related Quality of Life: A Randomized Controlled Trial with Community-Dwelling Older Adults. Games Health J..

[193] Guede-Rojas F., Medel-Gutiérrez M.J., Cárcamo-Vargas M., Soto-Martínez A., Chirosa Ríos L.J., Ramirez-Campillo R., Álvarez C., Jerez-Mayorga D. (2023). Effects of Exergames and Conventional Physical Therapy on Functional Physical Performance in Older Adults: A Randomized Controlled Trial. Games Health J..

[194] Hou H.Y., Li H.J. (2022). Effects of exergame and video game training on cognitive and physical function in older adults: A randomized controlled trial. Appl. Ergon..

[195] Jung D.I., Ko D.S., Jeong M.A. (2015). Kinematic effect of Nintendo Wii(TM) sports program exercise on obstacle gait in elderly women with falling risk. J. Phys. Ther. Sci..

[196] Karahan A.Y., Tok F., Taşkın H., Kuçuksaraç S., Başaran A., Yıldırım P. (2015). Effects of Exergames on Balance, Functional Mobility, and Quality of Life of Geriatrics Versus Home Exercise Programme: Randomized Controlled Study. Cent. Eur. J. Public Health.

[197] Khushnood K., Sultan N., Altaf S., Qureshi S., Mehmood R., Awan M.M.A. (2021). Effects of Wii Fit exer-gaming on balance and gait in elderly population: A randomized control trial. J. Pak. Med. Assoc..

[198] Lima Rebêlo F., de Souza Silva L.F., Doná F., Sales Barreto A., de Souza Siqueira Quintans J. (2021). Immersive virtual reality is effective in the rehabilitation of older adults with balance disorders: A randomized clinical trial. Exp. Gerontol..

[199] Morat M., Bakker J., Hammes V., Morat T., Giannouli E., Zijlstra W., Donath L. (2019). Effects of stepping exergames under stable versus unstable conditions on balance and strength in healthy community-dwelling older adults: A three-armed randomized controlled trial. Exp. Gerontol..

[200] Padala K.P., Padala P.R., Lensing S.Y., Dennis R.A., Bopp M.M., Parkes C.M., Garrison M.K., Dubbert P.M., Roberson P.K., Sullivan D.H. (2017). Efficacy of Wii-Fit on Static and Dynamic Balance in Community Dwelling Older Veterans: A Randomized Controlled Pilot Trial. J. Aging Res..

[201] Park E.C., Kim S.G., Lee C.W. (2015). The effects of virtual reality game exercise on balance and gait of the elderly. J. Phys. Ther. Sci..

[202] Pluchino A., Lee S.Y., Asfour S., Roos B.A., Signorile J.F. (2012). Pilot study comparing changes in postural control after training using a video game balance board program and 2 standard activity-based balance intervention programs. Arch. Phys. Med. Rehabil..

[203] Sadeghi H., Jehu D.A., Daneshjoo A., Shakoor E., Razeghi M., Amani A., Hakim M.N., Yusof A. (2021). Effects of 8 Weeks of Balance Training, Virtual Reality Training, and Combined Exercise on Lower Limb Muscle Strength, Balance, and Functional Mobility Among Older Men: A Randomized Controlled Trial. Sports Health.

[204] Sato K., Kuroki K., Saiki S., Nagatomi R. (2015). Improving Walking, Muscle Strength, and Balance in the Elderly with an Exergame Using Kinect: A Randomized Controlled Trial. Games Health J..

[205] Shamsoddini A., Cheraghifard M. (2026). Impact of virtual reality versus conventional balance training on balance function and fear of falling in older adults: A single blinded, randomized controlled trial. Eur. Geriatr. Med..

[206] Sheehy L., Bharadwaj L., Nissen K.A., Estey J.L. (2025). Non-Immersive Virtual Reality Exercise Can Increase Exercise in Older Adults Living in the Community and in Long-Term Care: A Randomized Controlled Trial. Clin. Interv. Aging.

[207] Tollár J., Nagy F., Moizs M., Tóth B.E., Sanders L.M.J., Hortobágyi T. (2019). Diverse Exercises Similarly Reduce Older Adults’ Mobility Limitations. Med. Sci. Sports Exerc..

[208] Toulotte C., Toursel C., Olivier N. (2012). Wii Fit^®^ training vs. Adapted Physical Activities: Which one is the most appropriate to improve the balance of independent senior subjects? A randomized controlled study. Clin. Rehabil..

[209] Yang C.M., Chen Hsieh J.S., Chen Y.C., Yang S.Y., Lin H.K. (2020). Effects of Kinect exergames on balance training among community older adults: A randomized controlled trial. Medicine.

[210] Yang D.J., Lee H.S. (2026). Exergame-based cognitive-motor integrated training: A novel approach to improving physical functions in older adults. Hong. Kong Physiother. J..

[211] Yang D.J., Lee H.S. (2026). Effects of a 3D motion recognition-based virtual training system on balance and gait performance in older adults. Phys. Act. Nutr..

[212] Yue T., Yan X., Lv Y., Ren X., Zhang S., Qi F. (2025). Effects of Immersive Virtual Reality-Based Tennis Training on Balance in Older Adults. Gerontology.

[213] Anson E., Ma L., Meetam T., Thompson E., Rathore R., Dean V., Jeka J. (2018). Trunk motion visual feedback during walking improves dynamic balance in older adults: Assessor blinded randomized controlled trial. Gait Posture.

[214] Béraud-Peigné N., Maillot P., Perrot A. (2024). The effects of a new immersive multidomain training on cognitive, dual-task and physical functions in older adults. Geroscience.

[215] Cho G.H., Hwangbo G., Shin H.S. (2014). The Effects of Virtual Reality-based Balance Training on Balance of the Elderly. J. Phys. Ther. Sci..

[216] Dos Santos R.G., de Freitas J.V.R., Alcantara L.M., Lopes G.G.C., Galvão L.L., de Queiroz B.M., Santos D.A.T., de Lira C.A.B., Andrade M.S., Knechtle B. (2023). Effects of twelve weeks of dance Exergames on the quality-of-life variables of elderly Brazilian women enrolled in a community program. J. Bodyw. Mov. Ther..

[217] Jiang Y., Oide K., Chow Y.E., Chen C., Zhang M., Chua M.C.H., Yoong S.Q. (2025). Effectiveness of an Artificial Intelligence-Infused SinDance Exergame for Enhancing Physical Functions and Well-Being in Older Adults: A Pilot Randomized Controlled Trial. J. Am. Med. Dir. Assoc..

[218] Lee Y., Choi W., Lee K., Song C., Lee S. (2017). Virtual Reality Training With Three-Dimensional Video Games Improves Postural Balance and Lower Extremity Strength in Community-Dwelling Older Adults. J. Aging Phys. Act..

[219] Maillot P., Perrot A., Hartley A. (2012). Effects of interactive physical-activity video-game training on physical and cognitive function in older adults. Psychol. Aging.

[220] Singh Y., Vashista V. (2026). Visuomotor coordination training in dual-motor task improves balance recovery while responding to visuospatial perturbations in older adults. Hum. Mov. Sci..

[221] Sápi M., Domján A., Fehérné Kiss A., Pintér S. (2019). Is Kinect Training Superior to Conventional Balance Training for Healthy Older Adults to Improve Postural Control?. Games Health J..

[222] Uematsu A., Tsuchiya K., Fukushima H., Hortobágyi T. (2023). Effects of Motor-Cognitive Dual-Task Standing Balance Exergaming Training on Healthy Older Adults’ Standing Balance and Walking Performance. Games Health J..

[223] Abd El-Kafy E.M., Alayat M.S., Subahi M.S., Badghish M.S. (2024). C-Mill Virtual Reality/Augmented Reality Treadmill Training for Reducing Risk of Fall in the Elderly: A Randomized Controlled Trial. Games Health J..

[224] Chan W.L.S., Chan C.W.L., Chan H.H.W., Chan K.C.K., Chan J.S.K., Chan O.L.W. (2024). A randomised controlled pilot study of a Nintendo Ring Fit Adventure™ balance and strengthening exercise program in community-dwelling older adults with a history of falls. Australas. J. Ageing.

[225] Khushnood K., Altaf S., Sultan N., Ali Awan M.M., Mehmood R., Qureshi S. (2021). Role Wii Fit exer-games in improving balance confidence and quality of life in elderly population. J. Pak. Med. Assoc..

[226] Maillot P., Perrot A., Hartley A., Do M.C. (2014). The braking force in walking: Age-related differences and improvement in older adults with exergame training. J. Aging Phys. Act..

