# Supplementary material for: Effects of Virtual Reality Interventions on Balance in Community-Dwelling Older Adults: A Systematic Review
Source: J Clin Med. 2026 Oct 27;15(21):8368. doi: 10.3390/jcm15218368 (PMC13764286; doi:10.3390/jcm15218368)
Supplement: Supplementary file 1 [file jcm-15-08368-s001.zip › jcm-4570649-supplementary.pdf]

Supplementary Table S1. Search runs, strategies and numbers of records retrieved

All databases were searched from inception with no date limits; only reports in English were eligible.

Run	Database	Strategy	Records retrieved
1 (July 2023) and 2 (January 2025)	PubMed (including MEDLINE)	Population block as reported in the original submission (frailty-specific terms) combined with the VR/exergaming block shown below	896 (to January 2025)
1 and 2	Embase	As above, translated to Emtree and free-text terms	186 (to January 2025)
1 and 2	CINAHL	As above, translated to CINAHL headings and free-text terms	133 (to January 2025)
1 and 2	PEDro	Free-text terms for VR/exergaming; subdiscipline gerontology; method clinical trial	26 (to January 2025)
3 (update, 8 October 2026)	PubMed (including MEDLINE)	("Aged"[MeSH] OR "older adult*"[tiab] OR elderly[tiab] OR "community-dwelling"[tiab]) AND ("Virtual Reality"[MeSH] OR "virtual reality"[tiab] OR "video game*"[tiab] OR exergam*[tiab] OR "Wii Fit"[tiab] OR Nintendo[tiab] OR Xbox[tiab] OR Kinect[tiab] OR "virtual world"[tiab]) AND English[lang]	4,128 (from inception)

Runs 1 and 2: 1,241 records in total (138 duplicates). Run 3: Embase, CINAHL and PEDro were not re-searched. Outcome of run 3: 3,928 records excluded on title/abstract; 200 assessed for eligibility; 15 already included; 122 excluded with reasons (Table S2); 63 potentially eligible, of which 15 were confirmed eligible in full text and incorporated in this review (Tables 2–4; Table S4) and 48 are awaiting classification (Table S5).

Supplementary Table S2. Reports excluded at eligibility assessment in the updated PubMed search, with reasons

Eligibility was assessed against the criteria in Section 2.2, using the full text where accessible (marked FT) and otherwise the abstract (marked Ab). Reasons for the 76 exclusions at the first full-text screening of the original search were not recorded at report level.

No.	Report	Reason for exclusion	Basis
1	Alhasan HS et al. (2021) [57]. Application of Interactive Video Games as Rehabilitation Tools to Improve Postural Control and Risk of Falls in Prefrail Older Adults. Cyborg Bionic Syst. PMID 36285138; doi:10.34133/2021/9841342	Sample recruited as frail or pre-frail	Ab
2	Alhasan HS et al. (2025) [58]. Gaming Against Frailty: Effects of Virtual Reality-Based Training on Postural Control, Mobility, and Fear of Falling Among Frail Older Adults. J Clin Med. PMID 40807152; doi:10.3390/jcm14155531	Sample recruited as frail or pre-frail	Ab
3	Anguera JA et al. (2022) [59]. Integrated cognitive and physical fitness training enhances attention abilities in older adults. NPJ Aging. PMID 36042247; doi:10.1038/s41514-022-00093-y	No balance outcome (attention)	Ab
4	Areiza R et al. (2026) [60]. Physical Capacity Changes Following a Virtual Reality-Assisted Exercise Program in Institutionalized Older Adults: A Pilot Intervention Study. Healthcare (Basel). PMID 42651241; doi:10.3390/healthcare14162472	Institutionalised or residential-care sample (nursing home, long-term care, hostel, inpatient); No control group	Ab
5	Arnold N et al. (2024) [61]. Virtual Reality Training Affects Center of Pressure (COP)-Based Balance Parameters in Older Individuals. Appl Sci (Basel). PMID 41000324; doi:10.3390/app14167182	Not randomised (allocation method not reported); VR headset without movement-mapped task	FT
6	Bakker J et al. (2020) [62]. Balance training monitoring and individual response during unstable vs. stable balance Exergaming in elderly adults: Findings from a randomized controlled trial. Exp Gerontol. PMID 32730797; doi:10.1016/j.exger.2020.111037	Secondary analysis of a trial listed as eligible (Morat 2019)	Ab
7	Batcir S et al. (2021) [63]. Development and piloting of a perturbation stationary bicycle robotic system that provides unexpected lateral perturbations during bicycling (the PerStBiRo system). BMC Geriatr. PMID 33478400; doi:10.1186/s12877-021-02015-1	Intervention does not meet the operational definition of VR	Ab
8	Begde A et al. (2023) [64]. The Effectiveness of Home-Based Exergames Training on Cognition and Balance in Older Adults: A Comparative Quasi-Randomized Study of Two Exergame Interventions. Innov Aging. PMID 37941829; doi:10.1093/geroni/igad102	Not randomised (non-randomised or quasi-randomised allocation, or randomisation not reported)	Ab
9	Belleville S et al. (2023) [65]. Pre-frail older adults show improved cognition with StayFitLonger computerized home-based training: a randomized controlled trial. Geroscience. PMID 36266559; doi:10.1007/s11357-022-00674-5	Intervention does not meet the operational definition of VR (tablet-delivered multidomain programme); cognitive outcomes	Ab
10	Blanco V et al. (2025) [66]. Efficacy of a video game for promoting active and healthy aging (Initial Version): a randomized controlled trial. BMC Public Health. PMID 41088013; doi:10.1186/s12889-025-23792-z	Intervention does not meet the operational definition of VR; Not older adults (mean age < 60 years)	Ab
11	Bourrelier J et al. (2021) [67]. Enhancement of Anticipatory Postural Adjustments by Virtual Reality in Older Adults with Cognitive and Motor Deficits: A Randomised Trial. Geriatrics (Basel). PMID 34449605; doi:10.3390/geriatrics6030072	Sample recruited as frail or pre-frail	Ab
12	Cai H et al. (2019) [68]. Effect of Low-Intensity, Kinect™-Based Kaimai-Style Qigong Exercise in Older Adults With Type 2 Diabetes. J Gerontol Nurs. PMID 30690653; doi:10.3928/00989134-20190111-05	Sample defined by a specific diagnosis (type 2 diabetes); Not randomised (non-randomised or quasi-randomised allocation, or randomisation not reported)	Ab
13	Campo-Prieto P et al. (2022) [69]. Immersive Virtual Reality as a Novel Physical Therapy Approach for Nonagenarians: Usability and Effects on Balance Outcomes of a Game-Based Exercise Program. J Clin Med. PMID 35807196; doi:10.3390/jcm11133911	Institutionalised or residential-care sample (participants recruited from an old people's home)	FT
14	Campo-Prieto P et al. (2022) [70]. Feasibility and Effects of an Immersive Virtual Reality Exergame Program on Physical Functions in Institutionalized Older Adults: A Randomized Clinical Trial. Sensors (Basel). PMID 36146092; doi:10.3390/s22186742	Institutionalised or residential-care sample (nursing home, long-term care, hostel, inpatient)	Ab
15	Chen PY et al. (2012) [71]. Lower limb power rehabilitation (LLPR) using interactive video game for improvement of balance function in older people. Arch Gerontol Geriatr. PMID 22795360; doi:10.1016/j.archger.2012.05.012	Not randomised (non-randomised or quasi-randomised allocation, or randomisation not reported)	Ab
16	Chow YE et al. (2025) [72]. Older adults' perceptions and experiences of the SinDance exergame: A process evaluation. Geriatr Nurs. PMID 41108898; doi:10.1016/j.gerinurse.2025.103660	No balance outcome (process evaluation)	Ab
17	Cicek A et al. (2020) [73]. Interactive video game-based approaches improve mobility and mood in older adults: A nonrandomized, controlled trial. J Bodyw Mov Ther. PMID 32825997; doi:10.1016/j.jbmt.2020.01.005	Institutionalised or residential-care sample (nursing home, long-term care, hostel, inpatient); Not randomised (non-randomised or quasi-randomised allocation, or randomisation not reported)	Ab
18	Ciemer C et al. (2025) [74]. Effectiveness, enjoyment, and meaningfulness of a virtual reality gait-based fall prevention exergame in community-dwelling healthy older adults: an interdisciplinary pilot study. Front Psychol. PMID 40567879; doi:10.3389/fpsyg.2025.1610377	Not randomised (non-randomised or quasi-randomised allocation, or randomisation not reported)	Ab
19	Corrêa FI et al. (2024) [75]. The effect of transcranial direct current stimulation associated with video game training on the postural balance of older women in the community: A blind, randomized, clinical trial. Physiother Res Int. PMID 37608641; doi:10.1002/pri.2046	No non-VR comparator (VR in all arms)	Ab
20	Damasceno SO et al. (2026) [76]. Responses in Posttherapy Recovery-Based Virtual Reality With Blood Flow Restriction in Older Women: A Randomized Crossover Clinical Trial. J Aging Phys Act. PMID 41052761; doi:10.1123/japa.2025-0027	Single-session (acute) exposure rather than a training intervention	Ab
21	Daniel K (2012) [77]. Wii-hab for pre-frail older adults. Rehabil Nurs. PMID 22744992; doi:10.1002/rnj.25	Sample recruited as frail or pre-frail	Ab
22	Del Din S et al. (2020) [78]. Falls Risk in Relation to Activity Exposure in High-Risk Older Adults. J Gerontol A Biol Sci Med Sci. PMID 31942969; doi:10.1093/gerona/glaa007	Sample defined by a specific diagnosis (V-TIME secondary analysis)	Ab
23	Delbroek T et al. (2017) [79]. The effect of cognitive-motor dual task training with the biorescue force platform on cognition, balance	Institutionalised or residential-care sample (nursing home, long-term care, hostel,	Ab

No.	Report	Reason for exclusion	Basis
	and dual task performance in institutionalized older adults: a randomized controlled trial. J Phys Ther Sci. PMID 28744033; doi:10.1589/jpts.29.1137	inpatient)	
24	Ditchburn JL et al. (2020) [80]. The effects of exergaming on pain, postural control, technology acceptance and flow experience in older people with chronic musculoskeletal pain: a randomised controlled trial. BMC Sports Sci Med Rehabil. PMID 33062284; doi:10.1186/s13102-020-00211-x	Sample defined by a specific diagnosis (chronic musculoskeletal pain)	Ab
25	Dockx K et al. (2017) [81]. Fall-Prone Older People's Attitudes towards the Use of Virtual Reality Technology for Fall Prevention. Gerontology. PMID 28817828; doi:10.1159/000479085	No balance outcome (attitudes; V-TIME trial)	Ab
26	Eggenberger P et al. (2015) [82]. Does multicomponent physical exercise with simultaneous cognitive training boost cognitive performance in older adults? A 6-month randomized controlled trial with a 1-year follow-up. Clin Interv Aging. PMID 26316729; doi:10.2147/CIA.S87732	No balance outcome (cognitive report of included trial [37])	Ab
27	Eggenberger P et al. (2016) [83]. Exergame and Balance Training Modulate Prefrontal Brain Activity during Walking and Enhance Executive Function in Older Adults. Front Aging Neurosci. PMID 27148041; doi:10.3389/fnagi.2016.00066	No balance outcome (prefrontal activity and executive function)	Ab
28	Eggenberger P et al. (2020) [84]. Heart Rate Variability Mainly Relates to Cognitive Executive Functions and Improves Through Exergame Training in Older Adults: A Secondary Analysis of a 6-Month Randomized Controlled Trial. Front Aging Neurosci. PMID 32760267; doi:10.3389/fnagi.2020.00197	No balance outcome (heart-rate variability report of included trial [37])	Ab
29	Fakhro MA et al. (2020) [85]. Effects of Nintendo Wii fit game training on balance among Lebanese older adults. Aging Clin Exp Res. PMID 31802405; doi:10.1007/s40520-019-01425-x	Institutionalised or residential-care sample (nursing home, long-term care, hostel, inpatient)	Ab
30	Filho JM et al. (2022) [86]. Effects of multidomain interventions on skeletal muscle architecture and function in pre-frail older women: The WiiProtein study. Geriatr Nurs. PMID 36332439; doi:10.1016/j.gerinurse.2022.10.009	Sample recruited as frail or pre-frail	Ab
31	Fu AS et al. (2015) [87]. Effectiveness of Exergaming Training in Reducing Risk and Incidence of Falls in Frail Older Adults With a History of Falls. Arch Phys Med Rehabil. PMID 26360975; doi:10.1016/j.apmr.2015.08.427	Institutionalised or residential-care sample (nursing home, long-term care, hostel, inpatient); Sample recruited as frail or pre-frail	Ab
32	Fung V et al. (2012) [88]. Use of Nintendo Wii Fit™ in the rehabilitation of outpatients following total knee replacement: a preliminary randomised controlled trial. Physiotherapy. PMID 22898573; doi:10.1016/j.physio.2012.04.001	Sample defined by a specific diagnosis (total knee replacement)	Ab
33	Gallo LH et al. (2019) [89]. Effects of virtual dance exercise on skeletal muscle architecture and function of community dwelling older women. J Musculoskelet Neuronal Interact. PMID 30839303	Not randomised (non-randomised or quasi-randomised allocation, or randomisation not reported) (self-selected groups)	Ab
34	Gatica-Rojas V et al. (2025) [90]. Telerehabilitation and Face-to-Face Exergame Delivery Modalities to Improve Postural Control in Older Adults: A Randomised Controlled Trial. Med Sci (Basel). PMID 41283271; doi:10.3390/medsci13040270	No non-VR comparator (VR in all arms)	Ab
35	Ghasemian M et al. (2024) [91]. Computerized cognitive games versus cognitive exergame: the comparison of motor and cognitive functions enhancement in the elderly. BMC Psychol. PMID 39394166; doi:10.1186/s40359-024-02064-2	Intervention does not meet the operational definition of VR (light-board reaction training)	Ab
36	Gomes GCV et al. (2018) [92]. Feasibility, safety, acceptability, and functional outcomes of playing Nintendo Wii Fit Plus(TM) for frail older adults: A randomized feasibility clinical trial. Maturitas. PMID 30415751; doi:10.1016/j.maturitas.2018.10.002	Sample recruited as frail or pre-frail	Ab
37	Gouveia ÉR et al. (2021) [93]. The Efficacy of a Multicomponent Functional Fitness Program Based on Exergaming on Cognitive Functioning of Healthy Older Adults: A Randomized Controlled Trial. J Aging Phys Act. PMID 33361495; doi:10.1123/japa.2020-0083	No balance outcome (cognitive report of the same trial)	Ab
38	Gschwind YJ et al. (2015) [94]. The effect of sensor-based exercise at home on functional performance associated with fall risk in older people - a comparison of two exergame interventions. Eur Rev Aging Phys Act. PMID 26865875; doi:10.1186/s11556-015-0156-5	Secondary analysis pooling two exergame trials (no separate trial data)	Ab
39	Hagedoorn L et al. (2025) [95]. Action observation with motor simulation of reactive stepping: A randomized study in older adults with a history of falls. Exp Gerontol. PMID 39622456; doi:10.1016/j.exger.2024.112645	Single-session (acute) exposure rather than a training intervention	Ab
40	Hassett L et al. (2020) [96]. Digitally enabled aged care and neurological rehabilitation to enhance outcomes with Activity and MObility UsiNg Technology (AMOUNT) in Australia: A randomised controlled trial. PLoS Med. PMID 32069288; doi:10.1371/journal.pmed.1003029	Institutionalised or residential-care sample (nursing home, long-term care, hostel, inpatient) (inpatient rehabilitation)	Ab
41	Hauer K et al. (2020) [97]. Effectiveness and sustainability of a motor-cognitive stepping exergame training on stepping performance in older adults: a randomized controlled trial. Eur Rev Aging Phys Act. PMID 33005256; doi:10.1186/s11556-020-00248-4	No balance outcome (outcomes were exergame stepping reaction times and game scores only)	FT
42	He Y et al. (2025) [98]. Virtual obstacle-avoidance training using daily-life obstacles with physical feedback in older people: A cross-over trial. Arch Gerontol Geriatr. PMID 40318297; doi:10.1016/j.archger.2025.105866	Single-session (acute) exposure rather than a training intervention	Ab
43	Huang SF et al. (2025) [99]. A Novel Gamified Exercise Program Incorporating Stampede Training for Enhancing Functional Fitness, Physical Activity Levels, and Quality of Life in Community-Dwelling Older Adults: Randomized Parallel Exploratory Trial. JMIR Serious Games. PMID 41183374; doi:10.2196/73474	Intervention does not meet the operational definition of VR (light-and-sound mat without a computer-generated environment)	FT
44	Hwang NK et al. (2021) [100]. Effects of Semi-Immersive Virtual Reality-Based Cognitive Training Combined with Locomotor Activity on Cognitive Function and Gait Ability in Community-Dwelling Older Adults. Healthcare (Basel). PMID 34203233; doi:10.3390/healthcare9070814	Not randomised (allocation method not reported)	FT
45	Jahouh M et al. (2021) [101]. Impact of an Intervention with Wii Video Games on the Autonomy of Activities of Daily Living and Psychological-Cognitive Components in the Institutionalized Elderly. Int J Environ Res Public Health. PMID 33562249; doi:10.3390/ijerph18041570	Institutionalised or residential-care sample (nursing home, long-term care, hostel, inpatient)	Ab

No.	Report	Reason for exclusion	Basis
46	Janhunen M et al. (2023) [102]. Effects of a home-based, exergaming intervention on physical function and pain after total knee replacement in older adults: a randomised controlled trial. BMJ Open Sport Exerc Med. PMID 36896366; doi:10.1136/bmjsem-2022-001416	Sample defined by a specific diagnosis (total knee replacement)	Ab
47	Janssen S et al. (2013) [103]. A Preliminary Study on the Effectiveness of Exergame Nintendo "Wii Fit Plus" on the Balance of Nursing Home Residents. Games Health J. PMID 26192126; doi:10.1089/g4h.2012.0074	Institutionalised or residential-care sample (nursing home, long-term care, hostel, inpatient); Not randomised (non-randomised or quasi-randomised allocation, or randomisation not reported)	Ab
48	Jeon MJ et al. (2021) [104]. Block and Random Practice: A Wii Fit Dynamic Balance Training in Older Adults. Res Q Exerc Sport. PMID 32401683; doi:10.1080/02701367.2020.1733456	No non-VR comparator (VR in all arms)	Ab
49	Kannan L et al. (2024) [105]. Gaming-Based Tele-Exercise Program to Improve Physical Function in Frail Older Adults: Feasibility Randomized Controlled Trial. J Med Internet Res. PMID 39602215; doi:10.2196/56810	Sample recruited as frail or pre-frail	Ab
50	Kayama H et al. (2013) [106]. Efficacy of an Exercise Game Based on Kinect in Improving Physical Performances of Fall Risk Factors in Community-Dwelling Older Adults. Games Health J. PMID 26192228; doi:10.1089/g4h.2013.0006	Not randomised (non-randomised or quasi-randomised allocation, or randomisation not reported)	Ab
51	Kayama H et al. (2014) [107]. Effect of a Kinect-based exercise game on improving executive cognitive performance in community-dwelling elderly: case control study. J Med Internet Res. PMID 24565806; doi:10.2196/jmir.3108	No balance outcome (cognitive outcomes; same trial as Kayama 2013)	Ab
52	Kim SH et al. (2022) [108]. Benefits of Virtual Reality Program and Motor Imagery Training on Balance and Fall Efficacy in Isolated Older Adults: A Randomized Controlled Trial. Medicina (Kaunas). PMID 36363502; doi:10.3390/medicina58111545	Institutionalised or residential-care sample (nursing home, long-term care, hostel, inpatient) (convalescent hospital)	Ab
53	Kim T et al. (2022) [109]. Effectiveness and Usability of a Novel Kinect-Based Tailored Interactive Fall Intervention System for Fall Prevention in Older People: A Preliminary Study. Front Public Health. PMID 35712291; doi:10.3389/fpubh.2022.884551	Not randomised (non-randomised or quasi-randomised allocation, or randomisation not reported)	Ab
54	Konstantinidis EI et al. (2016) [110]. Design, Implementation, and Wide Pilot Deployment of FitForAll: An Easy to use Exergaming Platform Improving Physical Fitness and Life Quality of Senior Citizens. IEEE J Biomed Health Inform. PMID 26731797; doi:10.1109/JBHI.2014.2378814	Not randomised (non-randomised or quasi-randomised allocation, or randomisation not reported)	Ab
55	Kubica J et al. (2019) [111]. Systematic Balance Exercises Influence Cortical Activation and Serum BDNF Levels in Older Adults. J Clin Med. PMID 31703409; doi:10.3390/jcm8111910	No balance outcome (neuroimaging and BDNF)	Ab
56	Kubicki A et al. (2012) [112]. Practice-related improvements in postural control during rapid arm movement in older adults: a preliminary study. J Gerontol A Biol Sci Med Sci. PMID 21948599; doi:10.1093/gerona/glr148	Not randomised (non-randomised or quasi-randomised allocation, or randomisation not reported)	Ab
57	Kubicki A et al. (2014) [113]. Motor-prediction improvements after virtual rehabilitation in geriatrics: frail patients reveal different learning curves for movement and postural control. Neurophysiol Clin. PMID 24502911; doi:10.1016/j.neucli.2013.10.128	Sample recruited as frail or pre-frail	Ab
58	Kwan RYC et al. (2021) [114]. Feasibility and Effects of Virtual Reality Motor-Cognitive Training in Community-Dwelling Older People With Cognitive Frailty: Pilot Randomized Controlled Trial. JMIR Serious Games. PMID 34383662; doi:10.2196/28400	Sample recruited as frail or pre-frail (cognitive frailty)	Ab
59	Kwan RYC et al. (2024) [115]. Effects of Virtual Reality Motor-Cognitive Training for Older People With Cognitive Frailty: Multicentered Randomized Controlled Trial. J Med Internet Res. PMID 39259959; doi:10.2196/57809	Sample recruited as frail or pre-frail (cognitive frailty)	Ab
60	Köyağasıoğlu O et al. (2022) [116]. The Effects of Virtual Reality Nonphysical Mental Training on Balance Skills and Functional Near-Infrared Spectroscopy Activity in Healthy Adults. J Sport Rehabil. PMID 35104787; doi:10.1123/jsr.2021-0197	Not older adults (mean age < 60 years)	Ab
61	Lee S et al. (2013) [117]. Effectiveness of virtual reality using video gaming technology in elderly adults with diabetes mellitus. Diabetes Technol Ther. PMID 23560480; doi:10.1089/dia.2013.0050	Sample defined by a specific diagnosis (diabetes mellitus)	Ab
62	Leung KM et al. (2025) [118]. Effects of Competitive Exergaming-Based Esports on Older Adults in Hong Kong: Nonrandomized Controlled Pilot Study. JMIR Serious Games. PMID 41397691; doi:10.2196/77655	Not randomised (non-randomised or quasi-randomised allocation, or randomisation not reported)	Ab
63	Levy F et al. (2016) [119]. Fear of falling: efficacy of virtual reality associated with serious games in elderly people. Neuropsychiatr Dis Treat. PMID 27143889; doi:10.2147/NDT.S97809	Not randomised (allocation by drawing lots; authors state it cannot be regarded as a true RCT)	FT
64	Li Y et al. (2024) [120]. Impact of Virtual Reality-Based Group Activities on Activity Level and Well-Being Among Older Adults in Nursing Homes: Longitudinal Exploratory Study. JMIR Serious Games. PMID 38551635; doi:10.2196/50796	Institutionalised or residential-care sample (nursing home, long-term care, hostel, inpatient)	Ab
65	Liao YY et al. (2019) [121]. Effects of Kinect-based exergaming on frailty status and physical performance in prefrail and frail elderly: A randomized controlled trial. Sci Rep. PMID 31249332; doi:10.1038/s41598-019-45767-y	Sample recruited as frail or pre-frail	Ab
66	Lima AMN et al. (2025) [122]. Efficacy of Nintendo Switch Rehabilitation Program for Older Adults: A Quasi-Experimental Study. Games Health J. PMID 40376779; doi:10.1089/g4h.2024.0165	Institutionalised or residential-care sample (nursing home, long-term care, hostel, inpatient); Not randomised (non-randomised or quasi-randomised allocation, or randomisation not reported)	Ab
67	Litz E et al. (2021) [123]. Effects and Feasibility of a Memory Exergame Training in Older Adults: A Randomized Controlled Trial. Games Health J. PMID 34860129; doi:10.1089/g4h.2021.0032	No balance outcome (exergame task performance only)	Ab
68	Liu J et al. (2015) [124]. Comparison of Slip Training in VR Environment And on Moveable Platform. Biomed Sci Instrum. PMID 25996717	Single-session (acute) exposure rather than a training intervention (single slip-training session)	Ab
69	Lund HH et al. (2014) [125]. Effects of Short-Term Training of Community-Dwelling Elderly with Modular Interactive Tiles. Games Health J. PMID 25276497; doi:10.1089/g4h.2014.0028	No control group	Ab

No.	Report	Reason for exclusion	Basis
70	Martins AC et al. (2020) [126]. Effects of A "Modified" Otago Exercise Program on the Functional Abilities and Social Participation of Older Adults Living in the Community-The AGA@4life Model. Int J Environ Res Public Health. PMID 32075307; doi:10.3390/ijerph17041258	Not randomised (non-randomised or quasi-randomised allocation, or randomisation not reported)	Ab
71	Mascarenhas CHM et al. (2023) [127]. Analysis of Plantar Tactile Sensitivity in Older Women after Conventional Proprioceptive Training and Exergame. Int J Environ Res Public Health. PMID 36981942; doi:10.3390/ijerph20065033	No balance outcome (plantar tactile sensitivity)	Ab
72	Merriman NA et al. (2022) [128]. "CityQuest," A Custom-Designed Serious Game, Enhances Spatial Memory Performance in Older Adults. Front Aging Neurosci. PMID 35356302; doi:10.3389/fnagi.2022.806418	No balance outcome (spatial memory)	Ab
73	Mirelman A et al. (2016) [129]. Addition of a non-immersive virtual reality component to treadmill training to reduce fall risk in older adults (V-TIME): a randomised controlled trial. Lancet. PMID 27524393; doi:10.1016/S0140-6736(16)31325-3	Sample defined by a specific diagnosis (mixed sample including Parkinson disease and MCI subgroups)	Ab
74	Mohamed Zein El-AbdeenMohamed D et al. (2025) [130]. Effect of a home-based physical rehabilitation program via virtual reality on the functional outcomes of frail older adults: a quasi-experimental study. Sci Rep. PMID 39905167; doi:10.1038/s41598-025-88225-8	Sample recruited as frail or pre-frail; Not randomised (non-randomised or quasi-randomised allocation, or randomisation not reported)	Ab
75	Monteiro-Junior RS et al. (2017) [131]. Acute effects of exergames on cognitive function of institutionalized older persons: a single-blinded, randomized and controlled pilot study. Aging Clin Exp Res. PMID 27256080; doi:10.1007/s40520-016-0595-5	Institutionalised or residential-care sample (nursing home, long-term care, hostel, inpatient); Single-session (acute) exposure rather than a training intervention	Ab
76	Montero-Alía P et al. (2019) [132]. Controlled trial of balance training using a video game console in community-dwelling older adults. Age Ageing. PMID 31081504; doi:10.1093/ageing/afz047	Not randomised (quasi-randomised allocation) - Montero-Alía et al.	Ab
77	Moreira NB et al. (2021) [133]. Perceptive-Cognitive and Physical Function in Prefrail Older Adults: Exergaming Versus Traditional Multicomponent Training. Rejuvenation Res. PMID 32443963; doi:10.1089/rej.2020.2302	Sample recruited as frail or pre-frail	Ab
78	Morone G et al. (2016) [134]. Wii Fit is effective in women with bone loss condition associated with balance disorders: a randomized controlled trial. Aging Clin Exp Res. PMID 27154875; doi:10.1007/s40520-016-0578-6	Sample defined by a specific diagnosis (bone loss)	Ab
79	Morrison S et al. (2018) [135]. Supervised Balance Training and Wii Fit-Based Exercises Lower Falls Risk in Older Adults With Type 2 Diabetes. J Am Med Dir Assoc. PMID 29289541; doi:10.1016/j.jamda.2017.11.004	Sample defined by a specific diagnosis (type 2 diabetes)	Ab
80	Mugueta-Aguinaga I et al. (2019) [136]. Frailty Level Monitoring and Analysis after a Pilot Six-Week Randomized Controlled Clinical Trial Using the FRED Exergame Including Biofeedback Supervision in an Elderly Day Care Centre. Int J Environ Res Public Health. PMID 30823460; doi:10.3390/ijerph16050729	Sample recruited as frail or pre-frail	Ab
81	Nobari H et al. (2021) [137]. Effect of Virtual Reality Exercises on the Cognitive Status and Dual Motor Task Performance of the Aging Population. Int J Environ Res Public Health. PMID 34360294; doi:10.3390/ijerph18158005	Not randomised (design described as quasi-experimental); seated driving simulator	FT
82	Oesch P et al. (2017) [138]. Exergames versus self-regulated exercises with instruction leaflets to improve adherence during geriatric rehabilitation: a randomized controlled trial. BMC Geriatr. PMID 28330455; doi:10.1186/s12877-017-0467-7	Institutionalised or residential-care sample (nursing home, long-term care, hostel, inpatient) (geriatric inpatient rehabilitation)	Ab
83	Orsega-Smith E et al. (2012) [139]. Wii Fit Balance Intervention in Community-Dwelling Older Adults. Games Health J. PMID 26192060; doi:10.1089/g4h.2012.0043	Not randomised (non-randomised or quasi-randomised allocation, or randomisation not reported)	Ab
84	Parijat P et al. (2015) [140]. Effects of perturbation-based slip training using a virtual reality environment on slip-induced falls. Ann Biomed Eng. PMID 25245221; doi:10.1007/s10439-014-1128-z	Single-session (acute) exposure rather than a training intervention (single slip-training session)	Ab
85	Peng HT et al. (2020) [141]. Novel Mat Exergaming to Improve the Physical Performance, Cognitive Function, and Dual-Task Walking and Decrease the Fall Risk of Community-Dwelling Older Adults. Front Psychol. PMID 32793044; doi:10.3389/fpsyg.2020.01620	Not randomised (non-randomised or quasi-randomised allocation, or randomisation not reported)	Ab
86	Perrot A et al. (2021) [142]. Effect of Exergaming on Physical Fitness, Functional Mobility, and Cognitive Functioning in Adults With Down Syndrome. Am J Intellect Dev Disabil. PMID 33370786; doi:10.1352/1944-7558-126.1.34	Sample defined by a specific diagnosis (Down syndrome); Not older adults (mean age < 60 years)	Ab
87	Pichierri G et al. (2012) [143]. The effect of a cognitive-motor intervention on voluntary step execution under single and dual task conditions in older adults: a randomized controlled pilot study. Clin Interv Aging. PMID 22865999; doi:10.2147/CIA.S32558	Institutionalised or residential-care sample (nursing home, long-term care, hostel, inpatient) (hostels for the aged; same trial as Pichierri 2012)	Ab
88	Pichierri G et al. (2012) [144]. A cognitive-motor intervention using a dance video game to enhance foot placement accuracy and gait under dual task conditions in older adults: a randomized controlled trial. BMC Geriatr. PMID 23241332; doi:10.1186/1471-2318-12-74	Institutionalised or residential-care sample (nursing home, long-term care, hostel, inpatient) (hostels for the aged)	Ab
89	Pitta A et al. (2020) [145]. The Effects of Different Exergame Intensity Training on Walking Speed in Older Women. Games Health J. PMID 31770007; doi:10.1089/g4h.2019.0109	No non-VR comparator (VR in all arms)	Ab
90	Prasertsakul T et al. (2018) [146]. The effect of virtual reality-based balance training on motor learning and postural control in healthy adults: a randomized preliminary study. Biomed Eng Online. PMID 30227884; doi:10.1186/s12938-018-0550-0	Not older adults (mean age < 60 years)	Ab
91	Pyatova Y et al. (2026) [147]. A Visual Telerehabilitation Program in Virtual Reality for Age-Related Macular Degeneration: Randomized Feasibility and Proof-of-Concept Trial. JMIR Rehabil Assist Technol. PMID 42546222; doi:10.2196/87596	Sample defined by a specific diagnosis (macular degeneration)	Ab
92	Quatorze J et al. (2026) [148]. Exergaming for Healthy Aging: Associations with Functional Capacity, Social Participation, Self-Efficacy for Exercise, and Adherence to Inform Exergame Development. Sensors (Basel). PMID 42515497; doi:10.3390/s26144616	Not randomised (non-randomised allocation by physiotherapists)	FT
93	Ramnath U et al. (2021) [149]. Efficacy of interactive video gaming in older adults with memory complaints: A cluster-randomized exercise intervention. PLoS One. PMID 34032799; doi:10.1371/journal.pone.0252016	Institutionalised or residential-care sample (nursing home, long-term care, hostel, inpatient) (retirement homes; memory complaints)	Ab
94	Rogan S et al. (2016) [150]. Sensory-motor training targeting motor dysfunction and muscle weakness in long-term care elderly combined with motivational strategies: a single blind randomized controlled study. Eur Rev Aging Phys Act. PMID 27239241;	Institutionalised or residential-care sample (nursing home, long-term care, hostel, inpatient)	Ab

No.	Report	Reason for exclusion	Basis
	doi:10.1186/s11556-016-0164-0		
95	Sadeghi H et al. (2017) [151]. The effect of exergaming on knee proprioception in older men: A randomized controlled trial. Arch Gerontol Geriatr. PMID 27923177; doi:10.1016/j.archger.2016.11.009	No balance outcome (knee proprioception only)	Ab
96	Sadura-Sieklucka T et al. (2023) [152]. Is Balance Training Using Biofeedback Effective in the Prophylaxis of Falls in Women over the Age of 65? Brain Sci. PMID 37190594; doi:10.3390/brainsci13040629	Sample defined by a specific diagnosis (osteoarthritis)	Ab
97	Santos GOR et al. (2019) [153]. Does exercise intensity increment in exergame promote changes in strength, functional capacity and perceptual parameters in pre-frail older women? A randomized controlled trial. Exp Gerontol. PMID 30553026; doi:10.1016/j.exger.2018.12.009	Sample recruited as frail or pre-frail; No non-VR comparator (VR in all arms)	Ab
98	Saredakis D et al. (2021) [154]. The Effect of Reminiscence Therapy Using Virtual Reality on Apathy in Residential Aged Care: Multisite Nonrandomized Controlled Trial. J Med Internet Res. PMID 34542418; doi:10.2196/29210	Institutionalised or residential-care sample (nursing home, long-term care, hostel, inpatient); Not randomised (non-randomised or quasi-randomised allocation, or randomisation not reported)	Ab
99	Schwenk M et al. (2014) [155]. Interactive balance training integrating sensor-based visual feedback of movement performance: a pilot study in older adults. J Neuroeng Rehabil. PMID 25496052; doi:10.1186/1743-0003-11-164	Not described as community-dwelling (residents of a senior living community; independent or assisted living not specified)	FT
100	Schättin A et al. (2016) [156]. Adaptations of Prefrontal Brain Activity, Executive Functions, and Gait in Healthy Elderly Following Exergame and Balance Training: A Randomized-Controlled Study. Front Aging Neurosci. PMID 27932975; doi:10.3389/fnagi.2016.00278	No balance outcome (prefrontal activity, executive function and gait)	Ab
101	Schättin A et al. (2019) [157]. Effects of exergame training combined with omega-3 fatty acids on the elderly brain: a randomized double-blind placebo-controlled trial. BMC Geriatr. PMID 30866834; doi:10.1186/s12877-019-1084-4	No non-VR comparator (VR in all arms)	Ab
102	Self MA et al. (2025) [158]. Mechanism evaluation of a digitally enabled rehabilitation intervention for people in aged care and neurological rehabilitation: mediation analysis of the AMOUNT trial. Disabil Rehabil. PMID 39861985; doi:10.1080/09638288.2025.2454298	Institutionalised or residential-care sample (nursing home, long-term care, hostel, inpatient)	Ab
103	Shin D et al. (2024) [159]. A Study on the Effectiveness of VR Rehabilitation Training Content for Older Individuals with Total Knee Replacement: Pilot Study. Healthcare (Basel). PMID 39120203; doi:10.3390/healthcare12151500	Sample defined by a specific diagnosis (total knee replacement)	Ab
104	Sokołowska B et al. (2024) [160]. A machine learning approach to evaluate the impact of virtual balance/cognitive training on fall risk in older women. Front Comput Neurosci. PMID 38808222; doi:10.3389/fncom.2024.1390208	No non-VR comparator (VR in all arms)	Ab
105	Song S et al. (2026) [161]. Feasibility and Preliminary Effects of a Pressure Sensor-Based Exergame on Cognitive and Sensorimotor Frailty in Low-Income Older Adults Receiving Public Community Care Services: Pilot Randomized Controlled Trial. JMIR Aging. PMID 42497405; doi:10.2196/93467	Sample recruited as frail or pre-frail	Ab
106	Stanmore E et al. (2024) [162]. Exergames for falls prevention in sheltered homes: a feasibility study. Front Public Health. PMID 38975352; doi:10.3389/fpubh.2024.1344019	Institutionalised or residential-care sample (nursing home, long-term care, hostel, inpatient) (sheltered housing)	Ab
107	Stephan AT et al. (2024) [163]. Older Adults' Perceptions of Cognitive, Physical, and Exergame Training: A Mixed Methods Investigation of a Four-Armed Randomized Controlled Pilot Trial. Games Health J. PMID 39069966; doi:10.1089/g4h.2023.0208	No balance outcome (perceptions; mixed methods)	Ab
108	Sugiyama K et al. (2026) [164]. Feasibility and safety of mixed reality treadmill walking in older adults: a pilot and feasibility trial. Disabil Rehabil Assist Technol. PMID 41697853; doi:10.1080/17483107.2026.2626848	Single-session (acute) exposure rather than a training intervention	Ab
109	Szczepocka E et al. (2024) [165]. The Effectiveness of Virtual Reality-Based Training on Cognitive, Social, and Physical Functioning in High-Functioning Older Adults (CoSoPhy FX): 2-Arm, Parallel-Group Randomized Controlled Trial. JMIR Res Protoc. PMID 38837194; doi:10.2196/53261	No non-VR comparator (VR in all arms) (control used VR headset)	Ab
110	Szturm T et al. (2011) [166]. Effects of an interactive computer game exercise regimen on balance impairment in frail community-dwelling older adults: a randomized controlled trial. Phys Ther. PMID 21799138; doi:10.2522/ptj.20090205	Sample recruited as frail or pre-frail; geriatric day hospital	Ab
111	Taylor L et al. (2018) [167]. Exergames to Improve the Mobility of Long-Term Care Residents: A Cluster Randomized Controlled Trial. Games Health J. PMID 29265884; doi:10.1089/g4h.2017.0084	Institutionalised or residential-care sample (nursing home, long-term care, hostel, inpatient)	Ab
112	Wherry SJ et al. (2019) [168]. Feasibility of a Home-Based Balance Intervention in Middle-Aged Women Using Wii Fit Plus®. J Phys Act Health. PMID 31310993; doi:10.1123/jpah.2018-0265	Not older adults (mean age < 60 years)	Ab
113	Wilson O et al. (2024) [169]. Exploring Virtual Reality-Based Training's Effects on Balance Ability and Balance Confidence in Older Adults. Annu Int Conf IEEE Eng Med Biol Soc. PMID 40039733; doi:10.1109/EMBC53108.2024.10782002	Same study as Arnold 2024 (allocation method not reported)	FT
114	Wilson O et al. (2024) [170]. Investigating the Effects of Virtual Reality-Based Training on Balance Ability and Balance Confidence in Older Individuals. Appl Sci (Basel). PMID 40161354; doi:10.3390/app14114581	Same study as Arnold 2024 (allocation method not reported)	FT
115	Wong AKC et al. (2024) [171]. The Effect of Young People-Assisted, Individualized, Motion-Based Video Games on Physical, Cognitive, and Social Frailty Among Community-Dwelling Older Adults With Frailty: Randomized Controlled Trial. JMIR Serious Games. PMID 39622701; doi:10.2196/57352	Sample recruited as frail or pre-frail (frailty; mild neurocognitive disorder)	Ab
116	Wu YZ et al. (2019) [172]. Effects of a hybrid intervention combining exergaming and physical therapy among older adults in a long-term care facility. Geriatr Gerontol Int. PMID 30523659; doi:10.1111/ggi.13575	Institutionalised or residential-care sample (nursing home, long-term care, hostel, inpatient)	Ab
117	Yeşilyaprak SS et al. (2016) [173]. Comparison of the effects of virtual reality-based balance exercises and conventional exercises on	Institutionalised or residential-care sample (nursing home, long-term care, hostel,	Ab

No.	Report	Reason for exclusion	Basis
	balance and fall risk in older adults living in nursing homes in Turkey. Physiother Theory Pract. PMID 27049879; doi:10.3109/09593985.2015.1138009	inpatient)	
118	Yousefi Babadi S et al. (2021) [174]. Effects of virtual reality versus conventional balance training on balance of the elderly. Exp Gerontol. PMID 34311059; doi:10.1016/j.exger.2021.111498	Institutionalised or residential-care sample (nursing home, long-term care, hostel, inpatient)	Ab
119	Zahedian-Nasab N et al. (2021) [175]. Effect of virtual reality exercises on balance and fall in elderly people with fall risk: a randomized controlled trial. BMC Geriatr. PMID 34563120; doi:10.1186/s12877-021-02462-w	Institutionalised or residential-care sample (nursing home, long-term care, hostel, inpatient)	Ab
120	Zukowski LA et al. (2022) [176]. Acute effects of virtual reality treadmill training on gait and cognition in older adults: A randomized controlled trial. PLoS One. PMID 36322594; doi:10.1371/journal.pone.0276989	Single-session (acute) exposure rather than a training intervention	Ab
121	de Bruin ED et al. (2020) [177]. Combining Stochastic Resonance Vibration With Exergaming for Motor-Cognitive Training in Long-Term Care; A Sham-Control Randomized Controlled Pilot Trial. Front Med (Lausanne). PMID 33330519; doi:10.3389/fmed.2020.507155	Institutionalised or residential-care sample (nursing home, long-term care, hostel, inpatient)	Ab
122	de Rond V et al. (2023) [178]. Neural correlates of weight-shift training in older adults: a randomized controlled study. Sci Rep. PMID 37949995; doi:10.1038/s41598-023-46645-4	Single-session (acute) exposure rather than a training intervention	Ab

Supplementary Table S3. PRISMA 2020 checklist

Location refers to the revised manuscript (section, table or figure).

Section	Item	Topic	Checklist item	Location in manuscript
TITLE	1	Title	Identify the report as a systematic review.	Title
ABSTRACT	2	Abstract	See the PRISMA 2020 for Abstracts checklist.	Abstract
INTRODUCTION	3	Rationale	Describe the rationale for the review in the context of existing knowledge.	Section 1, paragraphs 1–3 (including relationship to the earlier review [14])
	4	Objectives	Provide an explicit statement of the objective(s) or question(s) the review addresses.	Section 1, final paragraph; Table 1
METHODS	5	Eligibility criteria	Specify the inclusion and exclusion criteria and how studies were grouped for the syntheses.	Section 2.2; Table 1; Section 2.5 (grouping)
	6	Information sources	Specify all databases and other sources searched and the date when each was last searched.	Section 2.1; Table S1
	7	Search strategy	Present the full search strategies for all databases, including any filters and limits used.	Section 2.1; Table S1
	8	Selection process	Specify the methods used to decide whether a study met the inclusion criteria, including how many reviewers screened each record.	Sections 2.1 and 3.1
	9	Data collection process	Specify the methods used to collect data from reports, including how many reviewers collected data.	Section 2.4
	10a	Data items	List and define all outcomes for which data were sought.	Sections 2.4 and 2.5 (4 balance domains; outcome hierarchy)
	10b	Data items	List and define all other variables for which data were sought.	Section 2.4
	11	Study risk of bias assessment	Specify the methods used to assess risk of bias in the included studies.	Section 2.3; Table S4
	12	Effect measures	Specify for each outcome the effect measure(s) used in the synthesis or presentation of results.	Section 2.5 (direction of the between-group effect for 1 prespecified outcome per trial and domain; statistical clarity recorded separately)
	13a	Synthesis methods	Describe the processes used to decide which studies were eligible for each synthesis.	Section 2.5 (comparator type × outcome domain; add-on designs identified); Table 3
	13b	Synthesis methods	Describe any methods required to prepare the data for presentation or synthesis.	Section 2.5 (outcome hierarchy; end-of-intervention difference or difference in change)
	13c	Synthesis methods	Describe any methods used to tabulate or visually display results.	Section 2.5; Tables 2–4 (effect direction plot, summary-of-findings table)
	13d	Synthesis methods	Describe any methods used to synthesise results and provide a rationale for the choices made.	Section 2.5 (SWiM; reasons meta-analysis not feasible)
	13e	Synthesis methods	Describe any methods used to explore possible causes of heterogeneity.	Section 2.5 (stratification by comparator type, add-on design); Section 4 (platform type, risk of bias)
	13f	Synthesis methods	Describe any sensitivity analyses conducted.	Section 2.2 (restriction to trials meeting the registered age criterion)
	14	Reporting bias assessment	Describe any methods used to assess risk of bias due to missing results.	Section 2.7 (GRADE publication-bias domain); formal tests not applicable (no pooled effect estimates), Section 4
	15	Certainty assessment	Describe any methods used to assess certainty in the body of evidence.	Section 2.7
RESULTS	16a	Study selection	Describe the results of the search and selection process, ideally using a flow diagram.	Section 3.1; Figure 1
	16b	Study selection	Cite studies that might appear to meet the inclusion criteria but were excluded, and explain why.	Section 3.1; Table S2 (updated search); Table S5 (awaiting classification)
	17	Study characteristics	Cite each included study and present its characteristics.	Section 3.3; Table 2
	18	Risk of bias in studies	Present assessments of risk of bias for each included study.	Section 3.2; Figures 2 and 3; Table S4
	19	Results of individual studies	For all outcomes, present for each study summary statistics and an effect estimate where available.	Table 2 (between-group results); Table 3

Section	Item	Topic	Checklist item	Location in manuscript
	20a	Results of syntheses	For each synthesis, briefly summarise the characteristics and risk of bias among contributing studies.	Section 3.5; Table 3
	20b	Results of syntheses	Present results of all statistical syntheses conducted.	Section 3.5; Table 4
	20c	Results of syntheses	Present results of all investigations of possible causes of heterogeneity.	Section 3.5 (by comparator type and risk of bias); Section 4
	20d	Results of syntheses	Present results of all sensitivity analyses conducted.	Section 3.5, final paragraph; Table 4 footnote
	21	Reporting biases	Present assessments of risk of bias due to missing results.	Section 3.7; Table 4 (publication bias not downgraded); Section 4 (limitation)
	22	Certainty of evidence	Present assessments of certainty in the body of evidence for each outcome assessed.	Section 3.7; Table 4
DISCUSSION	23a	Discussion	Provide a general interpretation of the results in the context of other evidence.	Section 4, paragraphs 1–3
	23b	Discussion	Discuss any limitations of the evidence included in the review.	Section 4 (risk of bias paragraph)
	23c	Discussion	Discuss any limitations of the review processes used.	Section 4 (limitations paragraph)
	23d	Discussion	Discuss implications of the results for practice, policy, and future research.	Section 4; Section 5
OTHER INFORMATION	24a	Registration and protocol	Provide registration information for the review.	Section 2 (PROSPERO CRD42023451977)
	24b	Registration and protocol	Indicate where the review protocol can be accessed.	Section 2 (PROSPERO record)
	24c	Registration and protocol	Describe and explain any amendments to information provided at registration or in the protocol.	Section 2.8
	25	Support	Describe sources of financial or non-financial support for the review.	Funding statement
	26	Competing interests	Declare any competing interests of review authors.	Conflicts of Interest statement
	27	Availability of data, code and other materials	Report which of the following are publicly available and where they can be found.	Data Availability Statement; Tables 2–4; Supplementary Tables S1, S2 and S4

Supplementary Table S4. RoB 2 judgements with signalling-question responses and supporting information for each trial

Assessed result: between-group comparison of the primary balance outcome at the end of the intervention; effect of assignment to the intervention. L, low risk; SC, some concerns; H, high risk; Y, yes; PY, probably yes; PN, probably no; N, no; NI, no information.

Trial	D1 Randomisation	D2 Deviations	D3 Missing data	D4 Measurement	D5 Reported result	Overall
Bateni 2012 [28]	Some concerns: 1.1 PY (random allocation stated); 1.2 NI (allocation concealment not reported); 1.3 N (no baseline imbalance suggesting a problem)	Some concerns: 2.1 Y; 2.2 Y; 2.3 NI; 2.6 N/NI (no ITT analysis reported); 2.7 PN/NI	Low: 3.1 Y/PY (outcome data for all or nearly all participants)	Low: 4.1 N; 4.2 N; 4.3 N/PN (assessors blinded) or 4.4 N (outcome not open to assessor influence)	Some concerns: 5.1 NI (no registered protocol or analysis plan identified); 5.2 PN; 5.3 PN	Some concerns
Rendon et al. 2012 [49]	Some concerns: 1.1 PY (random allocation stated); 1.2 NI (allocation concealment not reported); 1.3 N (no baseline imbalance suggesting a problem)	Low: 2.1 Y; 2.2 Y; 2.3 N; 2.6 Y (analysed as randomised); 2.7 NA	Low: 3.1 Y/PY (outcome data for all or nearly all participants)	Low: 4.1 N; 4.2 N; 4.3 N/PN (assessors blinded) or 4.4 N (outcome not open to assessor influence)	Some concerns: 5.1 NI (no registered protocol or analysis plan identified); 5.2 PN; 5.3 PN	Some concerns
Franco et al. 2012 [27]	Some concerns: 1.1 PY (random allocation stated); 1.2 NI (allocation concealment not reported); 1.3 N (no baseline imbalance suggesting a problem)	Low: 2.1 Y; 2.2 Y; 2.3 N; 2.6 Y (analysed as randomised); 2.7 NA	Low: 3.1 Y/PY (outcome data for all or nearly all participants)	Low: 4.1 N; 4.2 N; 4.3 N/PN (assessors blinded) or 4.4 N (outcome not open to assessor influence)	Some concerns: 5.1 NI (no registered protocol or analysis plan identified); 5.2 PN; 5.3 PN	Some concerns
Singh et al. 2012/2013 [17,18]	Some concerns: 1.1 PY (random allocation stated); 1.2 NI (allocation concealment not reported); 1.3 N (no baseline imbalance suggesting a problem)	Some concerns: 2.1 Y; 2.2 Y; 2.3 NI; 2.6 N/NI (no ITT analysis reported); 2.7 PN/NI	Low: 3.1 Y/PY (outcome data for all or nearly all participants)	Low: 4.1 N; 4.2 N; 4.3 N/PN (assessors blinded) or 4.4 N (outcome not open to assessor influence)	Some concerns: 5.1 NI (no registered protocol or analysis plan identified); 5.2 PN; 5.3 PN	Some concerns
Bieryla & Dold 2013 [29]	Some concerns: 1.1 PY (random allocation stated); 1.2 NI (allocation concealment not reported); 1.3 N (no baseline imbalance suggesting a problem)	Some concerns: 2.1 Y; 2.2 Y; 2.3 NI; 2.6 N/NI (no ITT analysis reported); 2.7 PN/NI	High: 3.1 N (9 of 12 analysed; 25% missing); 3.2 N; 3.3 PY; 3.4 PY	High: 4.1 N; 4.2 N; 4.3 Y (assessors not blinded); 4.4 Y; 4.5 PY	Some concerns: 5.1 NI (no registered protocol or analysis plan identified); 5.2 PN; 5.3 PN	High
Jorgensen et al. 2013 [7]	Low: 1.1 Y (computer-generated random numbers in permuted blocks, stratified by sex); 1.2 Y — "All participant allocation procedures were handled by the laboratory's chief nurse who was not involved in any other parts of the study."; 1.3 N (no baseline differences)	Some concerns: 2.1 Y; 2.2 Y; 2.3 NI; 2.6 N/NI (no ITT analysis reported); 2.7 PN/NI	Low: 3.1 PY (intention-to-treat analysis of all 58 randomised, last observation carried forward; sensitivity analysis excluding 5 non-starters did not alter findings)	Low: 4.1 N; 4.2 N; 4.3 N/PN (assessors blinded) or 4.4 N (outcome not open to assessor influence)	Low: 5.1 Y (registered protocol NCT01371253); 5.2 N; 5.3 N	Some concerns
Kim et al. 2013 [54]	Some concerns: 1.1 PY (random allocation stated); 1.2 NI (allocation concealment not reported); 1.3 N (no baseline imbalance suggesting a problem)	Some concerns: 2.1 Y; 2.2 Y; 2.3 NI; 2.6 N/NI (no ITT analysis reported); 2.7 PN/NI	High: 3.1 N; 3.2 N; 3.3 PY; 3.4 PY	Low: 4.1 N; 4.2 N; 4.3 N/PN (assessors blinded) or 4.4 N (outcome not open to assessor influence)	Some concerns: 5.1 NI (no registered protocol or analysis plan identified); 5.2 PN; 5.3 PN	High
Schoene et al. 2013 [34]	Low: 1.1 Y (computer-generated list); 1.2 PY (list prepared by staff not involved in the study; all participants randomised at one time point); 1.3 N	Some concerns: 2.1 Y; 2.2 Y; 2.3 NI; 2.6 N (completers analysed, 32 of 37); 2.7 PN	Some concerns: 3.1 N (86% analysed); 3.2 N; 3.3 PY; 3.4 PN (reasons reported, balanced losses)	Low: 4.1 N; 4.2 N; 4.3 N (assessors blinded)	Some concerns: 5.1 NI (ACTRN12611001081909; registration date relative to enrolment not established); 5.2 PN; 5.3 PN	Some concerns
Eggenberger et al. 2015 [48]	Some concerns: 1.1 PY (random allocation stated); 1.2 NI (allocation concealment not reported); 1.3 N (no baseline imbalance suggesting a problem)	Some concerns: 2.1 Y; 2.2 Y; 2.3 NI; 2.6 N/NI (no ITT analysis reported); 2.7 PN/NI	High: 3.1 N (71 of 89 analysed); 3.2 N; 3.3 PY; 3.4 PY	High: 4.1 N; 4.2 N; 4.3 Y (assessors not blinded); 4.4 Y; 4.5 PY	Low: 5.1 Y (registered protocol); 5.2 N; 5.3 N	High
Gschwind et al. 2015 [35]	Some concerns: 1.1 Y; 1.2 NI (allocation concealment not described); 1.3 N	Low: 2.1 Y; 2.2 Y; 2.3 N; 2.6 Y (re-assessed participants analysed as randomised)	Some concerns: 3.1 N (136 of 153 re-assessed; losses per arm not reported); 3.2 N; 3.3 PY; 3.4 PN	Low: 4.1 N; 4.2 N; 4.3 N (assessors blinded)	Some concerns: 5.1 PN (ACTRN12614000096651, protocol published; repeated-measures ANOVA reported instead of the planned ANCOVA, and the planned falls analysis was not reported); 5.2	Some concerns

Trial	D1 Randomisation	D2 Deviations	D3 Missing data	D4 Measurement	D5 Reported result	Overall
					PN; 5.3 PN	
Lee et al. 2015 [50]	Some concerns: 1.1 PY (random allocation stated); 1.2 NI (allocation concealment not reported); 1.3 N (no baseline imbalance suggesting a problem)	Low: 2.1 Y; 2.2 Y; 2.3 N; 2.6 Y (analysed as randomised); 2.7 NA	Low: 3.1 Y/PY (outcome data for all or nearly all participants)	Low: 4.1 N; 4.2 N; 4.3 N/PN (assessors blinded) or 4.4 N (outcome not open to assessor influence)	Some concerns: 5.1 NI (no registered protocol or analysis plan identified); 5.2 PN; 5.3 PN	Some concerns
Whyatt et al. 2015 [52]	Some concerns: 1.1 PY (random allocation stated); 1.2 NI (allocation concealment not reported); 1.3 N (no baseline imbalance suggesting a problem)	Some concerns: 2.1 Y; 2.2 Y; 2.3 NI; 2.6 N/NI (no ITT analysis reported); 2.7 PN/NI	Low: 3.1 Y/PY (outcome data for all or nearly all participants)	Low: 4.1 N; 4.2 N; 4.3 N/PN (assessors blinded) or 4.4 N (outcome not open to assessor influence)	Some concerns: 5.1 NI (no registered protocol or analysis plan identified); 5.2 PN; 5.3 PN	Some concerns
Kwok & Pua 2016 [47]	Low: 1.1 Y; 1.2 Y (consecutively numbered sealed envelopes); 1.3 N	Some concerns: 2.1 Y; 2.2 Y; 2.3 NI; 2.6 N/NI (no ITT analysis reported); 2.7 PN/NI	Low: 3.1 Y (no losses to follow-up; 40/40 per arm)	Low: 4.1 N; 4.2 N; 4.3 N/PN (assessors blinded) or 4.4 N (outcome not open to assessor influence)	Some concerns: 5.1 NI (no registered protocol or analysis plan identified); 5.2 PN; 5.3 PN	Some concerns
Park & Yim 2016 [51]	Some concerns: 1.1 PY (random allocation stated); 1.2 NI (allocation concealment not reported); 1.3 N (no baseline imbalance suggesting a problem)	Low: 2.1 Y; 2.2 Y; 2.3 N; 2.6 Y (analysed as randomised); 2.7 NA	Low: 3.1 Y/PY (outcome data for all or nearly all participants)	Low: 4.1 N; 4.2 N; 4.3 N/PN (assessors blinded) or 4.4 N (outcome not open to assessor influence)	Some concerns: 5.1 NI (no registered protocol or analysis plan identified); 5.2 PN; 5.3 PN	Some concerns
Ordnung et al. 2017 [36]	Some concerns: 1.1 PY (random allocation stated); 1.2 NI; 1.3 N	Low: 2.1 Y; 2.2 Y; 2.3 N; 2.6 PN; 2.7 N (1 of 30 not analysed)	Low: 3.1 Y (29 of 30 analysed)	Low: 4.1 N; 4.2 N; 4.3 NI; 4.4 PN (instrumented centre-of-pressure measure)	Some concerns: 5.1 N (no registration); 5.2 PN; 5.3 PN (single-leg balance measured but not reported)	Some concerns
Rodrigues et al. 2018 [55]	Some concerns: 1.1 PY (random allocation stated); 1.2 NI (allocation concealment not reported); 1.3 N (no baseline imbalance suggesting a problem)	Some concerns: 2.1 Y; 2.2 Y; 2.3 NI; 2.6 N/NI (no ITT analysis reported); 2.7 PN/NI	High: 3.1 N; 3.2 N; 3.3 PY; 3.4 PY	High: 4.1 N; 4.2 N; 4.3 Y (assessors not blinded); 4.4 Y; 4.5 PY	Some concerns: 5.1 NI (no registered protocol or analysis plan identified); 5.2 PN; 5.3 PN	High
Adcock et al. 2019 [37]	High: 1.1 PY; 1.2 NI; 1.3 Y (age and fear of falling differed between arms at baseline, P = 0.005 for age)	Some concerns: 2.1 Y; 2.2 Y; 2.3 NI; 2.6 N (per-protocol analysis, 31 of 37); 2.7 PN	Some concerns: 3.1 N (84% analysed); 3.2 N; 3.3 PY; 3.4 PN	High: 4.1 N; 4.2 N; 4.3 Y (assessors not blinded); 4.4 PY (performance test); 4.5 PY	Some concerns: 5.1 N (NCT03676452 registered after recruitment had started); 5.2 PN; 5.3 PN	High
Yu et al. 2020 [22]	Some concerns: 1.1 PY; 1.2 NI; 1.3 N	Some concerns: 2.1 Y; 2.2 Y; 2.3 NI; 2.6 N (completers analysed); 2.7 PN	Some concerns: 3.1 N (40 of 43 for the up-and-go test; 31 of 43 for the static balance test); 3.2 N; 3.3 PY; 3.4 PN	Some concerns: 4.1 N; 4.2 N; 4.3 NI; 4.4 PY (timed performance test); 4.5 PN	Some concerns: 5.1 N (no registration); 5.2 PN; 5.3 PN	Some concerns
Barsasella et al. 2021 [23]	Low: 1.1 Y; 1.2 Y; 1.3 N	Low: 2.1 Y; 2.2 Y; 2.3 N; 2.6 Y (analysed as randomised); 2.7 NA	Low: 3.1 Y/PY (outcome data for all or nearly all participants)	Low: 4.1 N; 4.2 N; 4.3 N/PN (assessors blinded) or 4.4 N (outcome not open to assessor influence)	Low: 5.1 Y (registered protocol NCT04166747); 5.2 N; 5.3 N	Low
Bagheri et al. 2021/Khanmohammadi et al. 2022 [19,20]	Low: 1.1 Y; 1.2 Y; 1.3 N	Some concerns: 2.1 Y; 2.2 Y; 2.3 NI; 2.6 N/NI (no ITT analysis reported); 2.7 PN/NI	High: 3.1 N; 3.2 N; 3.3 PY; 3.4 PY	Low: 4.1 N; 4.2 N; 4.3 N/PN (assessors blinded) or 4.4 N (outcome not open to assessor influence)	Low: 5.1 Y (registered protocol); 5.2 N; 5.3 N	High
Kim et al. 2021 [21]	Some concerns: 1.1 PY (random allocation stated); 1.2 NI (allocation concealment not reported); 1.3 N (no baseline imbalance suggesting a problem)	Low: 2.1 Y; 2.2 Y; 2.3 N; 2.6 Y (analysed as randomised); 2.7 NA	Low: 3.1 Y (20/20 analysed)	High: 4.1 N; 4.2 N; 4.3 Y (assessors not blinded); 4.4 Y; 4.5 PY	Some concerns: 5.1 NI (no registered protocol or analysis plan identified); 5.2 PN; 5.3 PN	High
Lee 2021 [38]	Some concerns: 1.1 PY; 1.2 NI; 1.3 N	Low: 2.1 Y; 2.2 Y; 2.3 N; 2.6 Y (no losses)	Low: 3.1 Y (56 of 56 analysed)	Low: 4.1 N; 4.2 N; 4.3 N (assessors blinded)	Some concerns: 5.1 N (no registration); 5.2 PN; 5.3 PN	Some concerns
Benitez-Lugo et al. 2022 [39]	Low: 1.1 NI (sequence generation not described); 1.2 Y (sealed opaque envelopes); 1.3 N	Low: 2.1 Y; 2.2 Y; 2.3 N; 2.6 PN; 2.7 N (2 of 48 not analysed)	Low: 3.1 Y (46 of 48 analysed)	Low: 4.1 N; 4.2 N; 4.3 N (assessors blinded)	Some concerns: 5.1 NI (NCT04615897; registration date relative to enrolment not reported); 5.2 PN; 5.3 PN (one-sided tests reported)	Some concerns
Zak et al. 2022 [31]	High: 1.1 PY; 1.2 NI; 1.3 Y (large baseline	Low: 2.1 Y; 2.2 Y; 2.3 N; 2.6 Y (no	Low: 3.1 Y (60 of 60 analysed)	Some concerns: 4.1 N; 4.2 N; 4.3	Some concerns: 5.1 N	High

Trial	D1 Randomisation	D2 Deviations	D3 Missing data	D4 Measurement	D5 Reported result	Overall
	differences in single-leg stance and POMA, P < 0.001)	losses)		NI; 4.4 PY (timed performance test); 4.5 PN	(ACTRN12621000719831 registered after ethics approval and enrolment); 5.2 PN; 5.3 PN	
Zhao et al. 2022 [24]	Some concerns: 1.1 Y (computer randomisation); 1.2 NI; 1.3 N	Some concerns: 2.1 Y; 2.2 Y; 2.3 NI; 2.6 N; 2.7 PY	High: 3.1 N (38 of 48 analysed; 8 losses in the control arm vs 2 in the VR arm); 3.2 N; 3.3 PY; 3.4 PY	Low: 4.1 N; 4.2 N; 4.3 NI; 4.4 PN (instrumented sway measure)	Some concerns: 5.1 N (no registration); 5.2 PN; 5.3 PN	High
Gerards et al. 2023 [46]	Low: 1.1 Y; 1.2 Y — "Allocation was concealed by using sequentially numbered, sealed opaque envelopes."; 1.3 N	Low: 2.1 Y; 2.2 Y; 2.3 N; 2.6 Y (analysed as randomised); 2.7 NA	Low: 3.1 PY (37/39 and 41/43 analysed; losses unrelated to outcome)	Low: 4.1 N; 4.2 N; 4.3 N/PN (assessors blinded) or 4.4 N (outcome not open to assessor influence)	Low: 5.1 Y (registered protocol); 5.2 N; 5.3 N	Low
Carcelén-Fraile et al. 2024 [42]	Low: 1.1 Y (computer-generated sequence); 1.2 Y (sealed opaque envelopes); 1.3 N	Low: 2.1 Y; 2.2 Y; 2.3 N; 2.6 PN; 2.7 N (3 of 76 not analysed)	Low: 3.1 Y (73 of 76 analysed)	Low: 4.1 N; 4.2 N; 4.3 N (assessor independent of allocation)	Some concerns: 5.1 NI (NCT06142812; registration date not reported); 5.2 PN; 5.3 PN	Some concerns
Hashim et al. 2024 [53]	Some concerns: 1.1 PY (random allocation stated); 1.2 NI (allocation concealment not reported); 1.3 N (no baseline imbalance suggesting a problem)	Some concerns: 2.1 Y; 2.2 Y; 2.3 NI; 2.6 N/NI (no ITT analysis reported); 2.7 PN/NI	Low: 3.1 Y/PY (outcome data for all or nearly all participants)	Low: 4.1 N; 4.2 N; 4.3 N/PN (assessors blinded) or 4.4 N (outcome not open to assessor influence)	Some concerns: 5.1 NI (no registered protocol or analysis plan identified); 5.2 PN; 5.3 PN	Some concerns
Sturnieks et al. 2024 [40]	Low: 1.1 Y; 1.2 Y (in-house web-based randomisation; code released only to non-blinded staff); 1.3 N	Low: 2.1 Y; 2.2 Y; 2.3 N; 2.6 Y (intention-to-treat)	Low: 3.1 Y (falls analysed in all 769 randomised)	Low: 4.1 N; 4.2 N; 4.3 PN (falls recorded with monthly calendars; outcome data handled by staff blinded to allocation); 4.4 PN	Low: 5.1 Y (ACTRN12616001325493 registered before recruitment; published protocol and statistical analysis plan); 5.2 N; 5.3 N	Low
Zak et al. 2024 [41]	Some concerns: 1.1 Y (computer-generated sequence); 1.2 NI; 1.3 N	Low: 2.1 Y; 2.2 Y; 2.3 N; 2.6 Y (no losses)	Low: 3.1 Y (80 of 80 analysed)	Some concerns: 4.1 N; 4.2 N; 4.3 NI; 4.4 PY (performance test); 4.5 PN	Some concerns: 5.1 N (ACTRN12621000719831 registered after ethics approval; no primary outcome named); 5.2 PN; 5.3 PN	Some concerns
Parmak et al. 2025 [43]	Some concerns: 1.1 PY; 1.2 NI; 1.3 N	Low: 2.1 Y; 2.2 Y; 2.3 N; 2.6 Y (no losses)	Low: 3.1 Y (44 of 44 analysed)	High: 4.1 N; 4.2 N; 4.3 Y (the physiotherapist who delivered the VR training performed the assessments); 4.4 PY; 4.5 PY	Some concerns: 5.1 N (NCT07007026 registered after the trial); 5.2 PN; 5.3 PN	High
Huang et al. 2026 [44]	Low: 1.1 Y (computer-generated sequence by an independent statistician); 1.2 Y (sequentially numbered, sealed opaque envelopes); 1.3 PN	Low: 2.1 Y; 2.2 Y; 2.3 N; 2.6 Y (no losses)	Low: 3.1 Y (40 of 40 analysed)	Low: 4.1 N; 4.2 N; 4.3 Y (open-label); 4.4 PN (primary outcome recorded by the force platform)	Some concerns: 5.1 N (NCT05779189 registered after the trial period); 5.2 PN; 5.3 PN	Some concerns
Kim et al. 2026 [45]	Low: 1.1 Y (random number table); 1.2 PY (sequence concealed until assignment); 1.3 N	Low: 2.1 Y; 2.2 Y; 2.3 N; 2.6 PN; 2.7 N (3 of 60 not analysed)	Low: 3.1 Y (57 of 60 analysed)	Low: 4.1 N; 4.2 N; 4.3 N (assessors blinded)	Some concerns: 5.1 N (UMIN000055778 registered after recruitment had started); 5.2 PN (primary outcome stated inconsistently between sections); 5.3 PN	Some concerns

Changes from the previous version (18 trials from the original searches): domain 1 revised from low to some concerns in 13 trials (allocation concealment not reported); domain 5 revised from low to some concerns in 13 trials (no protocol or analysis plan available); domain 3 for Kwok and Pua [47] revised from high to low (no losses to follow-up). Rows in red: 15 trials identified in the updated PubMed search (8 October 2026) and assessed for this revision. For these trials, domain 4 was judged to raise some concerns when assessor blinding was not reported for a performance-based outcome, and domain 5 was judged low only when the trial had been registered before enrolment with outcomes and analyses consistent with the report.

Supplementary Table S5. Studies awaiting classification identified in the updated PubMed search

Reports judged potentially eligible in the updated search (8 October 2026) that could not be obtained in full text, extracted and assessed for risk of bias within the revision period. The 15 reports confirmed as eligible in full text have been incorporated in the review and are not listed here. Status: Potentially eligible (Ab) = criteria met on the abstract, full text not yet assessed; Unclear = abstract insufficient (e.g., allocation method not reported); Companion = additional report of another listed trial. These reports were not incorporated in the present synthesis and will be assessed in an update of the review.

No.	Report	Status	Notes
1	Abd El-Kafy EM et al. (2024) [179]. Motion tracking virtual reality technology in improving gait in the elderly: A randomized controlled trial. Clin Rehabil. PMID 38130064; doi:10.1177/02692155231217468	Potentially eligible (Ab)	C-Mill VR treadmill
2	AlQahtani M (2024) [180]. Impact of Wii Fit Training (WFT) and Reactive Balance Training (RBT) for in Elder Age Population: A Comparative Analysis. J Pharm Bioallied Sci. PMID 38595384; doi:10.4103/jpbs.jpbs_712_23	Potentially eligible (Ab)	
3	Bacha JMR et al. (2018) [181]. Effects of Kinect Adventures Games Versus Conventional Physical Therapy on Postural Control in Elderly People: A Randomized Controlled Trial. Games Health J. PMID 29239677; doi:10.1089/g4h.2017.0065	Potentially eligible (Ab)	
4	Bieryla KA (2016) [182]. Xbox Kinect training to improve clinical measures of balance in older adults: a pilot study. Aging Clin Exp Res. PMID 26386865; doi:10.1007/s40520-015-0452-y	Potentially eligible (Ab)	
5	Campelo AM et al. (2023) [183]. Physical and Affective Physical Literacy Domains Improved After a Six-Week Exergame Exercise Program in Older Adults: A Randomized Controlled Clinical Trial. Games Health J. PMID 37311178; doi:10.1089/g4h.2022.0212	Potentially eligible (Ab)	
6	Chan WLS et al. (2024) [184]. Feasibility, safety, and effects of a Nintendo Ring Fit Adventure™ balance and strengthening exercise program in community-dwelling older adults with a history of falls: A feasibility randomized controlled trial. Geriatr Gerontol Int. PMID 38088479; doi:10.1111/ggi.14771	Potentially eligible (Ab)	Community fallers
7	Chang CL et al. (2026) [185]. The Effects of Eight-Week Traditional Aerobic Exercise and Exergaming on Dual-Task Performance and Prefrontal Cortex Activation in Older Adults. Med Sci Sports Exerc. PMID 42384848; doi:10.1249/MSS.0000000000004067	Potentially eligible (Ab)	Mean age 64
8	Chuang IC et al. (2025) [186]. Effects of immersive leisure-based virtual reality cognitive training on cognitive and physical function in community-based older adults: A randomized controlled trial. Digit Health. PMID 40171148; doi:10.1177/20552076251328491	Potentially eligible (Ab)	Cluster RCT
9	Duque G et al. (2013) [187]. Effects of balance training using a virtual-reality system in older fallers. Clin Interv Aging. PMID 23467506; doi:10.2147/CIA.S41453	Potentially eligible (Ab)	
10	Evangelista de Lima B et al. (2021) [188]. Effects of Xbox Kinect exercise training on sleep quality, anxiety and functional capacity in older adults. J Bodyw Mov Ther. PMID 34776152; doi:10.1016/j.jbmt.2021.07.029	Potentially eligible (Ab)	
11	Fidan O et al. (2025) [189]. Effects of group-based virtual reality training on activities of daily living and functional outcomes in older adults: a randomised control trial. Disabil Rehabil Assist Technol. PMID 39570714; doi:10.1080/17483107.2024.2431051	Potentially eligible (Ab)	
12	Gallardo-Meza C et al. (2022) [190]. Effects of 4 Weeks of Active Exergames Training on Muscular Fitness in Elderly Women. J Strength Cond Res. PMID 32483059; doi:10.1519/JSC.0000000000003560	Potentially eligible (Ab)	
13	Ghous M et al. (2024) [191]. Comparison of Nonimmersive Virtual Reality and Task-Oriented Circuit Training on Gait, Balance, and Cognition Among Elderly Population: A Single-Blind Randomized Control Trial. Games Health J. PMID 38512706; doi:10.1089/g4h.2022.0205	Potentially eligible (Ab)	
14	Gonçalves A et al. (2021) [192]. The Benefits of Custom Exergames for Fitness, Balance, and Health-Related Quality of Life: A Randomized Controlled Trial with Community-Dwelling Older Adults. Games Health J. PMID 34370609; doi:10.1089/g4h.2020.0092	Potentially eligible (Ab)	Add-on design
15	Guede-Rojas F et al. (2023) [193]. Effects of Exergames and Conventional Physical Therapy on Functional Physical Performance in Older Adults: A Randomized Controlled Trial. Games Health J. PMID 37585611; doi:10.1089/g4h.2022.0194	Potentially eligible (Ab)	Add-on design
16	Hou HY et al. (2022) [194]. Effects of exergame and video game training on cognitive and physical function in older adults: A randomized controlled trial. Appl Ergon. PMID 35066398; doi:10.1016/j.apergo.2022.103690	Potentially eligible (Ab)	Comparators: sedentary video game and no training
17	Jung DI et al. (2015) [195]. Kinematic effect of Nintendo Wii(TM) sports program exercise on obstacle gait in elderly women with falling risk. J Phys Ther Sci. PMID 26157228; doi:10.1589/jpts.27.1397	Potentially eligible (Ab)	Older women at risk of falls; three arms
18	Karahan AY et al. (2015) [196]. Effects of Exergames on Balance, Functional Mobility, and Quality of Life of Geriatrics Versus Home Exercise Programme: Randomized Controlled Study. Cent Eur J Public Health. PMID 26849537; doi:10.21101/cejph.a4081	Potentially eligible (Ab)	
19	Khushnood K et al. (2021) [197]. Effects of Wii Fit exer-gaming on balance and gait in elderly population: A randomized control trial. J Pak Med Assoc. PMID 33819217; doi:10.47391/JPMA.565	Potentially eligible (Ab)	
20	Lima Rebêlo F et al. (2021) [198]. Immersive virtual reality is effective in the rehabilitation of older adults with balance disorders: A randomized clinical trial. Exp Gerontol. PMID 33744393; doi:10.1016/j.exger.2021.111308	Potentially eligible (Ab)	Older adults with balance disorders

No.	Report	Status	Notes
21	Morat M et al. (2019) [199]. Effects of stepping exergames under stable versus unstable conditions on balance and strength in healthy community-dwelling older adults: A three-armed randomized controlled trial. Exp Gerontol. PMID 31513877; doi:10.1016/j.exger.2019.110719	Potentially eligible (Ab)	
22	Padala KP et al. (2017) [200]. Efficacy of Wii-Fit on Static and Dynamic Balance in Community Dwelling Older Veterans: A Randomized Controlled Pilot Trial. J Aging Res. PMID 28261500; doi:10.1155/2017/4653635	Potentially eligible (Ab)	Community-dwelling veterans
23	Park EC et al. (2015) [201]. The effects of virtual reality game exercise on balance and gait of the elderly. J Phys Ther Sci. PMID 25995578; doi:10.1589/jpts.27.1157	Potentially eligible (Ab)	
24	Pluchino A et al. (2012) [202]. Pilot study comparing changes in postural control after training using a video game balance board program and 2 standard activity-based balance intervention programs. Arch Phys Med Rehabil. PMID 22414490; doi:10.1016/j.apmr.2012.01.023	Potentially eligible (Ab)	
25	Sadeghi H et al. (2021) [203]. Effects of 8 Weeks of Balance Training, Virtual Reality Training, and Combined Exercise on Lower Limb Muscle Strength, Balance, and Functional Mobility Among Older Men: A Randomized Controlled Trial. Sports Health. PMID 33583253; doi:10.1177/1941738120986803	Potentially eligible (Ab)	
26	Sato K et al. (2015) [204]. Improving Walking, Muscle Strength, and Balance in the Elderly with an Exergame Using Kinect: A Randomized Controlled Trial. Games Health J. PMID 26182059; doi:10.1089/g4h.2014.0057	Potentially eligible (Ab)	
27	Shamsoddini A et al. (2026) [205]. Impact of virtual reality versus conventional balance training on balance function and fear of falling in older adults: a single blinded, randomized controlled trial. Eur Geriatr Med. PMID 42223906; doi:10.1007/s41999-026-01493-z	Potentially eligible (Ab)	
28	Sheehy L et al. (2025) [206]. Non-Immersive Virtual Reality Exercise Can Increase Exercise in Older Adults Living in the Community and in Long-Term Care: A Randomized Controlled Trial. Clin Interv Aging. PMID 39931102; doi:10.2147/CIA.S498272	Potentially eligible (Ab)	Home-based sample only (facility sample ineligible)
29	Tollár J et al. (2019) [207]. Diverse Exercises Similarly Reduce Older Adults' Mobility Limitations. Med Sci Sports Exerc. PMID 30973482; doi:10.1249/MSS.0000000000002001	Potentially eligible (Ab)	Mobility-limited older adults; three arms
30	Toulotte C et al. (2012) [208]. Wii Fit® training vs. Adapted Physical Activities: which one is the most appropriate to improve the balance of independent senior subjects? A randomized controlled study. Clin Rehabil. PMID 22324055; doi:10.1177/0269215511434996	Potentially eligible (Ab)	
31	Yang CM et al. (2020) [209]. Effects of Kinect exergames on balance training among community older adults: A randomized controlled trial. Medicine (Baltimore). PMID 32664177; doi:10.1097/MD.0000000000021228	Potentially eligible (Ab)	
32	Yang DJ et al. (2026) [210]. Exergame-based cognitive-motor integrated training: A novel approach to improving physical functions in older adults. Hong Kong Physiother J. PMID 42016049; doi:10.1142/S1013702526500010	Potentially eligible (Ab)	
33	Yang DJ et al. (2026) [211]. Effects of a 3D motion recognition-based virtual training system on balance and gait performance in older adults. Phys Act Nutr. PMID 41956492; doi:10.20463/pan.2026.0002	Potentially eligible (Ab)	
34	Yue T et al. (2025) [212]. Effects of Immersive Virtual Reality-Based Tennis Training on Balance in Older Adults. Gerontology. PMID 40695260; doi:10.1159/000547548	Potentially eligible (Ab)	Sham VR documentary control
35	Anson E et al. (2018) [213]. Trunk motion visual feedback during walking improves dynamic balance in older adults: Assessor blinded randomized controlled trial. Gait Posture. PMID 29614467; doi:10.1016/j.gaitpost.2018.03.044	Unclear	Treadmill walking with cursor feedback: confirm VR definition
36	Béraud-Peigné N et al. (2024) [214]. The effects of a new immersive multidomain training on cognitive, dual-task and physical functions in older adults. Geroscience. PMID 37743415; doi:10.1007/s11357-023-00952-w	Unclear	Allocation method not stated in abstract
37	Cho GH et al. (2014) [215]. The Effects of Virtual Reality-based Balance Training on Balance of the Elderly. J Phys Ther Sci. PMID 24764645; doi:10.1589/jpts.26.615	Unclear	Randomisation not stated in abstract
38	Dos Santos RG et al. (2023) [216]. Effects of twelve weeks of dance Exergames on the quality-of-life variables of elderly Brazilian women enrolled in a community program. J Bodyw Mov Ther. PMID 37949598; doi:10.1016/j.jbmt.2023.04.056	Unclear	Allocation method not stated in abstract
39	Jiang Y et al. (2025) [217]. Effectiveness of an Artificial Intelligence-Infused SinDance Exergame for Enhancing Physical Functions and Well-Being in Older Adults: A Pilot Randomized Controlled Trial. J Am Med Dir Assoc. PMID 40456279; doi:10.1016/j.jamda.2025.105701	Unclear	No abstract
40	Lee Y et al. (2017) [218]. Virtual Reality Training With Three-Dimensional Video Games Improves Postural Balance and Lower Extremity Strength in Community-Dwelling Older Adults. J Aging Phys Act. PMID 28290746; doi:10.1123/japa.2015-0271	Unclear	Randomisation not stated in abstract
41	Maillot P et al. (2012) [219]. Effects of interactive physical-activity video-game training on physical and cognitive function in older adults. Psychol Aging. PMID 22122605; doi:10.1037/a0026268	Unclear	Randomisation not stated in abstract
42	Singh Y et al. (2026) [220]. Visuomotor coordination training in dual-motor task improves balance recovery while responding to visuospatial perturbations in older adults. Hum Mov Sci. PMID 42607526; doi:10.1016/j.humov.2026.103527	Unclear	Allocation method not stated in abstract
43	Sápi M et al. (2019) [221]. Is Kinect Training Superior to Conventional Balance Training for Healthy Older Adults to Improve Postural Control? Games Health J. PMID 30153062; doi:10.1089/g4h.2018.0027	Unclear	Allocation method not stated in abstract

No.	Report	Status	Notes
44	Uematsu A et al. (2023) [222]. Effects of Motor-Cognitive Dual-Task Standing Balance Exergaming Training on Healthy Older Adults' Standing Balance and Walking Performance. Games Health J. PMID 36944151; doi:10.1089/g4h.2022.0203	Unclear	Allocation method not stated in abstract
45	Abd El-Kafy EM et al. (2024) [223]. C-Mill Virtual Reality/Augmented Reality Treadmill Training for Reducing Risk of Fall in the Elderly: A Randomized Controlled Trial. Games Health J. PMID 38563663; doi:10.1089/g4h.2023.0162	Companion	Possibly same trial as C-Mill 2024 (#187)
46	Chan WLS et al. (2024) [224]. A randomised controlled pilot study of a Nintendo Ring Fit Adventure™ balance and strengthening exercise program in community-dwelling older adults with a history of falls. Australas J Ageing. PMID 38404233; doi:10.1111/ajag.13297	Companion	Second report of Ring Fit trial (#202)
47	Khushnood K et al. (2021) [225]. Role Wii Fit exer-games in improving balance confidence and quality of life in elderly population. J Pak Med Assoc. PMID 34580500; doi:10.47391/JPMA.319	Companion	Second report of Rahman 2021 trial
48	Maillot P et al. (2014) [226]. The braking force in walking: age-related differences and improvement in older adults with exergame training. J Aging Phys Act. PMID 24231655; doi:10.1123/japa.2013-0001	Companion	Same trial as Maillot 2012 (#19)
